# Correction to *Lancet Glob Health* 2022; 10: 1715–43

**DOI:** 10.1016/S2214-109X(24)00036-6

**Published:** 2024-01-17

*GBD 2019 Healthcare Access and Quality Collaborators. Assessing performance of the Healthcare Access and Quality Index, overall and by select age groups, for 204 countries and territories, 1990–2019: a systematic analysis from the Global Burden of Disease Study 2019.* Lancet Glob Health *2022;* 10: *1715–43*—In this Article, Ashok Bhurtyal should have been included in the list of collaborators. The author contributions section of the appendix has also been updated accordingly. These corrections have been made as of Jan 17, 2024.

