# Supplementary material for: Assessing performance of the Healthcare Access and Quality Index, overall and by select age groups, for 204 countries and territories, 1990–2019: a systematic analysis from the Global Burden of Disease Study 2019
Source: Lancet Glob Health. 2022 Oct 6;10(12):e1715–43. doi: 10.1016/S2214-109X(22)00429-6 (PMC9666426; doi:10.1016/S2214-109X(22)00429-6)
Supplement: Supplementary appendix [file mmc1.pdf]

THE LANCET
Global Health

Supplementary appendix

This appendix formed part of the original submission and has been peer reviewed. We post it as supplied by the authors.

Supplement to: GBD 2019 Healthcare Access and Quality Collaborators. Assessing performance of the Healthcare Access and Quality Index, overall and by select age groups, for 204 countries and territories, 1990–2019: a systematic analysis from the Global Burden of Disease Study 2019. *Lancet Glob Health* 2022; published online Oct 6. https://doi.org/10.1016/S2214-109X(22)00429-6.

Appendix to Assessing performance on the Healthcare Access and Quality Index, overall and by select age group, for 204 countries and territories, 1990–2019: a systematic analysis from the Global Burden of Disease Study 2019

This appendix provides further methodological detail and supplemental figures and tables.

Table of Contents

Author Contributions 3
Preamble 8
List of supplementary figures and tables 9
GATHER Statement 11
Appendix Table 1 GATHER Checklist 12
Part 1. Estimating the Healthcare Access and Quality Index 14
Overview 14
Section 1. Healthcare Access and Quality Index overview 14
Causes of amenable mortality 14
Section 2. Overview of modelling strategy for the Healthcare Access and Quality Index 15
Modelling overview 15
Inputs and modelling strategies 15
Risk-standardisation 16
Mortality-incidence ratios 17
Age-standardisation 21
Scaling Causes 21
Creating the HAQ Index 21
Uncertainty analysis 30
Part 2. Estimating convergence 31
Beta convergence 31
Sigma convergence 34
Convergence with High SDI quintile countries 35
Part 3. Online tools and glossary of terms 40
Section 1. Online tools 41
Section 2. List of abbreviations 41
Section 3. List of ISO3 codes and location names 41
Section 4. SDI reference quintiles and country-specific SDI values 50
References 58
Part 4. Cause write-ups 59

Author Contributions

Managing the overall research enterprise

Kelly Bienhoff, Annie Haakenstad, Kate E LeGrand, Stephen S Lim, Rafael Lozano, and Christopher J L Murray.

Writing the first draft of the manuscript

Nancy Fullman, Annie Haakenstad, Rafael Lozano, and Jamal Akeem Yearwood.

Primary responsibility for applying analytical methods to produce estimates

Corinne Bintz, Nancy Fullman, Annie Haakenstad, Jonah N Joffe, Stephen S Lim, Rafael Lozano, Christopher J L Murray, Vishnu Nandakumar , Marcia R Weaver, and Jamal Akeem Yearwood.

Primary responsibility for seeking, cataloguing, extracting, or cleaning data; designing or coding figures and tables

Corinne Bintz, Megan Knight, and Jamal Akeem Yearwood.

Providing data or critical feedback on data sources

Cristiana Abbafati, Amir Abdoli, Victor Adekanmbi, Olatunji O Adetokunboh, Muhammad Sohail Afzal, Saira Afzal, Marcela Agudelo-Botero, Bright Opoku Ahinkorah, Sajjad Ahmad, Ali Ahmadi, Sepideh Ahmadi, Tarik Ahmed Rashid, Budi Aji, Hanadi Al Hamad, Liaqat Ali, Syed Mohamed Aljunid, Dickson A Amugsi, Jalal Arabloo, Judie Arulappan, Desta Debalkie Atnafu, Alok Atreya, Martin Amogre Ayanore, Samad Azari, Atif Amin Baig, Shankar M Bakkannavar, Palash Chandra Banik, Till Winfried Bärnighausen, Amadou Barrow, Sanjay Basu, Akshaya Srikanth Bhagavathula, Sonu Bhaskar, Sadia Bibi, Boris Bikbov, Obasanjo Afolabi Bolarinwa, Katrin Burkart, Nadeem Shafique Butt, Lucero Cahuana-Hurtado, Luis Alberto Cámera, Ferrán Catalá-López, Massimo Cirillo, Xiaochen Dai, Lalit Dandona, Rakhi Dandona, William James Dangel, Mandira Lamichhane Dhimal, Meghnath Dhimal, Huyen Phuc Do, Leila Doshmangir, Bruce B Duncan, Andem Effiong, Islam Y Elgendy, Jawad Fares, Farshad Farzadfar, Takeshi Fukumoto, Peter Andras Gaal, William M Gardner, Mansour Ghafourifard, Ahmad Ghashghaee, Mahaveer Golechha, Rajat Das Gupta, Vivek Kumar Gupta, Annie Haakenstad, Mohammad Hamiduzzaman, Josep Maria Haro, Ahmed I Hasaballah, Khezar Hayat, Mohammad Heidari, Claudiu Herteliu, Sheikh Jamal Hossain, Mehdi Hosseinzadeh, Olayinka Stephen Ilesanmi, Seyed Sina Naghibi Irvani, Nahlah Elkudssiah Ismail, Gaetano Isola, Nader Jahanmehr, Rajesh Jain, Mihajlo Jakovljevic, Sathish Kumar Jayapal, Shubha Jayaram, Jost B Jonas, Tamas Joo, Mikk Jürisson, Leila R Kalankesh, Himal Kandel, Rami S Kantar, Srinivasa Vittal Katikireddi, Taras Kavetskyy, Norito Kawakami, Gbenga A Kayode, Leila Keikavoosi-Arani, Yousef Saleh Khader, Himanshu Khajuria, Rovshan Khalilov, Mohammad Khammarnia, Maseer Khan, Md Nuruzzaman Khan, Moien AB Khan, Min Seo Kim, Yun Jin Kim, Adnan Kisa, Sezer Kisa, Soewarta Kosen, Barthelemy Kuate Defo, G Anil Kumar, Dian Kusuma, Hmwe Hmwe Kyu, Savita Lasrado, Yo Han Lee, Rafael Lozano, Alaa Makki, Reza Malekzadeh, Deborah Carvalho Malta, Mohammad Ali Mansournia, Lorenzo Giovanni Mantovani, Francisco Rogerlândio Martins-Melo, Walter Mendoza, Ritesh G Menezes, Atte Meretoja, Erkin M Mirrakhimov, Awoke Misganaw, Babak Moazen, Mokhtar Mohammadi, Shafiu Mohammed, Modhurima Moitra, Ali H Mokdad, Mariam Molokhia, Lorenzo Monasta, Ghobad Moradi, Jonathan F Mosser, Ebrahim Mostafavi, Ahamarshan Jayaraman Nagarajan, Mohsen Naghavi, Sreenivas Narasimha Swamy, Aparna Ichalangod Narayana, Bruno Ramos Nascimento, Biswa Prakash Nayak, Javad Nazari, Ionut Negoi, Sandhya Neupane Kandel, Josephine W Ngunjiri, Cuong Tat Nguyen, Huong Lan Thi Nguyen, Jean Jacques Noubiap, Bogdan Oancea, Onome

Bright Oghenetega, Andrew T Olagunju, Ahmed Omar Bali, Emad Omer, Obinna E Onwujekwe, Adrian Otoiu, Jagadish Rao Padubidri, Adrian Pana, Songhomitra Panda-Jonas, Shahina Pardhan, Deepak Kumar Pasupula, Praveen Kumar Pathak, Shrikant Pawar, Jeevan Pereira, Vivek Podder, Maarten J Postma, Zahiruddin Quazi Syed, Navid Rabiee, Raghu Anekal Radhakrishnan, Amir Masoud Rahmani, Chhabi Lal Ranabhat, Chythra R Rao, Sowmya J Rao, Salman Rawaf, Lal Rawal, Andre M N Renzaho, Bhageerathy Reshmi, Seyed Mohammad Riahi, Rezaul Karim Ripon, Umar Saeed, Harihar Sahoo, Abdallah M Samy, Milena M Santric-Milicevic, Brijesh Sathian, Monika Sawhney, Maria Inês Schmidt, Abdul-Aziz Seidu, Sadaf G Sepanlou, Allen Seylani, Masood Ali Shaikh, Adithi Shetty, K M Shivakumar, Jasvinder A Singh, Valentin Yurievich Skryabin, Anna Aleksandrovna Skryabina, Jing Sun, Miklós Szócska, Rafael Tabarés-Seisdedos, Hooman Tadbiri, Roman Topor-Madry, Marcos Roberto Tovani-Palone, Bach Xuan Tran, Mai Thi Ngoc Tran, Niharika Tripathi, Jaya Prasad Tripathy, Christopher E Troeger, Deinzel Robles Uezono, Anayat Ullah, Saif Ullah, Bhaskaran Unnikrishnan, Sahel Valadan Tahbaz, Pascual R Valdez, Vasily Vlassov, Bay Vo, Yasir Waheed, Marcia R Weaver, Taweewat Wiangkham, Seyed Hossein Yahyazadeh Jabbari, Kazumasa Yamagishi, Sanni Yaya, Jamal Akeem Yearwood, Siyan Yi, Vahit Yiğit, Naohiro Yonemoto, Mustafa Z Younis, Chuanhua Yu, Mikhail Sergeevich Zastrozhin, and Yves Miel H Zuniga.

Developing methods or computational machinery

Saira Afzal, Ali Ahmadi, Tarik Ahmed Rashid, Liaqat Ali, Jalal Arabloo, Zahra Aryan, Corinne Bintz, Xiaochen Dai, Iman El Sayed, William M Gardner, Annie Haakenstad, Mehdi Hosseinzadeh, Jonah N Joffe, Taras Kavetskyy, Leila Keikavoosi-Arani, Rovshan Khalilov, Mohammad Khammarnia, Adnan Kisa, Sezer Kisa, Megan Knight, Rafael Lozano, Alaa Makki, Seyedeh Zahra Masoumi, Mokhtar Mohammadi, Shafiu Mohammed, Ali H Mokdad, Mohsen Naghavi, Vishnu Nandakumar , Josephine W Ngunjiri, Ahmed Omar Bali, Emad Omer, Zahiruddin Quazi Syed, Amir Masoud Rahmani, Chhabi Lal Ranabhat, Lal Rawal, Umar Saeed, Abdallah M Samy, Roman Topor-Madry, Mai Thi Ngoc Tran, Niharika Tripathi, Christopher E Troeger, Bay Vo, Vahid Yazdi-Feyzabadi, Jamal Akeem Yearwood, and Mikhail Sergeevich Zastrozhin.

Providing critical feedback on methods or results

Cristiana Abbafati, Roberto Ariel Abeldaño Zuñiga, Victor Adekanmbi, Olatunji O Adetokunboh, Muhammad Sohail Afzal, Saira Afzal, Marcela Agudelo-Botero, Bright Opoku Ahinkorah, Sajjad Ahmad, Ali Ahmadi, Ali Ahmed, Tarik Ahmed Rashid, Budi Aji, Wuraola Akande-Sholabi, Hanadi Al Hamad, Khurshid Alam, Robert Kaba Alhassan, Liaqat Ali, Vahid Alipour, Syed Mohamed Aljunid, Edward Kwabena Ameyaw, Tarek Tawfik Amin, Dickson A Amugsi, Robert Ancuceanu, Pedro Prata Andrade, Afifa Anjum, Jalal Arabloo, Morteza Arab-Zozani, Hany Ariffin, Judie Arulappan, Zahra Aryan, Alok Atreya, Marcel Ausloos, Leticia Avila-Burgos, Getinet Ayano, Martin Amogre Ayanore, Samad Azari, Ashish D Badiye, Atif Amin Baig, Mohan Bairwa, Shrikala Baliga, Palash Chandra Banik, Till Winfried Bärnighausen, Amadou Barrow, Sanjay Basu, Mohsen Bayati, Rebuma Belete, Arielle Wilder Bell, Devidas S Bhagat, Akshaya Srikanth Bhagavathula, Nikha Bhardwaj, Pankaj Bhardwaj, Sonu Bhaskar, Krittika Bhattacharyya, Zulfiqar A Bhutta, Sadia Bibi, Ali Bijani, Boris Bikbov, Obasanjo Afolabi Bolarinwa, Aime Bonny, Hermann Brenner, Danilo Buonsenso, Katrin Burkart, Reinhard Busse, Nadeem Shafique Butt, Zahid A Butt, Florentino Luciano Caetano dos Santos, Lucero Cahuana-Hurtado, Rosario Cárdenas, Vera L A Carneiro, Ferrán Catalá-López, Jaykaran Charan, Prachi P Chavan, Shu Chen, Sonali Gajanan Choudhari, Enayet Karim Chowdhury, Mohiuddin Ahsanul Kabir Chowdhury, Barbara Corso, Omid Dadras, Saad M A Dahlawi, Xiaochen Dai, Lalit Dandona, Rakhi Dandona, William James Dangel, Claudio Alberto Dávila-Cervantes, Kairat Davletov, Keshab Deuba, Mandira Lamichhane Dhimal, Meghnath Dhimal, Shirin Djalalinia, Huyen Phuc Do, Leila Doshmangir, Andem Effiong, Elham Ehsani-Chimeh, Iman El Sayed, Islam

Y Elgendy, Muhammed Elhadi, Daniel Asfaw Erku, Sharareh Eskandarieh, Jawad Fares, Farshad Farzadfar, Florian Fischer, Nataliya A Foigt, Masoud Foroutan, Takeshi Fukumoto, Nancy Fullman, Peter Andras Gaal, Santosh Gaihre, Tushar Garg, Abera Getachew Obsa, Mansour Ghafourifard, Ahmad Ghashghaee, Nermin Ghith, Syed Amir Gilani, Paramjit Singh Gill, Salime Goharinezhad, Mahaveer Golechha, Jenny S Guadamuz, Yuming Guo, Rajat Das Gupta, Rajeev Gupta, Vivek Kumar Gupta, Annie Haakenstad, Mohammad Hamiduzzaman, Asif Hanif, Ahmed I Hasaballah, M Tasdik Hasan, Md. Mehedi Hasan, Abdiwahab Hashi, Simon I Hay, Khezar Hayat, Golnaz Heidari, Mohammad Heidari, Nathaniel J Henry, Claudiu Herteliu, Ramesh Holla, Mohammad Bellal Hossain Hossain, Sahadat Hossain, Sheikh Jamal Hossain, Mehdi Hosseinzadeh, Soodabeh Hoveidamanesh, Vivian Chia-rong Hsieh, Guoqing Hu, M Mamun Huda, Susan C Ifeagwu, Kevin S Ikuta, Olayinka Stephen Ilesanmi, Seyed Sina Naghibi Irvani, Rakibul M Islam, Nahlah Elkudssiah Ismail, Gaetano Isola, Ramaiah Itumalla, Masao Iwagami, Mohammad Ali Jahani, Mihajlo Jakovljevic, Sathish Kumar Jayapal, Shubha Jayaram, Ravi Prakash Jha, Jonah N Joffe, Jost B Jonas, Tamas Joo, Nitin Joseph, Mikk Jürisson, Ali Kabir, Leila R Kalankesh, Rohollah Kalhor, Aruna M Kamath, Kaloyan Kamenov, Himal Kandel, Rami S Kantar, Neeti Kapoor, Marina Karanikolos, Srinivasa Vittal Katikireddi, Taras Kavetskyy, Norito Kawakami, Gbenga A Kayode, Leila Keikavoosi-Arani, Mohammad Keykhaei, Yousef Saleh Khader, Himanshu Khajuria, Rovshan Khalilov, Mohammad Khammarnia, Maseer Khan, Md Nuruzzaman Khan, Moien AB Khan, Min Seo Kim, Yun Jin Kim, Adnan Kisa, Sezer Kisa, Vitalii Klymchuk, Megan Knight, Kamrun Nahar Koly, Oleksii Korzh, Barthelemy Kuate Defo, G Anil Kumar, Dian Kusuma, Hmwe Hmwe Kyu, Anders O Larsson, Savita Lasrado, Chiachi Bonnie Lee, Wei-Chen Lee, Yo Han Lee, Shanshan Li, Stephen S Lim, Rafael Lozano, Giancarlo Lucchetti, Preetam Bhalchandra Mahajan, Azeem Majeed, Alaa Makki, Reza Malekzadeh, Ahmad Azam Malik, Mohammad Ali Mansournia, Adolfo Martinez-Valle, Francisco Rogerlândio Martins-Melo, Manu Raj Mathur, Richard James Maude, Martin McKee, Walter Mendoza, Ritesh G Menezes, George A Mensah, Atte Meretoja, Tomislav Mestrovic, Irmina Maria Michalek, Erkin M Mirrakhimov, Sanjeev Misra, Babak Moazen, Mokhtar Mohammadi, Shafiu Mohammed, Modhurima Moitra, Ali H Mokdad, Mariam Molokhia, Mohammad Ali Moni, Ghobad Moradi, Rafael Silveira Moreira, Jonathan F Mosser, Ebrahim Mostafavi, Simin Mouodi, Christopher J L Murray, Ahamarshan Jayaraman Nagarajan, Chie Nagata, Mohsen Naghavi, Vinay Nangia, Sreenivas Narasimha Swamy, Bruno Ramos Nascimento, Biswa Prakash Nayak, Javad Nazari, Ionut Negoi, Samata Nepal, Sandhya Neupane Kandel, Josephine W Ngunjiri, Cuong Tat Nguyen, Huong Lan Thi Nguyen, Dina Nur Anggraini Ningrum, Jean Jacques Noubiap, Bogdan Oancea, Onome Bright Oghenetega, Andrew T Olagunju, Ahmed Omar Bali, Emad Omer, Obinna E Onwujekwe, Adrian Otoiu, Jagadish Rao Padubidri, Raffaele Palladino, Adrian Pana, Songhomitra Panda-Jonas, Seithikurippu R Pandi-Perumal, Shahina Pardhan, Deepak Kumar Pasupula, Praveen Kumar Pathak, Shrikant Pawar, Jeevan Pereira, Manju Pilania, Bakhtiar Piroozi, Vivek Podder, Khem Narayan Pokhrel, Maarten J Postma, Zahiruddin Quazi Syed, Navid Rabiee, Mahfuzar Rahman, Md. Mosfequr Rahman, Mohammad Hifz Ur Rahman, Mosiur Rahman, Amir Masoud Rahmani, Chhabi Lal Ranabhat, Chythra R Rao, Sowmya J Rao, David Laith Rawaf, Salman Rawaf, Lal Rawal, Andre M N Renzaho, Bhageerathy Reshmi, Serge Resnikoff, Aziz Rezapour, Seyed Mohammad Riahi, Rezaul Karim Ripon, Masoumeh Sadeghi, Umar Saeed, Biniyam Sahiledengle, Harihar Sahoo, Maitreyi Sahu, Joseph S Salama, Payman Salamati, Abdallah M Samy, Juan Sanabria, Milena M Santric-Milicevic, Brijesh Sathian, Monika Sawhney, Abdul-Aziz Seidu, Sadaf G Sepanlou, Allen Seylani, Masood Ali Shaikh, Aziz Sheikh, Mika Shigematsu, Rahman Shiri, K M Shivakumar, Jasvinder A Singh, Dhirendra Narain Sinha, Valentin Yurievich Skryabin, Anna Aleksandrovna Skryabina, Ahmad Sofi-Mahmudi, Jacqueline H Stephens, Jing Sun, Miklós Szócska, Rafael Tabarés-Seisdedos, Hooman Tadbiri, Animut Tagele Tamiru, Kavumpurathu

Raman Thankappan, Roman Topor-Madry, Marcos Roberto Tovani-Palone, Bach Xuan Tran, Mai Thi Ngoc Tran, Niharika Tripathi, Jaya Prasad Tripathy, Christopher E Troeger, Deinzel Robles Uezono, Anayat Ullah, Saif Ullah, Bhaskaran Unnikrishnan, Sahel Valadan Tahbaz, Pascual R Valdez, Milena Vasic, Massimiliano Veroux, Dominique Vervoort, Francesco S Violante, Sergey Konstantinovitch Vladimirov, Bay Vo, Yasir Waheed, Richard G Wamai, Yanzhong Wang, Paul Ward, Taweewat Wiangkham, Lalit Yadav, Seyed Hossein Yahyazadeh Jabbari, Sanni Yaya, Jamal Akeem Yearwood, Siyan Yi, Vahit Yiğit, Naohiro Yonemoto, Mustafa Z Younis, Chuanhua Yu, Ismaeel Yunusa, Sojib Bin Zaman, Mikhail Sergeevich Zastrozhin, Chenwen Zhong, and Yves Miel H Zuniga.

Drafting the work or revising is critically for important intellectual content

Cristiana Abbafati, Mohsen Abbasi-Kangevari, Roberto Ariel Abeldaño Zuñiga, Isaac Akinkunmi Adedeji, Victor Adekanmbi, Olatunji O Adetokunboh, Muhammad Sohail Afzal, Saira Afzal, Bright Opoku Ahinkorah, Ali Ahmadi, Sepideh Ahmadi, Ali Ahmed, Khurshid Alam, Robert Kaba Alhassan, Liaqat Ali, Tarek Tawfik Amin, Hubert Amu, Dickson A Amugsi, Robert Ancuceanu, Pedro Prata Andrade, Afifa Anjum, Jalal Arabloo, Morteza Arab-Zozani, Hany Ariffin, Judie Arulappan, Tahira Ashraf, Alok Atreya, Leticia Avila-Burgos, Ashish D Badiye, Atif Amin Baig, Shrikala Baliga, Palash Chandra Banik, Till Winfried Bärnighausen, Fabio Barra, Amadou Barrow, Sanjay Basu, Rebuma Belete, Arielle Wilder Bell, Akshaya Srikanth Bhagavathula, Sonu Bhaskar, Krittika Bhattacharyya, Sadia Bibi, Kelly Bienhoff, Boris Bikbov, Antonio Biondi, Obasanjo Afolabi Bolarinwa, Hermann Brenner, Danilo Buonsenso, Florentino Luciano Caetano dos Santos, Lucero Cahuana-Hurtado, Vera L A Carneiro, Ferrán Catalá-López, Joht Singh Chandan, Simiao Chen, Barbara Corso, Claudio Alberto Dávila-Cervantes, Mandira Lamichhane Dhimal, Meghnath Dhimal, Huyen Phuc Do, Leila Doshmangir, Bruce B Duncan, Andem Effiong, Iman El Sayed, Maha El Tantawi, Islam Y Elgendy, Muhammed Elhadi, Sharareh Eskandarieh, Jawad Fares, Simone Ferrero, Lorenzo Ferro Desideri, Florian Fischer, Masoud Foroutan, Takeshi Fukumoto, Nancy Fullman, Peter Andras Gaal, Santosh Gaihre, Tushar Garg, Abera Getachew Obsa, Mansour Ghafourifard, Nermin Ghith, Paramjit Singh Gill, Jenny S Guadamuz, Rajeev Gupta, Veer Bala Gupta, Vivek Kumar Gupta, Mohammad Hamiduzzaman, Josep Maria Haro, Ahmed I Hasaballah, M Tasdik Hasan, Abdiwahab Hashi, Simon I Hay, Golnaz Heidari, Claudiu Herteliu, Ramesh Holla, Mohammad Bellal Hossain Hossain, Sahadat Hossain, Sheikh Jamal Hossain, Sorin Hostiuc, Vivian Chia-rong Hsieh, Guoqing Hu, Junjie Huang, M Mamun Huda, Olayinka Stephen Ilesanmi, Seyed Sina Naghibi Irvani, Rakibul M Islam, Sheikh Mohammed Shariful Islam, Nahlah Elkudssiah Ismail, Hiroyasu Iso, Gaetano Isola, Ramaiah Itumalla, Mohammad Ali Jahani, Nader Jahanmehr, Rajesh Jain, Mihajlo Jakovljevic, Manthan Dilipkumar Janodia, Sathish Kumar Jayapal, Shubha Jayaram, Ravi Prakash Jha, Jost B Jonas, Tamas Joo, Nitin Joseph, Mikk Jürisson, Ali Kabir, Himal Kandel, Rami S Kantar, Neeti Kapoor, Marina Karanikolos, Srinivasa Vittal Katikireddi, Taras Kavetskyy, Gbenga A Kayode, Leila Keikavoosi-Arani, Yousef Saleh Khader, Rovshan Khalilov, Mohammad Khammarnia, Maseer Khan, Md Nuruzzaman Khan, Moien AB Khan, Mehdi Khezeli, Yun Jin Kim, Adnan Kisa, Sezer Kisa, Megan Knight, Kamrun Nahar Koly, Oleksii Korzh, Parvaiz A Koul, Barthelemy Kuate Defo, Dian Kusuma, Anders O Larsson, Savita Lasrado, Kate E LeGrand, Rafael Lozano, Giancarlo Lucchetti, Preetam Bhalchandra Mahajan, Reza Malekzadeh, Ahmad Azam Malik, Deborah Carvalho Malta, Lorenzo Giovanni Mantovani, Adolfo Martinez-Valle, Francisco Rogerlândio Martins-Melo, Richard James Maude, Pallab K Maulik, Walter Mendoza, Ritesh G Menezes, George A Mensah, Atte Meretoja, Tuomo J Meretoja, Tomislav Mestrovic, Irmina Maria Michalek, Babak Moazen, Shafiu Mohammed, Ali H Mokdad, Mariam Molokhia, Lorenzo Monasta, Mohammad Ali Moni, Rafael Silveira Moreira, Jonathan F Mosser, Ebrahim Mostafavi, Simin Mouodi, Christopher J L Murray, Ahamarshan Jayaraman Nagarajan, Mohsen Naghavi, Sreenivas Narasimha Swamy, Aparna Ichalangod

Narayana, Bruno Ramos Nascimento, Hasan Nassereldine, Biswa Prakash Nayak, Javad Nazari, Ionut Negoi, Samata Nepal, Sandhya Neupane Kandel, Josephine W Ngunjiri, Cuong Tat Nguyen, Huong Lan Thi Nguyen, Onome Bright Oghenetega, In-Hwan Oh, Andrew T Olagunju, Babayemi Oluwaseun Olakunde, Obinna E Onwujekwe, Adrian Otoiu, Jagadish Rao Padubidri, Songhomitra Panda-Jonas, Seithikurippu R Pandi-Perumal, Shahina Pardhan, Praveen Kumar Pathak, George C Patton, Shrikant Pawar, Jeevan Pereira, Vivek Podder, Maarten J Postma, Sergio I Prada, Zahiruddin Quazi Syed, Navid Rabiee, Mahfuzar Rahman, Md. Mosfequr Rahman, Chhabi Lal Ranabhat, Chythra R Rao, Sowmya J Rao, Davide Rasella, David Laith Rawaf, Salman Rawaf, Lal Rawal, Andre M N Renzaho, Bhageerathy Reshmi, Seyed Mohammad Riahi, Simona Sacco, Umar Saeed, Amirhossein Sahebkar, Biniyam Sahiledengle, Harihar Sahoo, Maitreyi Sahu, Joseph S Salama, Abdallah M Samy, Juan Sanabria, Milena M Santric-Milicevic, Maria Inês Schmidt, Allen Seylani, Aziz Sheikh, Mika Shigematsu, K M Shivakumar, Jasvinder A Singh, Dhirendra Narain Sinha, Valentin Yurievich Skryabin, Anna Aleksandrovna Skryabina, Ahmad Sofi-Mahmudi, Raúl A R C Sousa, Jacqueline H Stephens, Miklós Szócska, Hooman Tadbiri, Animut Tagele Tamiru, Roman Topor-Madry, Marcos Roberto Tovani-Palone, Bach Xuan Tran, Mai Thi Ngoc Tran, Niharika Tripathi, Anayat Ullah, Saif Ullah, Bhaskaran Unnikrishnan, Marco Vacante, Massimiliano Veroux, Francesco S Violante, Vasily Vlassov, Yanzhong Wang, Yuan-Pang Wang, Paul Ward, Marcia R Weaver, Taweewat Wiangkham, Lalit Yadav, Kazumasa Yamagishi, Sanni Yaya, Vahid Yazdi-Feyzabadi, Jamal Akeem Yearwood, Vahit Yiğit, Naohiro Yonemoto, Sojib Bin Zaman, Mikhail Sergeevich Zastrozhin, and Zhi-Jiang Zhang.

Managing the estimation or publications process

Cristiana Abbafati, Isaac Akinkunmi Adedeji, Marcela Agudelo-Botero, Sepideh Ahmadi, Devidas S Bhagat, Ahmed I Hasaballah, Rakibul M Islam, Nader Jahanmehr, Rami S Kantar, Wei-Chen Lee, Shanshan Li, Ali H Mokdad, Ahamarshan Jayaraman Nagarajan, Vinay Nangia, Ionut Negoi, Navid Rabiee, Bach Xuan Tran, Jaya Prasad Tripathy, Naohiro Yonemoto, and Yves Miel H Zuniga.

Preamble

This appendix provides methodological detail for estimating the Healthcare Access and Quality Index as well as supplementary results. The appendix is organized into broad sections following the structure of the main paper. This study complies with the Guidelines for Accurate and Transparent Health Estimates Reporting (GATHER) recommendations. It includes detailed indicator modeling write-ups and flowcharts, and information on data sourcing to maximize transparency in our estimation processes and provides a comprehensive account of analytical steps. We intend this to be a living document, to be updated with each annual iteration of the Global Burden of Diseases, Injuries, and Risk Factors Study (GBD).

List of supplementary figures and tables

Appendix Figure 1. Reference information for cause of death methods

Appendix Figure 2a. HAQ PCA Weighted Overall Index (0-74) vs Mean Overall Index (0-74), 2017

Appendix Figure 2b. HAQ PCA Weighted Young Index (0-14) vs Mean Young Index (0-14), 2017

Appendix Figure 2c. HAQ PCA Weighted Working Index (15-64) vs Mean Working Index (15-64), 2017

Appendix Figure 2d. HAQ PCA Weighted Post-working Index (65-74) vs Mean Post-working Index (65-74), 2017

Appendix Figure 3a. HAQ Geometric Mean Overall Index (0-74) vs Arithmetic Mean Overall Index (0-74), 2019

Appendix Figure 3b. HAQ Geometric Mean Young Index (0-14) vs Arithmetic Mean Young Index (0-14), 2019

Appendix Figure 3c. HAQ Geometric Mean Working Index (15-64) vs Arithmetic Mean Working Index (15-64), 2019

Appendix Figure 3d. HAQ Geometric Mean Post-working Index (65-74) vs Arithmetic Mean Post-working Index (65-74), 2019

Appendix Figure 4: Absolute change in HAQ Index, 1990-2019 versus 1990 HAQ Index by select age group

Appendix Figure 5. Annualized percent change in HAQ Index, 1990-2019, versus 1990 Overall HAQ by select age group

Appendix Figure 6a. Trends in average gap with mean HAQ Index in the High SDI Quintile by select age group, Low SDI quintile, 1990-2019

Appendix Figure 6b. Trends in average gap with mean HAQ Index in the High SDI Quintile by select age group, Low-middle SDI quintile, 1990-2019

Appendix Figure 6c. Trends in average gap with mean HAQ Index in the High SDI Quintile by select age group, Middle SDI quintile, 1990-2019

Appendix Figure 6d. Trends in average gap with mean HAQ Index in the High SDI Quintile by select age group, High-middle SDI quintile, 1990-2019

Appendix Table 1. GATHER checklist

Appendix Table 2. The correlation of MIRs and RSDs, respectively, with HALE, for causes tested

Appendix Table 3. Causes for which mortality is amenable to health care, mapped to GBD causes, and amenable age ranges.

Appendix Table 4a. Absolute change in HAQ Index, 1990 to 2019, by select age group

Appendix Table 4b. Annualized percent change in HAQ Index, 1990 to 2019, by select age group

Appendix Table 5. Normalized Standard Deviation HAQ, 1990 and 2019

Appendix Table 6. HAQ Index regressed on SDI over 1990-2019 by select age group

Appendix Table 7. SDI reference quintiles

GATHER Statement

This study complies with the Guidelines for Accurate and Transparent Health Estimates Reporting (GATHER) recommendations.[1] We have documented the steps involved in our analytical procedures and detailed the data sources used in compliance with the GATHER. For additional GATHER reporting, please refer to Appendix Table 1 on pages 7.

Appendix Table 1 GATHER Checklist

GATHER checklist of information that should be included in reports of global health estimates, with description of compliance and location of information

#	GATHER checklist item	Description of compliance	Reference
**Objectives and funding**			
**1**	Define the indicator(s), populations (including age, sex, and geographic entities), and time period(s) for which estimates were made.	Description of indicators, definitions, relevant time periods, and populations in paper and appendix.	Main Text Methods; Appendix, Part 1, Section 1; Appendix Table 3;
**2**	List the funding sources for the work.	Funding sources listed in paper.	Main Text Summary
**Data inputs**			
For all data inputs from multiple sources that are synthesized as part of the study:			
**3**	Describe how the data were identified and how the data were accessed.	Narrative description of data seeking methodology provided.	Appendix, Part 1, Section 1
**4**	Specify the inclusion and exclusion criteria. Identify all ad-hoc exclusions.	Narrative about inclusion and exclusion criteria by data type provided in linked materials.	Inclusion and exclusion criteria, and ad-hoc exclusions are included in cause-specific write-ups (Appendix, Part 4)
**5**	Provide information on all included data sources and their main characteristics. For each data source used, report reference information or contact name/institution, population represented, data collection method, year(s) of data collection, sex and age range, diagnostic criteria or measurement method, and sample size, as relevant.	An interactive, online data source tool that provides metadata for data sources by component, geography, cause, risk, or impairment.	Available upon publication at: https://ghdx.healthdata.org/gbd-2019/
**6**	Identify and describe any categories of input data that have potentially important biases (e.g., based on characteristics listed in item 5).	Summary of known biases included in paper	Main Text Methods
For data inputs that contribute to the analysis but were not synthesized as part of the study:			
**7**	Describe and give sources for any other data inputs.	An interactive, online data source tool that provides metadata for data sources by component, geography, cause, risk, or impairment.	Appendix Methods, Part 3; https://ghdx.healthdata.org/gbd-2019/
For all data inputs:			
**8**	Provide all data inputs in a file format from which data can be efficiently extracted (e.g., a spreadsheet as opposed to a PDF), including all relevant meta-data listed in item 5. For any data inputs that cannot be shared due to ethical or legal reasons, such as third-party ownership, provide a contact name or the	Downloads of input data are available through online tools, including data visualization tools and data query tools.	Appendix Methods, Part 3; https://ghdx.healthdata.org/gbd-2019/

	name of the institution that retains the right to the data.		
**Data analysis**			
**9**	Provide a conceptual overview of the data analysis method. A diagram may be helpful.	Flow diagrams of the overall methodological processes, as well as cause-specific modelling processes have been provided.	Main text methods, Appendix Methods, Part 1
**10**	Provide a detailed description of all steps of the analysis, including mathematical formulae. This description should cover, as relevant, data cleaning, data pre-processing, data adjustments and weighting of data sources, and mathematical or statistical model(s).	Provided in the methodological write-ups.	Main text methods, Appendix Methods, Part 1
**11**	Describe how candidate models were evaluated and how the final model(s) were selected.	Provided in the methodological write-ups.	Main text methods, Appendix Methods, Part 1
**12**	Provide the results of an evaluation of model performance, if done, as well as the results of any relevant sensitivity analysis.	Provided in the methodological write-ups.	Main text methods, Appendix Methods, Part 1
**13**	Describe methods for calculating uncertainty of the estimates. State which sources of uncertainty were, and were not, accounted for in the uncertainty analysis.	Provided in the methodological write-ups.	Main text methods
**14**	State how analytic or statistical source code used to generate estimates can be accessed.	Access statement provided.	Links to code will be updated at time of publication and can be found here: http://ghdx.healthdata.org/gbd-2019/
**Results and Discussion**			
**15**	Provide published estimates in a file format from which data can be efficiently extracted.	GBD 2019 results are available through online data visualization tools, the Global Health Data Exchange, and the online data query tool.	Available upon publication at: https://ghdx.healthdata.org/gbd-2019/
**16**	Report a quantitative measure of the uncertainty of the estimates (e.g., uncertainty intervals).	Uncertainty intervals are provided with all results.	Main Text, Results
**17**	Interpret results in light of existing evidence. If updating a previous set of estimates, describe the reasons for changes in estimates.		Main Text, Discussion
**18**	Discuss limitations of the estimates. Include a discussion of any modelling assumptions or data limitations that affect interpretation of the estimates.	Discussion of limitations provided in the main text.	Main Text, Methods and Discussion

Part 1. Estimating the Healthcare Access and Quality Index

Overview

This study drew on the results of the Global Burden of Diseases, Injuries, and Risk Factors Study (GBD 2019) to estimate Healthcare Access and Quality (HAQ) Index scores for 204 countries and territories for the period from 1990 to 2019. This HAQ analysis improves on earlier iterations[2,3] by assessing variation in HAQ by age over time. This study drew on the results of the Global Burden of Diseases, Injuries, and Risk Factors Study (GBD 2019) to estimate Healthcare Access and Quality (HAQ) Index scores for 204 countries and territories for the period from 1990 to 2019. This HAQ analysis improves on earlier iterations by assessing variation in HAQ by age over time.[2,3]

Many of the methodologies described below have been presented in prior HAQ Index studies and in other GBD publications.[2–7] The major methodological difference in the HAQ methodology this round included using the arithmetic mean rather than principal component analysis (PCA) to construct the composite index and special considerations related to constructing the HAQ Index for each of the three age groups.

Section 1. Healthcare Access and Quality Index overview

We used the measurement of amenable mortality to assess the accessibility and quality of health care worldwide. Our research process was focused on first creating a list of causes of amenable mortality and then transforming the indicators to ensure that results would be valid and comparable across locations.

Causes of amenable mortality

Instead of attempting to measure health-care access and quality by looking directly at service availability and quality variables, we sought to infer health-care access and quality by measuring a set of carefully selected health outcomes. This approach avoided the difficulty of attempting to identify, compare, and collect data for a standard set of health-care services across all of the locations in our analysis.

We drew on the work of Nolte and McKee to compile a list of causes of mortality that can be avoided with access to good quality health-care.[8–10] These are known as causes of amenable mortality. From the list produced by Nolte and McKee, we selected 32 causes of amenable mortality that we were able to map to the GBD cause hierarchy. Being able to identify corresponding causes of mortality in the GBD cause list was important in order to be able to use outputs from the most recent GBD research cycle.

As we note in the main text, the original list of causes of amenable mortality that Nolte and McKee produced contained 34 causes.[8] Benign prostatic hyperplasia and thyroid diseases were two causes from Nolte and McKee's list that did not have corresponding causes in the GBD cause hierarchy. Thyroid diseases are captured in a residual causes group in the GBD cause hierarchy, and benign prostatic hyperplasia is not included because is not considered a cause of death in the GBD study. In addition, we disaggregated the category of "other infections" in Nolte and McKee to separately estimate diphtheria and tetanus. We did not include the two other infections from this group.

Section 2. Overview of modelling strategy for the Healthcare Access and Quality Index

Modelling overview

The flowchart below represents the overall modelling strategy we used to assemble the HAQ Index by age.

Appendix Figure 1. Reference information for cause of death methods

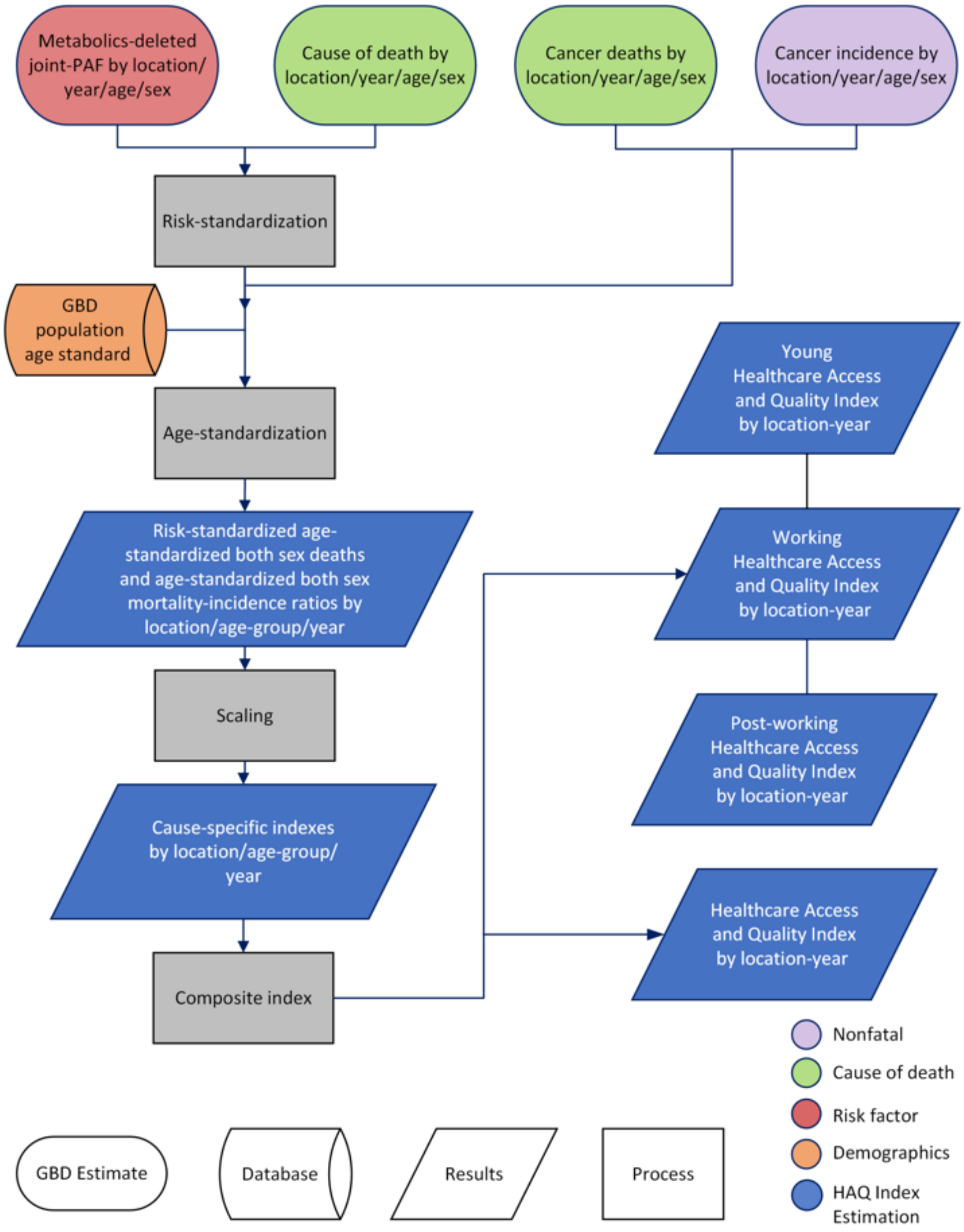

Inputs and modelling strategies

This analysis used results from the GBD 2019 research cycle. We used three broad categories of data to construct the index: cause-specific mortality rate estimates; comparative risk estimates; and mortality-

to-incidence ratios. Data were used from 910 locations, 23 age groups, two sexes, and 5 years spanning a 29-year range. These years were: 1990, 2000, 2005, 2010, 2019.

Detailed summaries of how causes were modelled in the GBD 2019 research cycle are available in Part 4 of this appendix.

Input data and estimates will be available for download from the Global Health Data Exchange (GHDx) online at: http://ghdx.healthdata.org/gbd-2019.Following methods from GBD 2016, joint population-attributable fractions (PAF) were estimated for each cause of death using comparative risk estimates from GBD 2019.[11] For each cause, all environmental and behavioral risks, excluding high blood pressure, high total cholesterol, and high fasting plasma glucose, were used to estimate the joint PAF. The three metabolic risks were excluded from standardization on the basis that they are measures that are amenable to personal healthcare. To determine how much one risk is mediated through another, published studies were used to estimate mediation factors for every two risk-factors for an outcome. The calculation of this resulted in a matrix of parameters that gave the aggregated burden by disease and for all risk factors. The formula for this computation is:

$$PAF_{J,o,a,s,l,t} = 1 - \prod_{j=1}^{J} (1 - PAF_{j,o,a,s,l,t} \prod_{i=1}^{J} (1 - MF_{j,i,o}))$$

where J is a set of risk factors for the aggregation; $PAF_{j,o,a,s,l,t}$ is the PAF for risk j for cause o, GBD age group a, sex s, location l, and year t; and $MF_{j,I,o}$ is the mediation factor for risk j mediated through i for cause o.

Risk-standardisation

Our standardisation process in this HAQ Index analysis followed the methodology used in the GBD 2016 HAQ Index study.[3] We wanted the HAQ Index to capture differences across locations and time in access to quality health-care, and not variation across locations in exposure to risk. Standardisation was completed by replacing local levels of exposure to risks with the global level of exposure for a given year. We used the following formula to risk-standardise:

$$RSD_{l,y,g,s} = D_{l,y,a,s} \times (1 - PAF_{l,y,a,s}) \times \frac{1}{1 - GPAF_{a,s}}$$

where $RSD_{l,y,a,s}$ is the risk-standardised deaths in location l, year y, HAQ age a, and sex s; $D_{l,y,g,s}$ are the deaths for the given specifications; $PAF_{l,y,a,s}$ is the PAF for the given specifications; and $GPAF_{a,s}$ is the global PAF for all years for age group a, and sex s.

For causes that either had no attributable risks, had a PAF of zero, or had a PAF of one, the observed death rate was used. Additionally, if any cause had a maximum observed PAF greater than 0·9 but less than 1 for a given age and sex, the PAFs across all locations and years were scaled downwards so that the maximum PAF was 0·9. This was done to recognize that while there are marginal differences

between 90 percent and 100 percent of a death being attributed to population risk factors, there still exists room for standardization especially for causes that high variability of PAFs across locations.

In the case of ischaemic heart disease, new data on the interplay of household air pollution, blood pressure, and ischaemic heart disease mortality resulted in implausible risk-standardised death rates for many low-SDI to low-middle SDI countries when we only accounted for joint exposures to metabolic risks considered amenable to health care. We thus included household and outdoor air pollution in risk standardisation and plan to further examine these risk mediation pathways in the future. We excluded three metabolic risk factors that are amenable to healthcare through proper diagnosis and treatment: high LDL, high fasting plasma glucose, and high systolic blood pressure (amenable, for example, due to diagnosis and treatment of diabetes and hypertension, respectively).

Mortality-incidence ratios

First implemented in HAQ 2016, MIRs are an additional measure for measuring access and quality in health systems. By looking at amenable deaths relative to total incidence, a stronger signal of health system performance is captured. The formula used to calculate MIRs is the following:

$$MIR_{l,y,g,s} = \frac{D_{l,y,g,s}}{I_{l,y,g,s}}$$

where $MIR_{l,y,g,s}$ is a mortality-incidence ratios in location *l*, year *y*, HAQ age group *a*, and sex *s*; $D_{l,y,a,s}$ are deaths in location *l,* year *y*, HAQ age group *g*, and sex *s*; and $I_{l,y,a,s}$ is incidence in location *l*, year *y*, HAQ age group *g*, and sex *s.*

Risk-standardised death vs mortality-incidence ratios

Due to improvements seen from the use of MIRs for cancers in the 2016 HAQ Index, MIR expansion was tested for all causes that were non-chronic and for which GBD measures incidence. When determining whether to use an RSD or an MIR, healthy average life expectancy (HALE) was set as the gold standard measure for better health. The correlation across years and locations for both HALE and an RSD and HALE and an MIR was calculated; the measure that had the highest correlation between the two was chosen. Appendix Table 2 indicates for which causes an MIR was used instead of an RSD. For ischemic heart disease, a RSD was used because incidence was only measured for myocardial infarction while deaths measured all deaths from ischemic heart disease.

Appendix Table 2: The correlation of MIRs and RSDs, respectively, with HALE, for causes tested

Cause	MIR Correlation with HALE	RSD Correlation with HALE
Tuberculosis*	-0.82	-0.78

Diarrheal diseases*	-0.77	-0.74
Lower respiratory infections	-0.70	-0.88
Upper respiratory infections	-0.55	-0.59
Diphtheria	-0.26	-0.42
Whooping cough*	-0.75	-0.67
Tetanus	-0.14	-0.41
Measles	0.09	-0.57
Maternal disorders	-0.59	-0.79
Neonatal disorders	--	-0.85
Breast cancer*	-0.93	-0.38
Cervical cancer*	-0.83	-0.76
Uterine cancer*	-0.92	-0.21
Colon and rectum cancer*	-0.91	0.14
Testicular cancer*	-0.92	0.01
Hodgkin lymphoma*	-0.85	-0.46

Leukemia*	-0.69	-0.03
Rheumatic heart disease	0.38	-0.57
Hypertensive heart disease	--	-0.64
Peptic ulcer disease	-0.57	-0.81
Appendicitis	-0.49	-0.82
Chronic kidney disease	-0.34	-0.47
Congenital heart anomalies	--	-0.61
Adverse effects of medical treatment*	-0.74	-0.65
Non-melanoma skin cancer (squamous-cell carcinoma)*	-0.57	-0.44

*Mortality incidence ratio used instead of risk-standardized death rate.

Age-group analyses

To complete the age-group analyses special considerations were needed to fit within the Nolte and McKee and GBD framework. Age groups of 0-14, 15-64, and 65+ were determined from demographic literature a priori and then constrained within the Nolte and McKee amenable age groups. These translated into the young (0-14), working (15-64), and post-working (65-74) age groups. Based on the Nolte and McKee amenable age groups, causes were then either included or excluded depending on the age groups[10]. For the young HAQ Index 26 causes were included. For the working age index 27 causes were included. And for the post-working index 22 causes were included. Additionally, due to GBD estimation limits, not all causes had estimates across the full age range of what Nolte and McKee considered amenable. An example of this is seen in maternal disorders for which Nolte and McKee specify amenability from age 0-74 while GBD produces estimates only between ages 10-50. In these scenarios, the estimates that were available were used. A full breakdown of this can be by age group in Appendix Table 3.

Appendix Table 3. Causes for which mortality is amenable to health care, mapped to GBD causes, and amenable age ranges

	Amenable age ranges (years)
**Communicable, maternal, neonatal, and nutritional diseases**	
Tuberculosis*	0-14, 15-64, 65-74
Diarrhoea, lower & upper respiratory, and vaccine-preventable diseases	
Diarrheal diseases*	0-14
Lower respiratory infections	0-14, 15-64, 65-74
Upper respiratory infections	0-14, 15-64, 65-74
Diphtheria	0-14, 15-64
Whooping cough*	0-14
Tetanus	0-14, 15-64, 65-74
Measles	0-14
Maternal disorders	0-14, 15-64
Neonatal disorders	0-14
**Non-communicable diseases**	
Neoplasms	
Colon and rectum cancer*	0-14, 15-64, 65-74
Non-melanoma skin cancer (squamous-cell carcinoma)*	15-64, 65-74
Breast cancer*	15-64, 65-74
Cervical cancer*	15-64, 65-74
Uterine cancer*	15-64
Testicular cancer*	0-14, 15-64, 65-74
Hodgkin lymphoma*	0-14, 15-64, 65-74
Leukemia*	0-14, 15-64
Cardiovascular diseases	
Rheumatic heart disease	0-14, 15-64, 65-74
Ischemic heart disease	15-64, 65-74
Stroke	0-14, 15-64, 65-74
Hypertensive heart disease	15-64, 65-74
Chronic respiratory diseases	0-14
Digestive diseases	
Peptic ulcer disease	0-14, 15-64, 65-74
Appendicitis	0-14, 15-64, 65-74
Inguinal, femoral, and abdominal hernia	0-14, 15-64, 65-74
Gallbladder and biliary diseases	0-14, 15-64, 65-74
Neurological disorders	
Idiopathic epilepsy	0-14, 15-64, 65-74
Diabetes, urogenital, blood, and endocrine diseases	
Diabetes mellitus	0-14, 15-64
Chronic kidney disease	0-14, 15-64, 65-74
Other non-communicable diseases	
Congenital heart anomalies	0-14, 15-64, 65-74
**Injuries**	
Unintentional injuries	
Adverse effects of medical treatment*	0-14, 15-64, 65-74

Although 0 (at birth) to 1 are listed as the lower bound of age ranges, age restrictions are applied for many causes such that mortality estimates are not produced before a given age group (eg, 15–19 years for many non-communicable diseases). Causes are ordered on the basis of the GBD cause list and corresponding group hierarchies.
GBD=Global Burden of Disease. *Mortality incidence ratio used instead of risk-standardized death rate.

Appendix Table 3. Causes for which mortality is amenable to health care, mapped to GBD causes, and amenable age ranges.

Age-standardisation

For both the composite and the age-group analyses, mortality and incidence were age standardised to control for differing age structures across locations. To standardize each cause, we compiled risk-standardised deaths for non-cancers and the initial components of cancer MIRs (i.e., mortality and incidence) for both sexes by location, year, age group, and amenable cause and computed the following:

$$RSASD_{l,y,g} = \sum_{a=1}^{n} RSD_{l,y,g,a} \times PAS_a$$

Where $RSASD_{l,y,g,d}$ is the age-standardised risk-standardised deaths or component parts for MIRs for location l, year y, and HAQ age group g, $RSD_{l,y,g,a}$ is the risk-standardised deaths or component parts for MIRs in location l, year y, HAQ age group g and GBD age group a; and PAS_a the population age standard for GBD age group a.

Although the highest age seen in the analyses was 74, GBD produces age weights from birth to 95+. To reconcile the differences in age groups, cause-specific age weights were recalculated by summing the age weights within each age group to be rescaled to 1 within each age group (young, working and post-working, as well as the composite).

Scaling Causes

Age-standardised MIRs and age-standardised RSDs were further standardized by scaling them from 0 to 100 using the following formula:

$$SI_{l,y,g} = \frac{\log(I_{c,l,y,g}) - 1st(\log(I'_{c,g}))}{99th(\log(I'_{c,g})) - 1st(\log(I'_{c,g}))}$$

Where $SI_{l,y,d}$ is equal to the scaled indicator for each MIR or RSD and $I_{c,l,y,d}$ represents the age-standardised RSD or MIR for a cause *c,* location *l*, year *y*, and HAQ age group *g* prior to scaling. $I'_{c,d}$ represents the 1st and 99th percentiles of draws that set the minimum and maximum value for each cause across locations and years. A log-offset of 10^{-6} was added to all unscaled indicators prior to being scaled in log space in order to eliminate zeroes in a small number of age-cause combinations.

The scaling of each cause is done independently by age group. The scale of a given cause is not the same for the young age group as the scales for the working, post-working or composite groups, respectively. Thus, scaling of a given cause is comparable across countries within an age group but not across age groups.

Creating the HAQ Index

The final HAQ Index for the composite and each age group is comprised of the mean of the scaled causes, with the scaled causes computed for each HAQ Index group independently. For the composite index, 32 causes were used, while for the young, working, and post-working age group indicators, 26, 27, and 22 causes, respectively, were used. Because each HAQ Index is based on independently scaled causes, the values of each of the four HAQ Indices are not comparable except with respect to

representing the distance for each cause to the maximum/minimum values and the scale between the maximum and minimum values across each HAQ Index group, as discussed in “Scaling Causes” section.

Taking the arithmetic mean of the scaled causes to compute each HAQ Index differed from the two previous iterations of HAQ, which constructed the index based on cause-weights derived from the relative contribution to a composite PCA factor. In this iteration of the HAQ Index, the arithmetic mean was used in order to improve the external interpretability of the HAQ Index while maintaining the cross-country patterns of the PCA-weighted version of the index. The mean weighted HAQ Index and the PCA-weighted HAQ Index were very similar, with a very strong correlation as shown in Figures 2a-2c which plot the HAQ Index based on the mean versus the HAQ Index based on PCA weights and depict the correlation between the two. Additionally, we elected to use the arithmetic mean over the geometric mean. The arithmetic and geometric means weighted HAQ indices were also very similar, with a strong correlation as show in figures 3a-3d.

Appendix Figure 2a. HAQ PCA Weighted Overall Index (0-74) vs Mean Overall Index (0-74), 2017

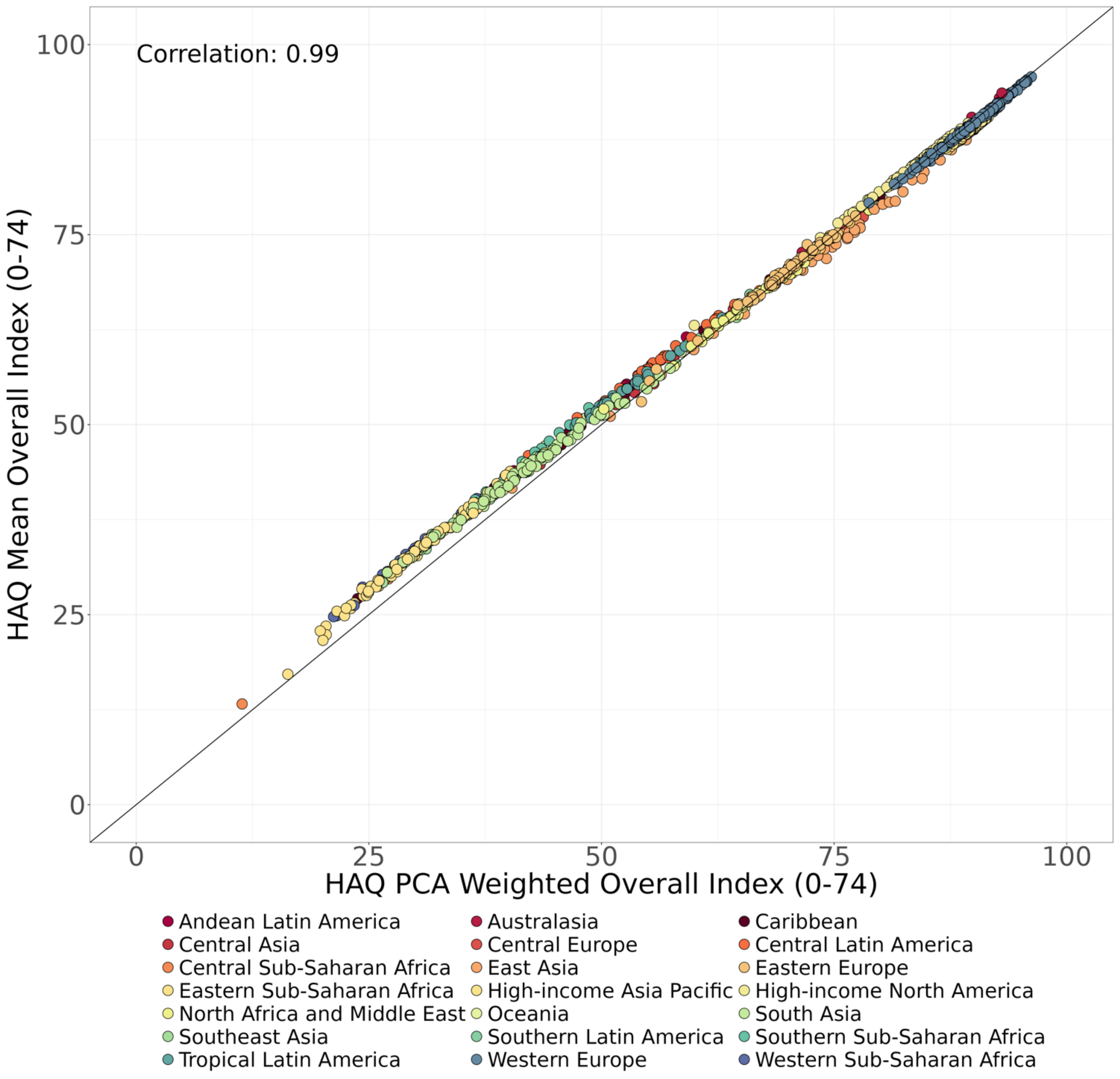

Appendix Figure 2b. HAQ PCA Weighted Young Index (0-14) vs Mean Young Index (0-14), 2017

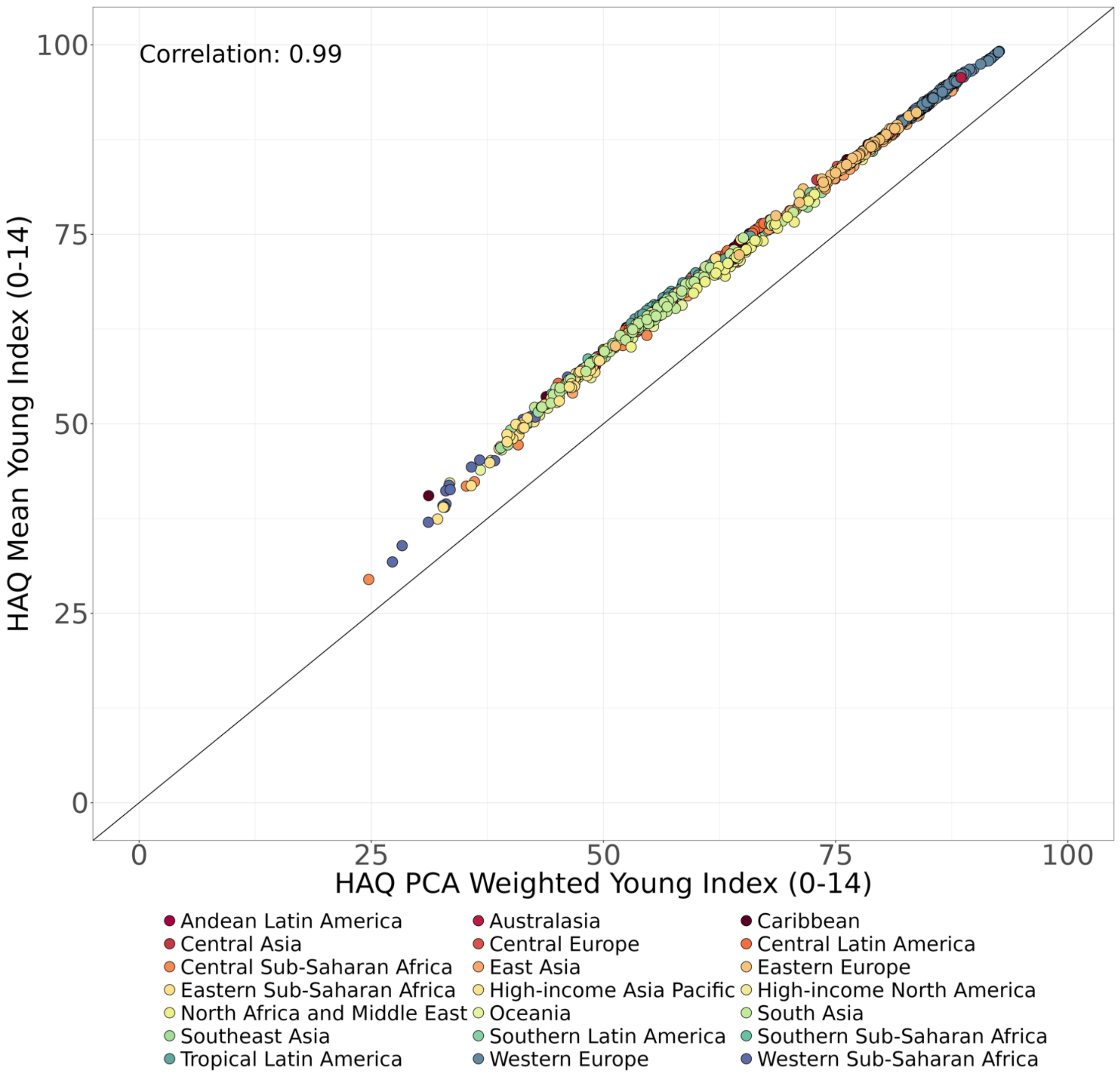

Appendix Figure 2c. HAQ PCA Weighted Working Index (15-64) vs Mean Working Index (15-64), 2017

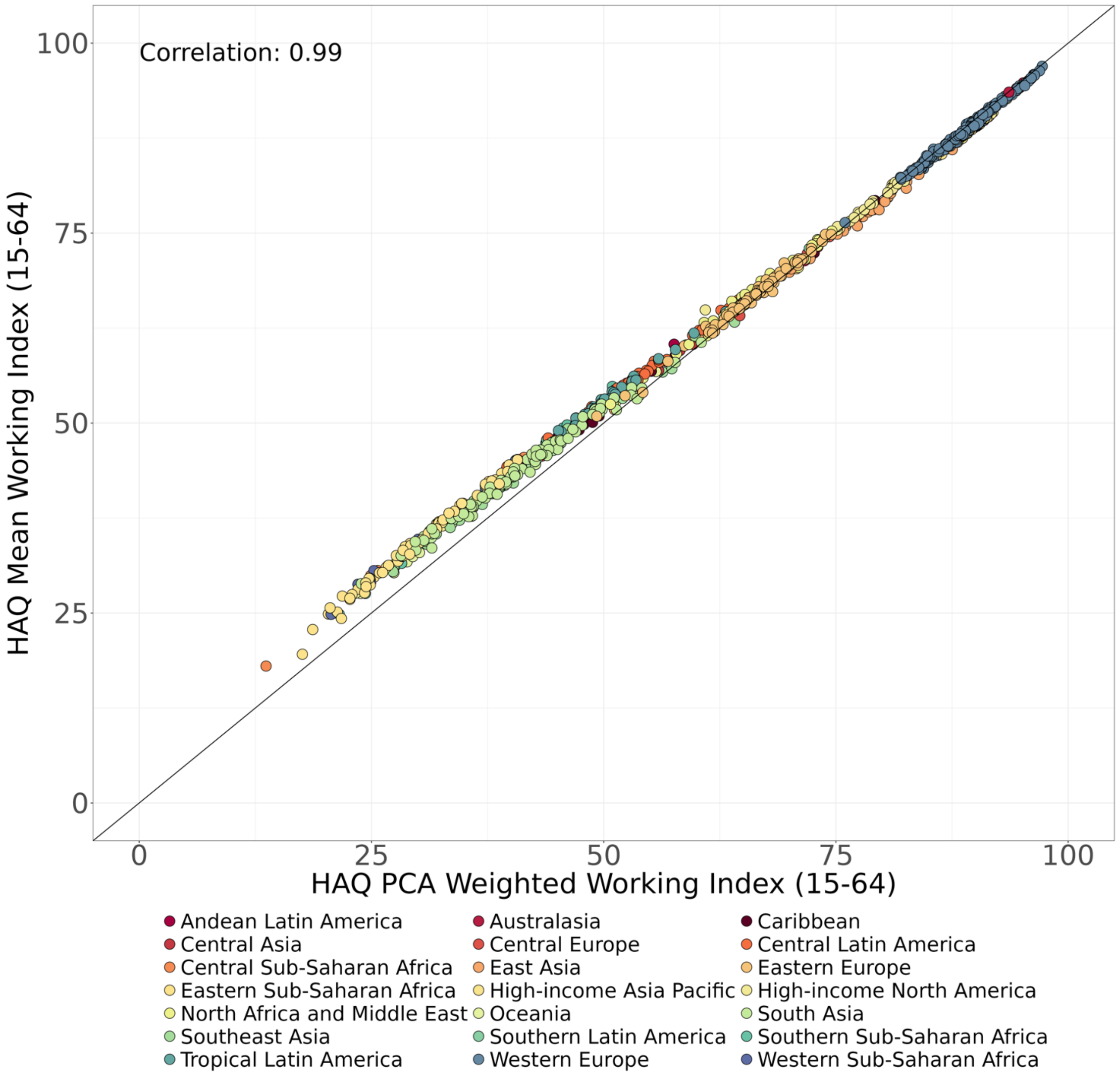

Appendix Figure 2d. HAQ PCA Weighted Post-working Index (65-74) vs Mean Post-working Index (65-74), 2017

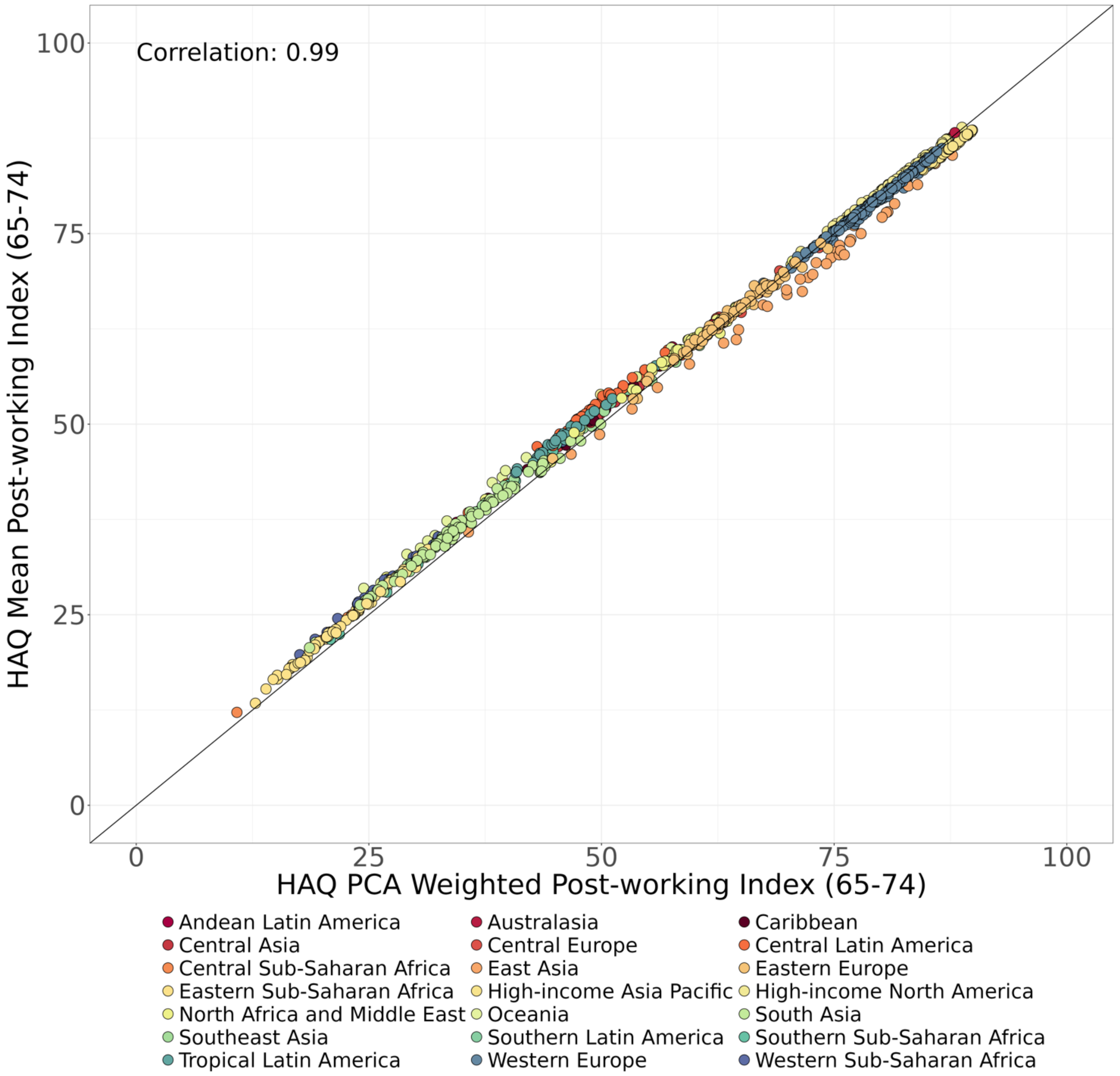

Appendix Figure 3a. HAQ Geometric Mean Overall Index (0-74) vs Arithmetic Mean Overall Index (0-74), 2019

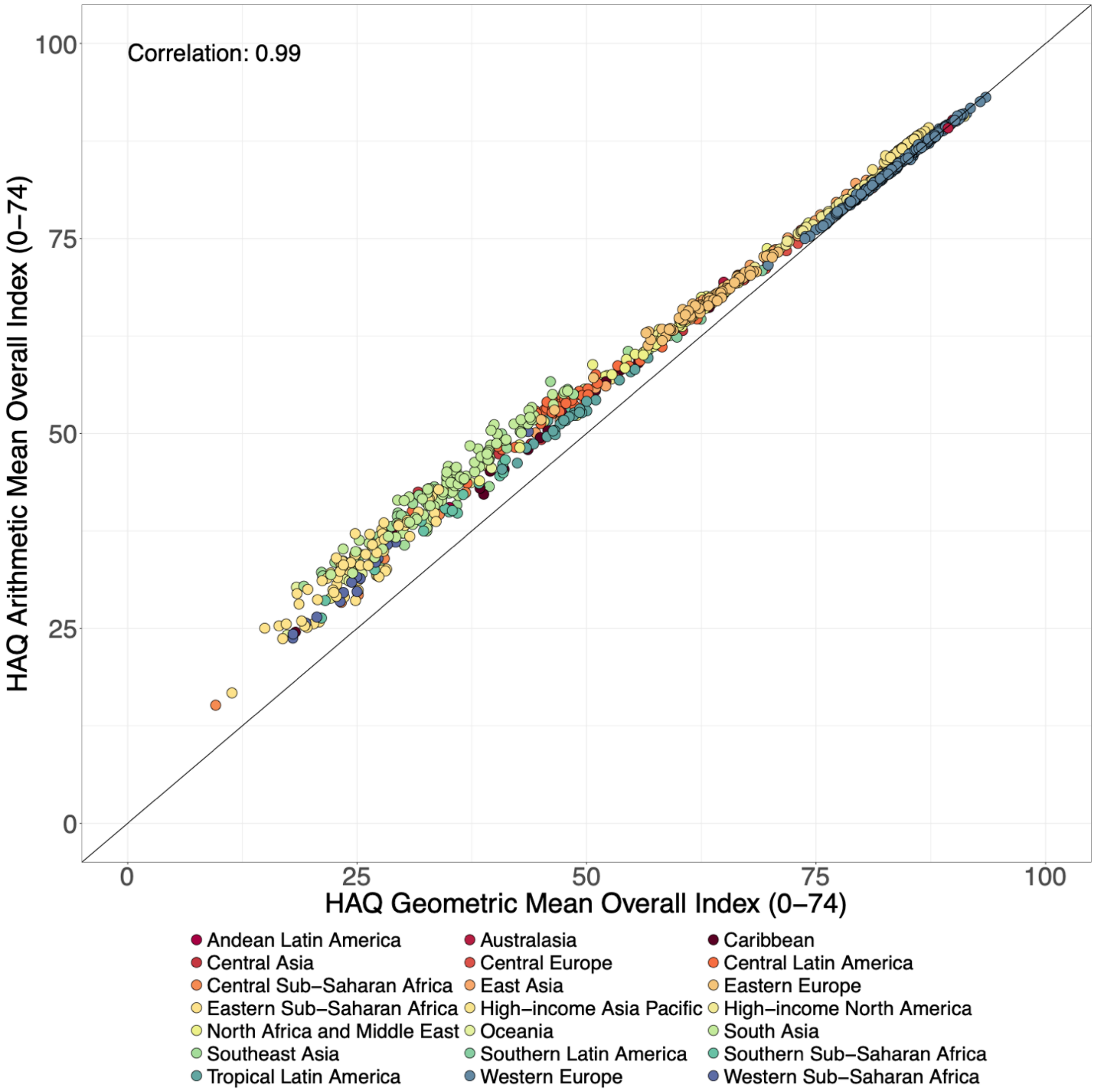

Appendix Figure 3b. HAQ Geometric Mean Young Index (0-14) vs Arithmetic Mean Young Index (0-14), 2019

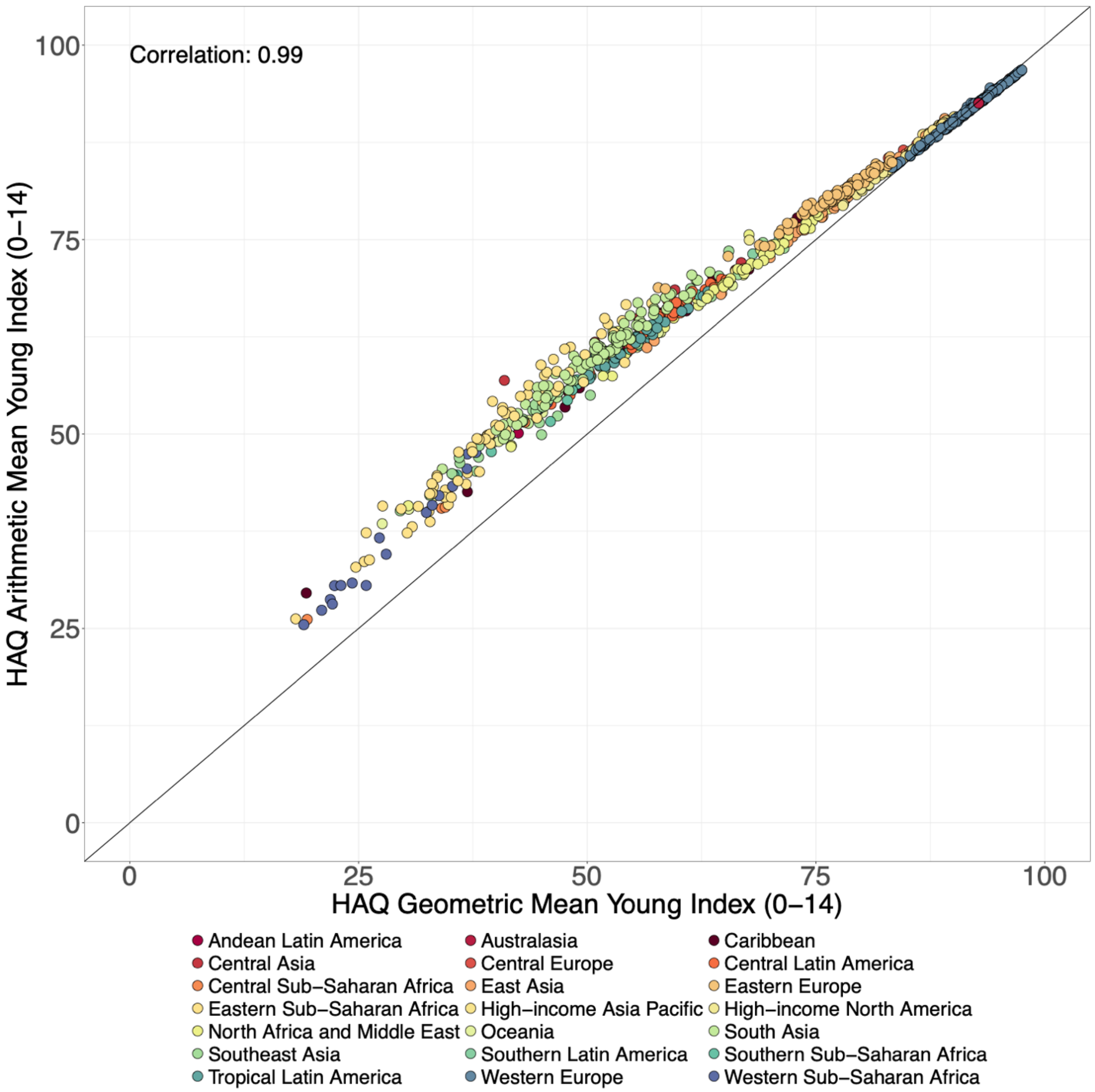

Appendix Figure 3c. HAQ Geometric Mean Working Index (15-64) vs Arithmetic Mean Working Index (15-64), 2019

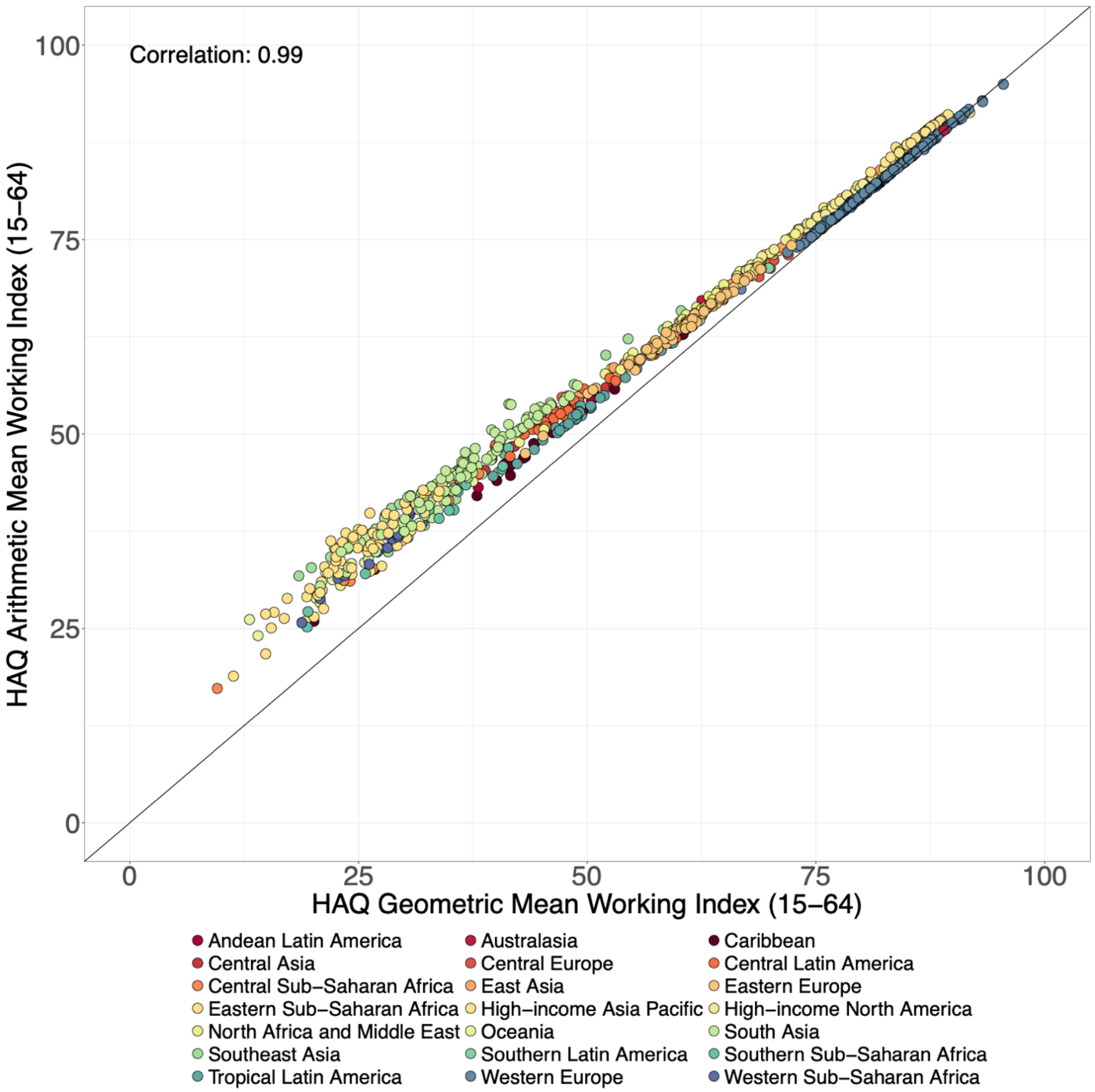

Appendix Figure 3d. HAQ Geometric Mean Post-working Index (65-74) vs Arithmetic Mean Working Index (65-74), 2019

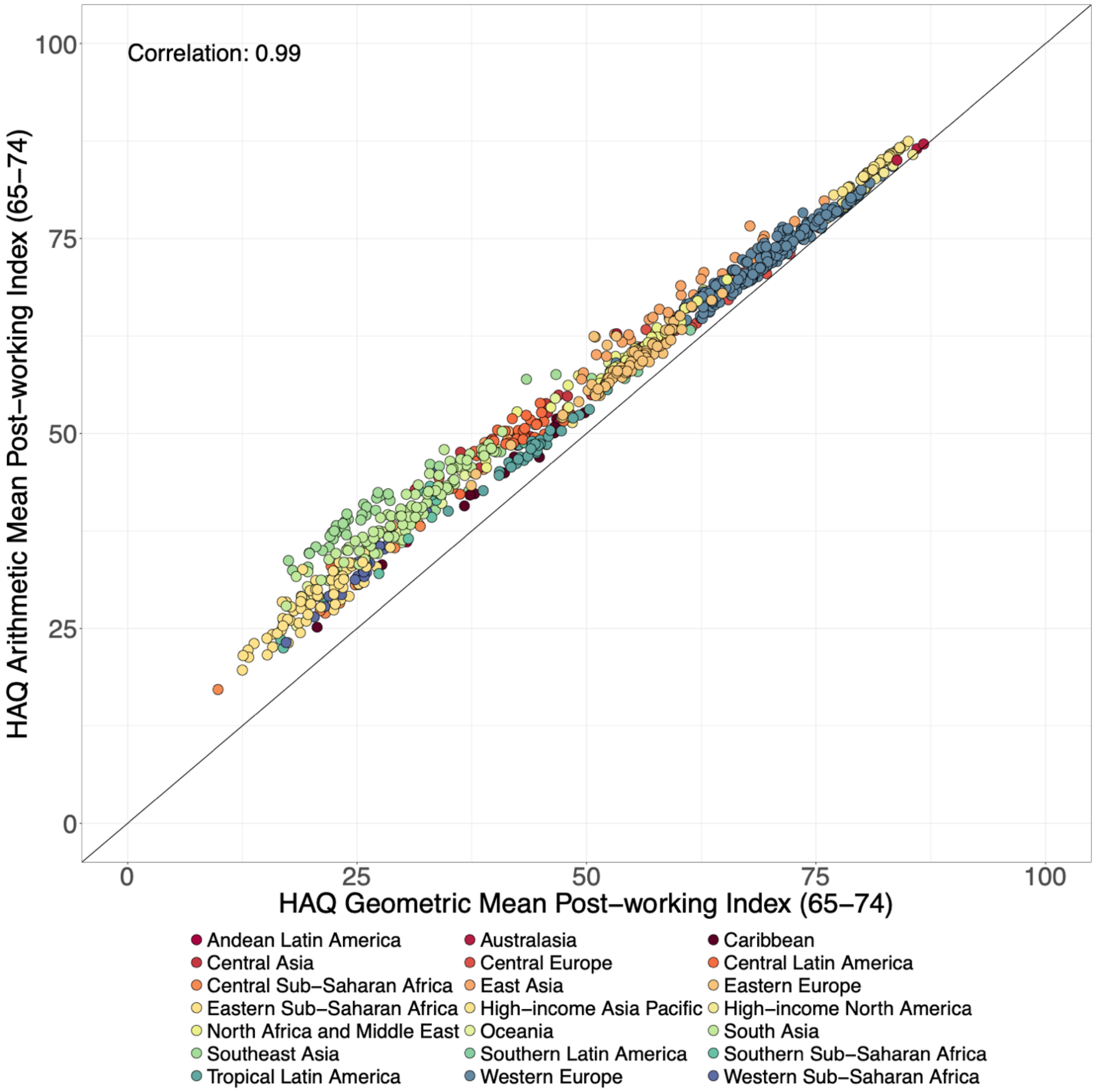

Uncertainty analysis

We created 1000 draws for each input into the HAQ Index, where an input is a location-year for each of the 32 causes for the composite index, 26 causes for the young HAQ Index, 27 causes for the working HAQ Index and 22 causes for the post-working HAQ Index. For each draw, the mean of all causes was computed to calculate the HAQ and the uncertainty intervals are represented by the 2.5 and 97.5 percentiles of the 1000 calculated HAQ Index draws, computed separately for each age group and the composite score.

Part 2. Estimating convergence

For each age group, we examine the convergence with the top country performers on the HAQ Index. We consider convergence as representing health system performance worldwide becoming closer to the best observed performance across countries, examined separately by age group. We consider three forms of convergence.

Beta convergence

First, we examine "beta" convergence or whether the countries with the lowest scores caught up with, or increased faster than, increases in the top scores. We do this by regressing the 2019 HAQ Index on the absolute change between 1990 and 2019 in the HAQ Index score. Appendix Table 4a presents the results of these from these ordinary least squares regressions, representing absolute convergence. These results are discussed in the main text. Absolute convergence is observed only in the overall HAQ Index and the young HAQ Index (Appendix Table 4a). Appendix Table 4b captures relative convergence, depicting a regression of annualized percent change in the HAQ Index between 1990 and 2019 on the 1990 HAQ Index. Relative convergence is observed in all groups (Appendix Table 4b). For smaller 1990 values, which tend to be in low SDI countries, the same absolute change represents larger annualized percent change – results are subject to the "base rate" effect. Because of this effect and the fact that the limits of the index (0, 100) can change each time we update the HAQ Index – there are not so-called natural bounds or limits to the HAQ Index – we focus on absolute convergence rather than relative convergence in our main analysis. The results from Appendix Table 4b are also depicted in Appendix Figure 5.

Appendix Table 4a: Absolute change in HAQ Index, 1990 to 2019, by select age group

Term	Estimate	Std. Error	P value
Overall 1990 HAQ Index	0.060	0.023	0.01
Young 1990 HAQ Index	-0.132	0.022	< .0001
Working 1990 HAQ Index	0.113	0.026	< .0001
Post-working 1990 HAQ Index	0.207	0.031	< .0001

Appendix Table 4b: Annualized percent change in HAQ Index, 1990 to 2019, by select age group

Term	Estimate	Std. Error	P value
Overall 1990 HAQ Index	-0.022	0.002	< .0001
Young 1990 HAQ Index	-0.030	0.002	< .0001
Working 1990 HAQ Index	-0.013	0.002	< .0001
Post-working 1990 HAQ Index	-0.006	0.002	0.01

Appendix Figure 4: Absolute change in HAQ Index, 1990-2019, versus 1990 HAQ Index by select age group

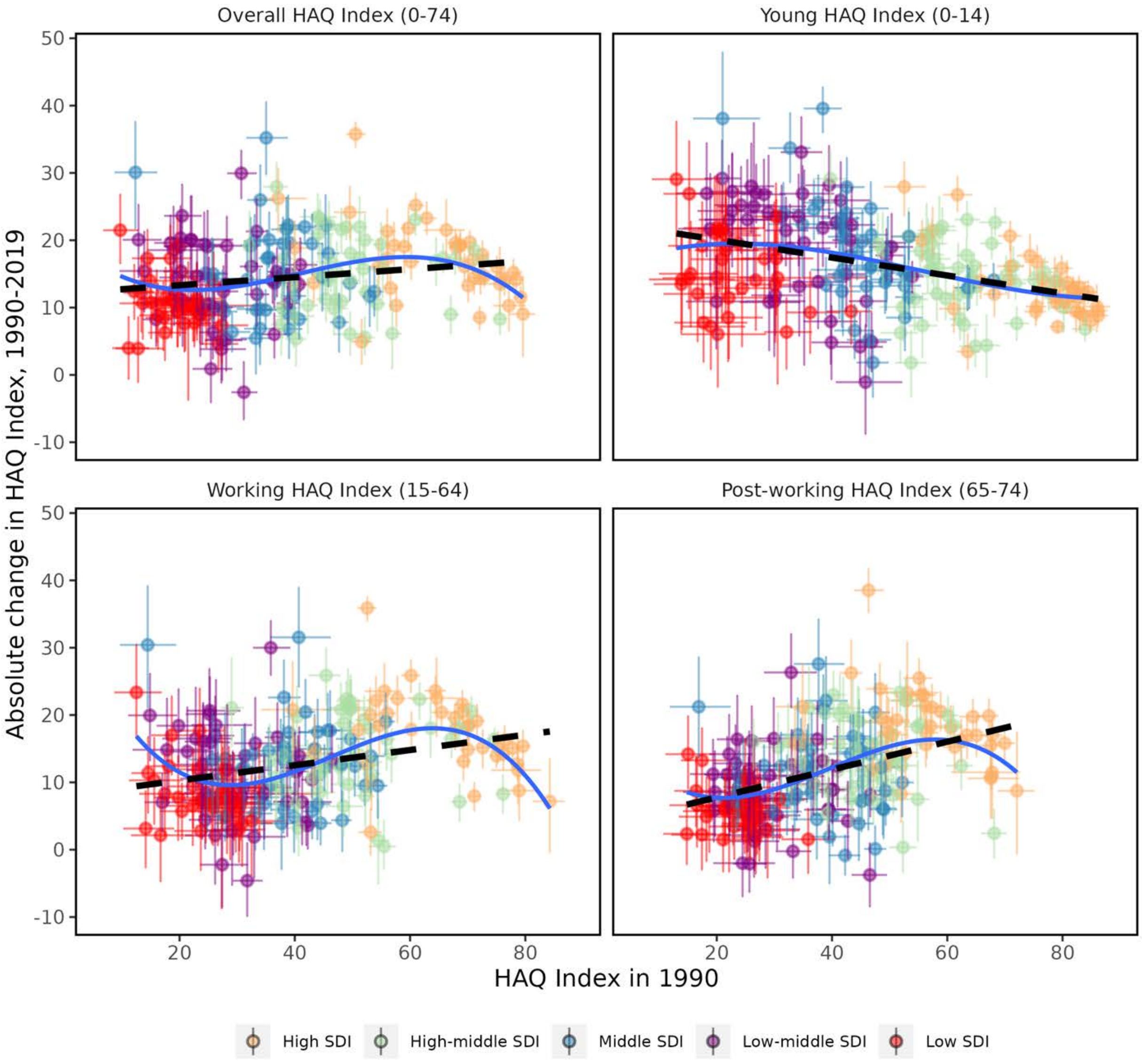

Appendix Figure 5. Annualized percent change in HAQ Index, 1990-2019, versus 1990 HAQ Index by select age group

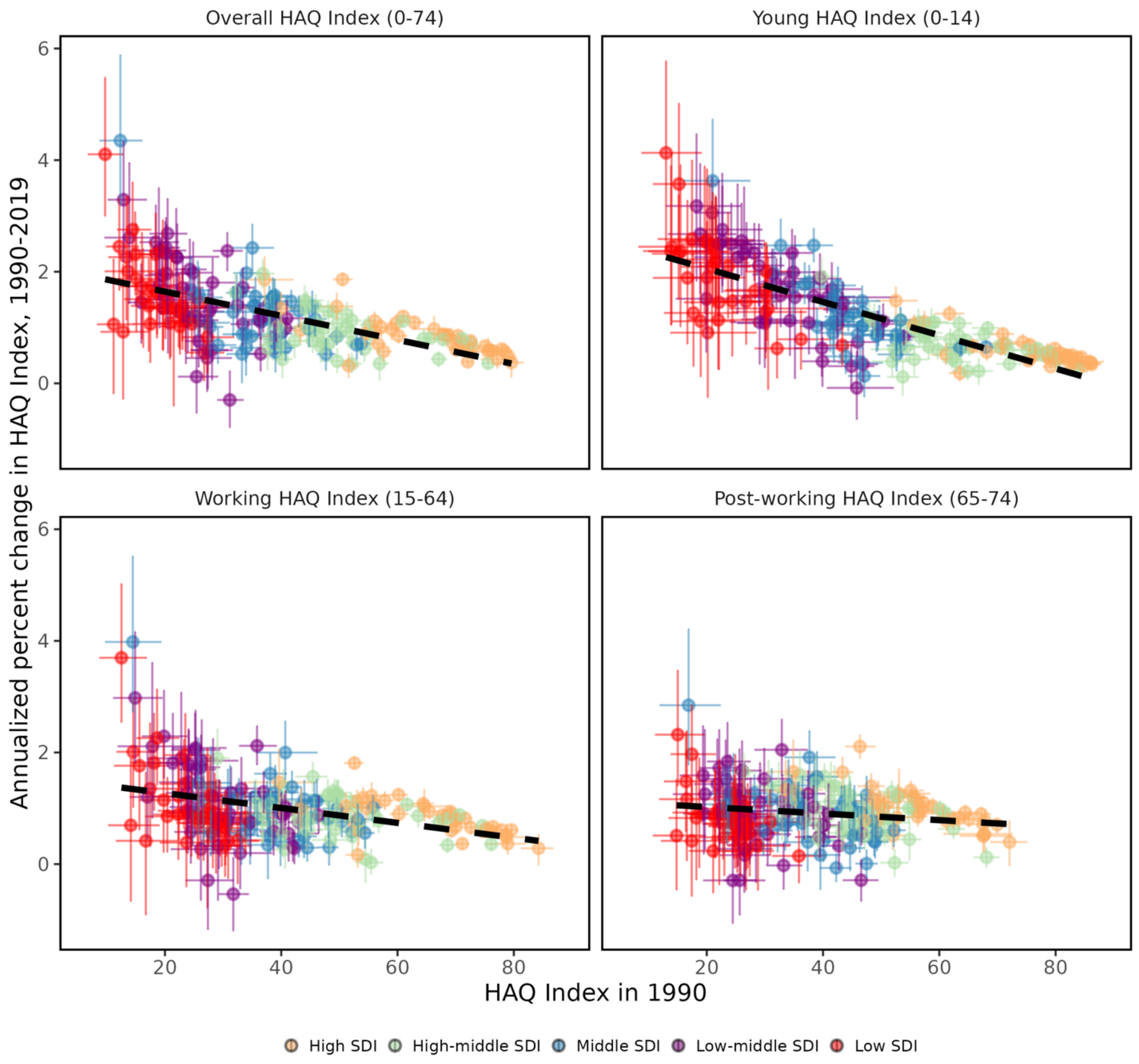

Sigma convergence

Second, we considered "sigma" convergence, which is whether scores become more similar overall. This is represented by Appendix Table 5, which depicts the standardized normal deviation (the standard deviation divided by the mean) for each of the three age groups in 1990 and 2019.

Appendix Table 5: Normalized Standard Deviation HAQ, 1990 and 2019

Age Group	Normalized Standard Deviation, 1990	Normalized Standard Deviation, 2019
Young	0.418	0.289
Working	0.397	0.358
Post-working	0.359	0.345

Convergence with High SDI quintile countries

Third, we examined convergence as defined by the average distance from the contemporaneous average HAQ Index in High SDI quintile countries, taken collectively as a representation of the best performance in the HAQ Index for each year. This is represented in Figures 6a-6d, which show the average distance to the High SDI country average for each SDI quintile and the overall and select age group HAQ Index scores.

Appendix Figure 6a. Trends in average gap with mean HAQ Index in the High SDI Quintile by select age group, Low SDI quintile, 1990-2019

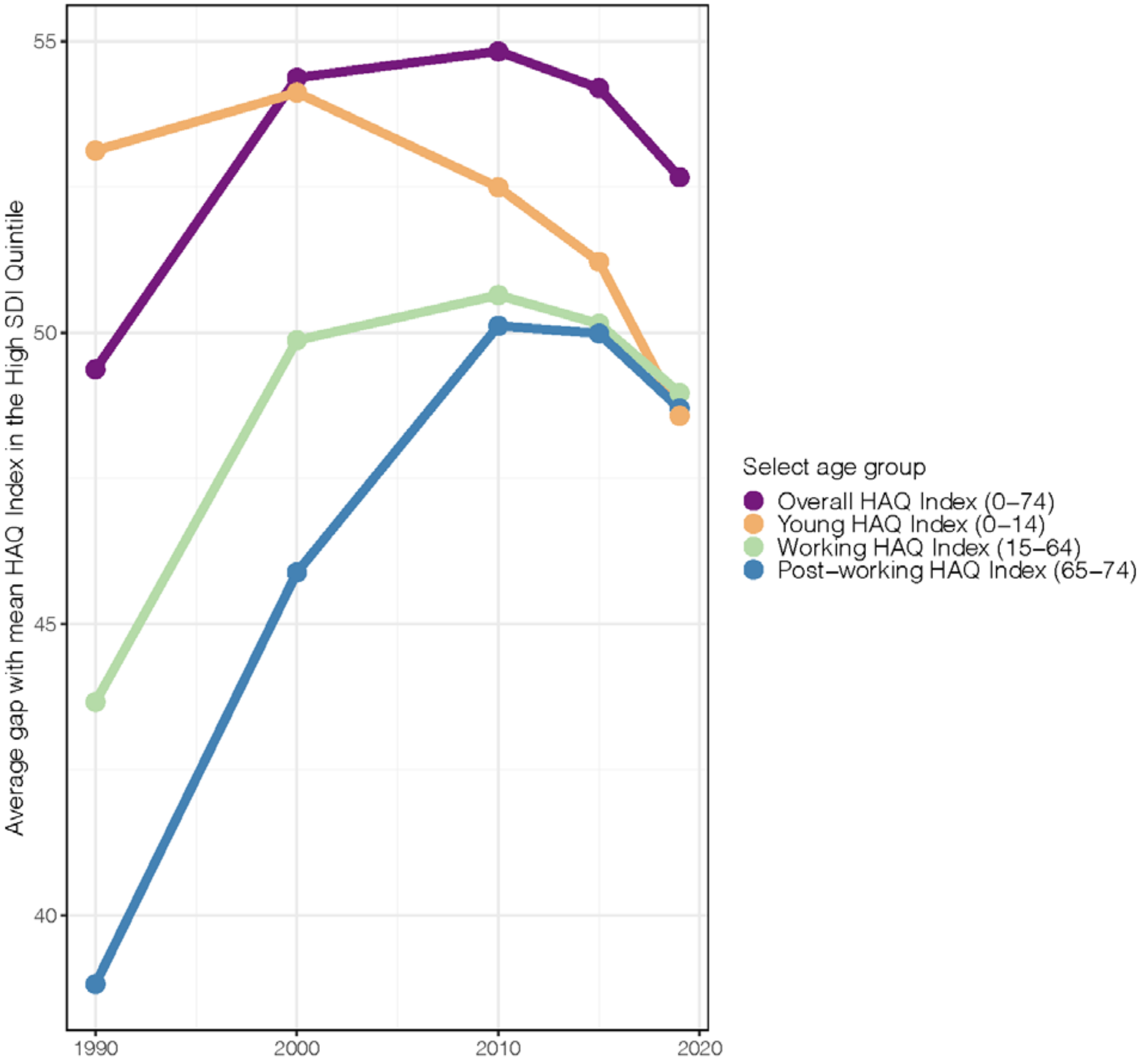

Appendix Figure 6b. Trends in average gap with mean HAQ Index in the High SDI Quintile by select age group, Low-middle SDI quintile, 1990-2019

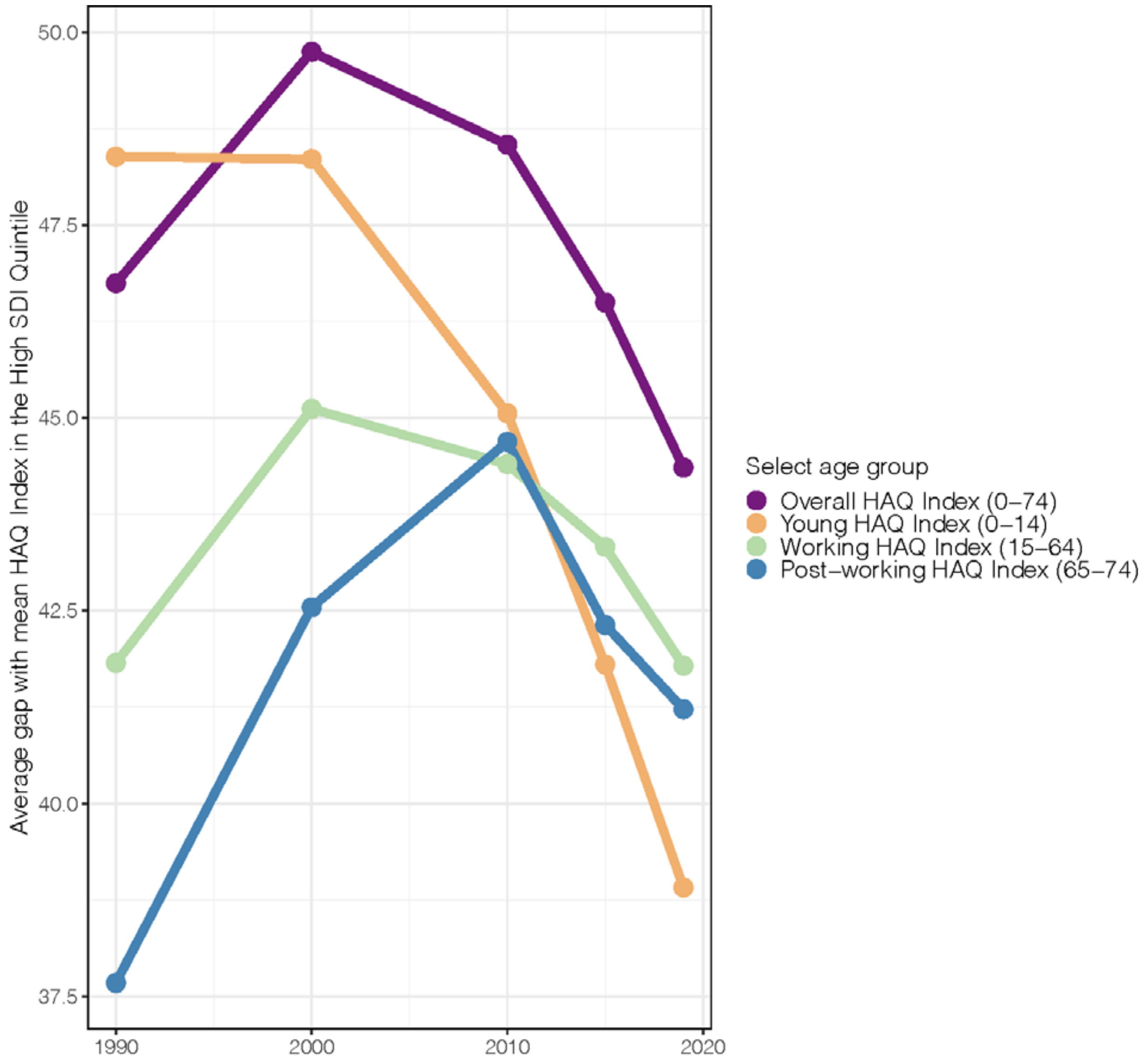

Appendix Figure 6c. Trends in average gap with mean HAQ Index in the High SDI Quintile by select age group, Middle SDI quintile, 1990-2019

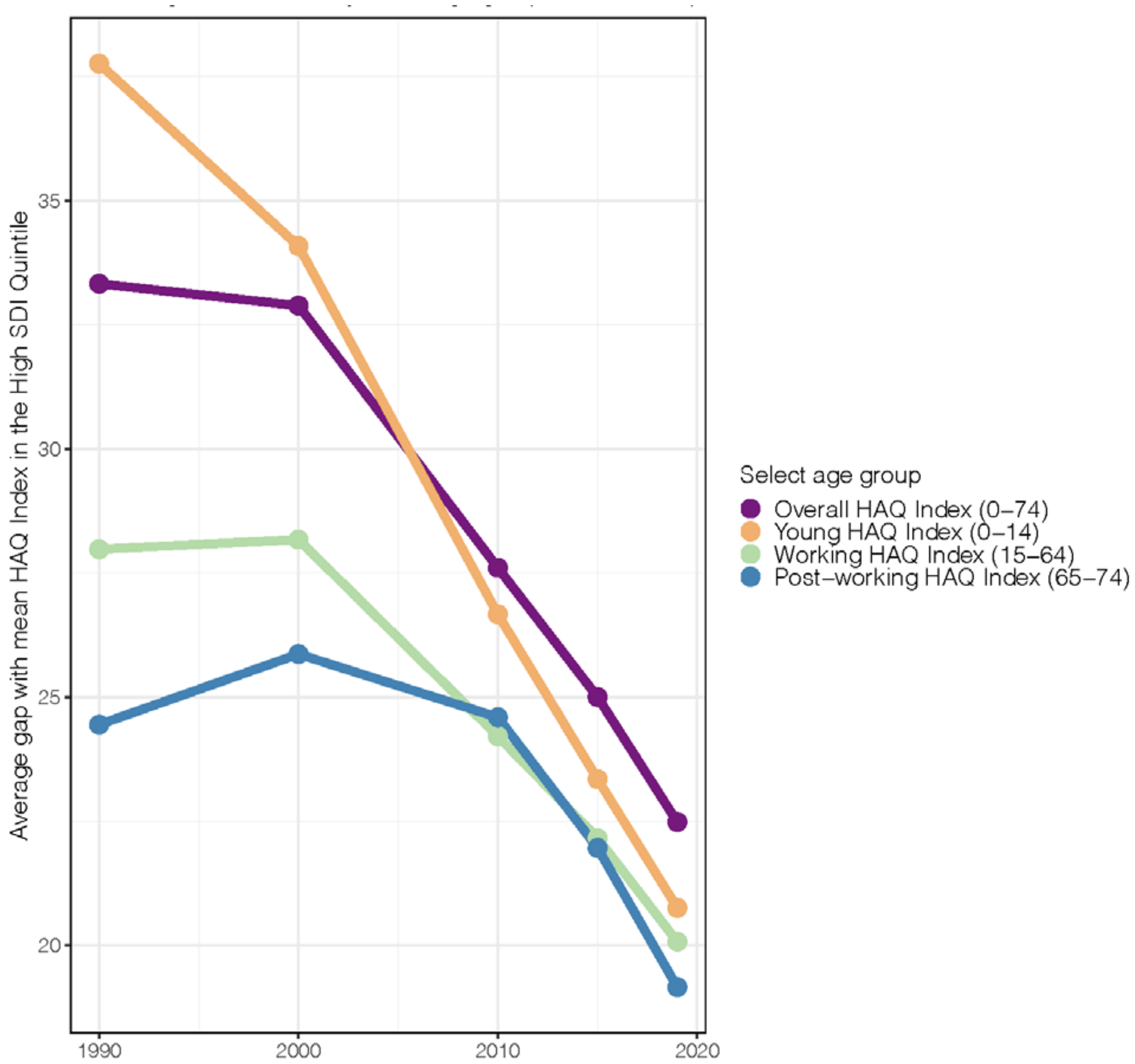

Appendix Figure 6d. Trends in average gap with mean HAQ Index in the High SDI Quintile by select age group, High-middle SDI quintile, 1990-2019

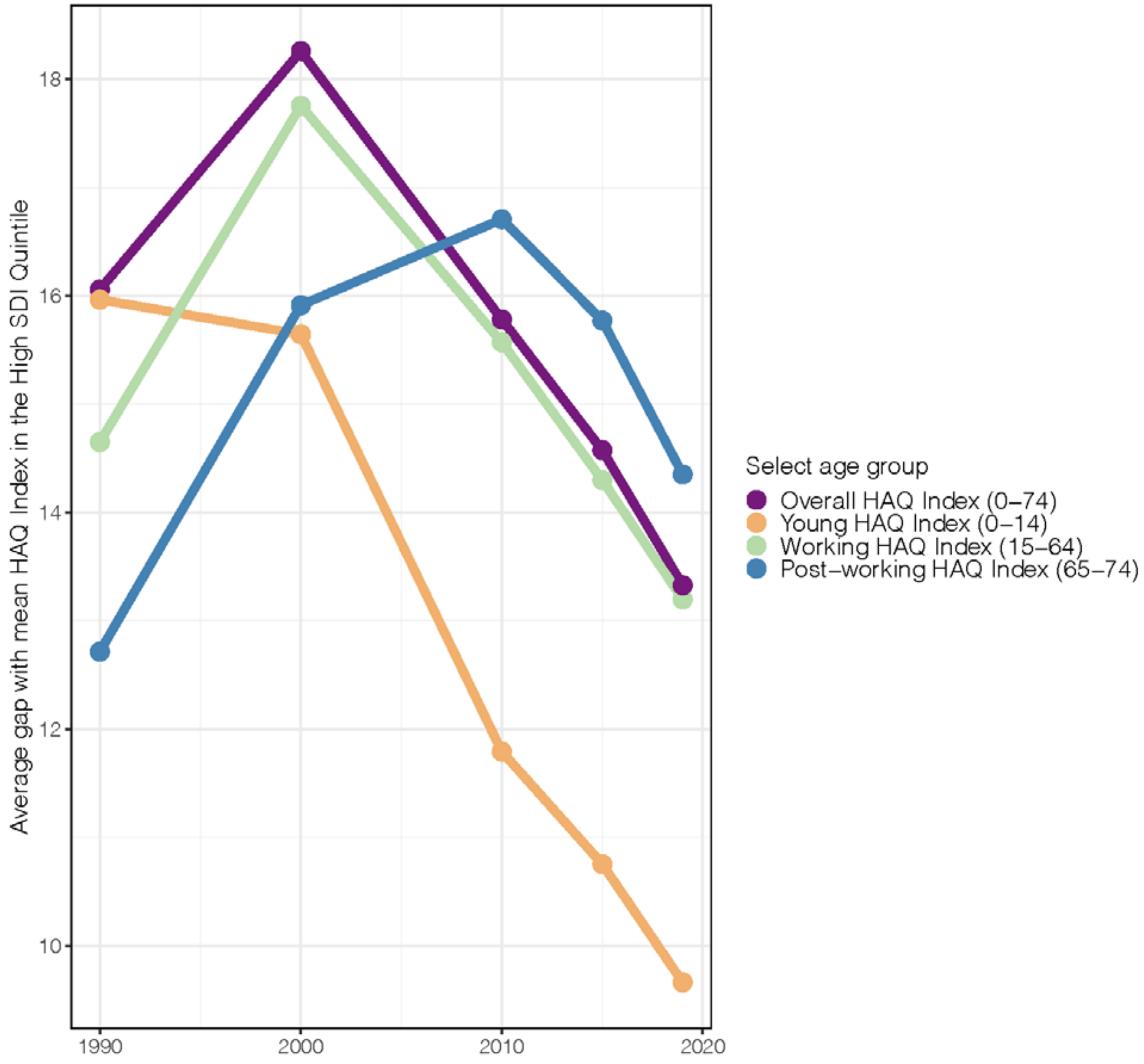

Appendix Table 6: HAQ Index regressed on SDI over 1990-2019 by select age group

Select age group	Estimate	Std. Error	P value	R^2
Overall	96.23	3.036	<0.001	0.83
Young	101.41	2.262	<0.001	0.89
Working	87.76	3.371	<0.001	0.75
Post-working	77.10	2.718	<0.001	0.77

Part 3. Online tools and glossary of terms

Section 1. Online tools

Data for all underlying causes used in the HAQ Index can be located and downloaded here: http://ghdx.healthdata.org/gbd-2019.

Section 2. List of abbreviations

GATHER – Guidelines for Accurate and Transparent Health Estimates Reporting
GBD – Global Burden of Diseases, Injuries, and Risk Factors Study
GHDx – Global Health Data Exchange
HAQ Index – Healthcare Access and Quality Index
MIRs – Mortality-to-incidence ratios
PCA – Principal Component Analysis
SDI – Socio-demographic Index
UI – Uncertainty Interval

Section 3. List of ISO3 codes and location names

ABW Aruba

AFG Afghanistan

AGO Angola

AIA Anguilla

ALA Åland Islands

ALB Albania

AND Andorra

ANT Netherlands Antilles

ARE United Arab Emirates

ARG Argentina

ARM Armenia

ASM American Samoa

ATA Antarctica

ATF French Southern Territories

ATG Antigua and Barbuda

AUS Australia

AUT Austria

AZE Azerbaijan

BDI Burundi

BEL Belgium

BEN Benin

BFA Burkina Faso

BGD Bangladesh

BGR Bulgaria

BHR Bahrain

BHS Bahamas

BIH Bosnia and Herzegovina

BLM Saint Barthélemy

BLR Belarus

BLZ Belize

BMU Bermuda

BOL Bolivia, Plurinational State of

BRA Brazil

BRB Barbados

BRN Brunei Darussalam

BTN Bhutan

BVT Bouvet Island

BWA Botswana

CAF Central African Republic

CAN Canada

CCK Cocos (Keeling) Islands

CHE Switzerland

CHL Chile

CHN China

CIV Côte d'Ivoire

CMR Cameroon

COD Congo, the Democratic Republic of the

COG Congo

COK Cook Islands

COL Colombia

COM Comoros

CPV Cape Verde

CRI Costa Rica

CUB Cuba

CXR Christmas Island

CYM Cayman Islands

CYP Cyprus

CZE Czech Republic

DEU Germany

DJI Djibouti

DMA Dominica

DNK Denmark

DOM Dominican Republic

DZA Algeria

ECU Ecuador

EGY Egypt

ERI Eritrea

ESH Western Sahara

ESP Spain

EST Estonia

ETH Ethiopia

FIN Finland

FJI Fiji

FLK Falkland Islands (Malvinas)

FRA France

FRO Faroe Islands

FSM Micronesia, Federated States of

GAB Gabon

GBR United Kingdom

GEO Georgia

GGY Guernsey

GHA Ghana

GIB Gibraltar

GIN Guinea

GLP Guadeloupe

GMB Gambia

GNB Guinea-Bissau

GNQ Equatorial Guinea

GRC Greece

GRD Grenada

GRL Greenland

GTM Guatemala

GUF French Guiana

GUM Guam

GUY Guyana

HKG Hong Kong

HMD Heard Island and McDonald Islands

HND Honduras

HRV Croatia

HTI Haiti

HUN Hungary

IDN Indonesia

IMN Isle of Man

IND India

IOT British Indian Ocean Territory

IRL Ireland

IRN Iran, Islamic Republic of

IRQ Iraq

ISL Iceland

ISR Israel

ITA Italy

JAM Jamaica

JEY Jersey

JOR Jordan

JPN Japan

KAZ Kazakhstan

KEN Kenya

KGZ Kyrgyzstan

KHM Cambodia

KIR Kiribati

KNA Saint Kitts and Nevis

KOR Korea, Republic of

KWT Kuwait

LAO People's Democratic Republic

LBN Lebanon

LBR Liberia

LBY Libyan Arab Jamahiriya

LCA Saint Lucia

LIE Liechtenstein

LKA Sri Lanka

LSO Lesotho

LTU Lithuania

LUX Luxembourg

LVA Latvia

MAC Macao

MAF Saint Martin (French part)

MAR Morocco

MCO Monaco

MDA Moldova, Republic of

MDG Madagascar

MDV Maldives

MEX Mexico

MHL Marshall Islands

MKD Macedonia, the former Yugoslav Republic of

MLI Mali

MLT Malta

MMR Myanmar

MNE Montenegro

MNG Mongolia

MNP Northern Mariana Islands

MOZ Mozambique

MRT Mauritania

MSR Montserrat

MTQ Martinique

MUS Mauritius

MWI Malawi

MYS Malaysia

MYT Mayotte

NAM Namibia

NCL New Caledonia

NER Niger

NFK Norfolk Island

NGA Nigeria

NIC Nicaragua

NIU Niue

NLD Netherlands

NOR Norway

NPL Nepal

NRU Nauru

NZL New Zealand

OMN Oman

PAK Pakistan

PAN Panama

PCN Pitcairn

PER Peru

PHL Philippines

PLW Palau

PNG Papua New Guinea

POL Poland

PRI Puerto Rico

PRK Korea, Democratic People's Republic of

PRT Portugal

PRY Paraguay

PSE Palestinian Territory, Occupied

PYF French Polynesia

QAT Qatar

REU Réunion

ROU Romania

RUS Russian Federation

RWA Rwanda

SAU Saudi Arabia

SDN Sudan

SEN Senegal

SGP Singapore

SGS South Georgia and the South Sandwich Islands

SHN Saint Helena, Ascension and Tristan da Cunha

SJM Svalbard and Jan Mayen

SLB Solomon Islands

SLE Sierra Leone

SLV El Salvador

SMR San Marino

SOM Somalia

SPM Saint Pierre and Miquelon

SRB Serbia

STP Sao Tome and Principe

SUR Suriname

SVK Slovakia

SVN Slovenia

SWE Sweden

SWZ Swaziland

SYC Seychelles

SYR Syrian Arab Republic

TCA Turks and Caicos Islands

TCD Chad

TGO Togo

THA Thailand

TJK Tajikistan

TKL Tokelau

TKM Turkmenistan

TLS Timor-Leste

TON Tonga

TTO Trinidad and Tobago

TUN Tunisia

TUR Turkey

TUV Tuvalu

TWN Taiwan, Province of China

TZA Tanzania, United Republic of

UGA Uganda

UKR Ukraine

UMI United States Minor Outlying Islands

URY Uruguay

USA United States

UZB Uzbekistan

VAT Holy See (Vatican City State)

VCT Saint Vincent and the Grenadines

VEN Venezuela, Bolivarian Republic of

VGB Virgin Islands, British

VIR Virgin Islands, U.S.

VNM Viet Nam

VUT Vanuatu

WLF Wallis and Futuna

WSM Samoa

YEM Yemen

ZAF South Africa

ZMB Zambia

ZWE Zimbabwe

Section 4. SDI reference quintiles and country-specific SDI values

Appendix Table 7. SDI reference quintiles and country-specific SDI values

SDI quintile	Location	SDI Estimate
High SDI (0.81-1.00)		
	Switzerland	0.93
	Norway	0.91
	Monaco	0.90
	Germany	0.90
	Luxembourg	0.89
	Andorra	0.89
	Denmark	0.89
	San Marino	0.88
	Netherlands	0.88
	United Arab Emirates	0.88
	Republic of Korea	0.88
	Canada	0.87
	Sweden	0.87
	Japan	0.87
	Iceland	0.87
	Taiwan (Province of China)	0.87
	Ireland	0.87
	Singapore	0.86
	United States of America	0.86
	Finland	0.86

	Kuwait	0.85
	Belgium	0.85
	Austria	0.85
	United Kingdom	0.85
	Lithuania	0.84
	Cyprus	0.84
	Slovenia	0.84
	New Zealand	0.84
	Australia	0.84
	Estonia	0.84
	France	0.83
	Qatar	0.83
	Czechia	0.83
	Brunei Darussalam	0.82
	Latvia	0.82
	Puerto Rico	0.81
	Bermuda	0.81
	Guam	0.81
	Slovakia	0.81
High-middle SDI (0.69-0.81)		
	Saudi Arabia	0.81
	Russian Federation	0.81
	Israel	0.80
	Poland	0.80
	Malta	0.80
	Italy	0.80
	United States Virgin Islands	0.80

	Bahamas	0.80
	Croatia	0.79
	Greece	0.79
	Hungary	0.79
	Montenegro	0.79
	Oman	0.78
	Northern Mariana Islands	0.77
	Spain	0.77
	Serbia	0.77
	Cook Islands	0.76
	Bulgaria	0.76
	Greenland	0.76
	Romania	0.76
	Chile	0.76
	Trinidad and Tobago	0.76
	Bahrain	0.75
	Turkey	0.75
	Saint Kitts and Nevis	0.75
	Belarus	0.74
	North Macedonia	0.74
	Portugal	0.74
	Antigua and Barbuda	0.74
	Barbados	0.74
	Palau	0.74
	Malaysia	0.74
	Ukraine	0.74
	Jordan	0.73

	Dominica	0.73
	Seychelles	0.72
	Kazakhstan	0.72
	Bosnia and Herzegovina	0.72
	American Samoa	0.71
	Niue	0.71
	Libya	0.71
	Lebanon	0.71
	Argentina	0.71
	Mauritius	0.71
	Georgia	0.70
	Uruguay	0.70
	Republic of Moldova	0.70
	Sri Lanka	0.69
Middle SDI (0.61-0.69)		
	Armenia	0.69
	Thailand	0.69
	China	0.69
	Panama	0.69
	Equatorial Guinea	0.69
	Jamaica	0.68
	Azerbaijan	0.68
	Albania	0.68
	Costa Rica	0.68
	South Africa	0.68
	Tunisia	0.67
	Iraq	0.67

	Turkmenistan	0.67
	Iran (Islamic Republic of)	0.67
	Saint Lucia	0.67
	Grenada	0.67
	Cuba	0.67
	Fiji	0.66
	Indonesia	0.66
	Egypt	0.66
	Gabon	0.66
	Algeria	0.65
	Mexico	0.65
	Peru	0.65
	Samoa	0.64
	Ecuador	0.64
	Brazil	0.64
	Paraguay	0.64
	Suriname	0.64
	Tonga	0.64
	Botswana	0.63
	Colombia	0.63
	Uzbekistan	0.63
	Saint Vincent and the Grenadines	0.63
	Tokelau	0.63
	Philippines	0.62
	Syrian Arab Republic	0.62
	Nauru	0.62
	Guyana	0.62

	Viet Nam	0.62
	Namibia	0.61
Low-middle SDI (0.45-0.61)		
	Venezuela (Bolivarian Republic of)	0.61
	Mongolia	0.61
	Belize	0.60
	Kyrgyzstan	0.60
	Dominican Republic	0.59
	Tuvalu	0.59
	Palestine	0.59
	Micronesia (Federated States of)	0.58
	Eswatini	0.58
	El Salvador	0.57
	Congo	0.57
	Bolivia (Plurinational State of)	0.57
	India	0.57
	Maldives	0.56
	Democratic People's Republic of Korea	0.56
	Ghana	0.56
	Morocco	0.55
	Marshall Islands	0.54
	Tajikistan	0.54
	Kiribati	0.53
	Guatemala	0.53
	Cabo Verde	0.52
	Myanmar	0.52
	Nicaragua	0.52

	Nigeria	0.52
	Sudan	0.52
	Timor-Leste	0.51
	Kenya	0.51
	Lesotho	0.51
	Zambia	0.51
	Sao Tome and Principe	0.50
	Honduras	0.50
	Mauritania	0.50
	Lao People's Democratic Republic	0.49
	Cameroon	0.49
	Vanuatu	0.48
	Bangladesh	0.48
	Zimbabwe	0.48
	Angola	0.47
	Cambodia	0.47
	Djibouti	0.46
	Bhutan	0.46
Low-SDI (0.00-0.45)		
	Comoros	0.45
	Pakistan	0.45
	Haiti	0.43
	Rwanda	0.43
	United Republic of Tanzania	0.42
	Nepal	0.42
	Togo	0.42
	Yemen	0.41

	C√¥te d'Ivoire	0.41
	Solomon Islands	0.41
	Uganda	0.40
	Gambia	0.40
	Madagascar	0.40
	Eritrea	0.40
	Papua New Guinea	0.39
	Senegal	0.39
	Malawi	0.38
	Democratic Republic of the Congo	0.38
	Liberia	0.37
	South Sudan	0.36
	Guinea-Bissau	0.36
	Benin	0.35
	Sierra Leone	0.35
	Afghanistan	0.34
	Ethiopia	0.34
	Guinea	0.32
	Mozambique	0.31
	Burundi	0.28
	Central African Republic	0.27
	Mali	0.26
	Burkina Faso	0.26
	Chad	0.24
	Niger	0.16
	Somalia	0.08

References

1 Stevens GA, Alkema L, Black RE, *et al.* Guidelines for Accurate and Transparent Health Estimates Reporting: the GATHER statement. *The Lancet* 2016; **388**: e19–23.

2 Barber RM, Fullman N, Sorensen RJD, *et al.* Healthcare Access and Quality Index based on mortality from causes amenable to personal health care in 195 countries and territories, 1990–2015: a novel analysis from the Global Burden of Disease Study 2015. *The Lancet* 2017; **390**: 231–66.

3 Fullman N, Yearwood J, Abay SM, *et al.* Measuring performance on the Healthcare Access and Quality Index for 195 countries and territories and selected subnational locations: a systematic analysis from the Global Burden of Disease Study 2016. *The Lancet* 2018; **391**: 2236–71.

4 Murray CJL, Abbafati C, Abbas KM, *et al.* Five insights from the Global Burden of Disease Study 2019. *The Lancet* 2020; **396**: 1135–59.

5 GBD 2019 Risk Factors Collaborators. The global burden of 87 risk factors for 204 countries and territories, 1990–2019: a systematic analysis for the Global Burden of Disease Study 2019. *The Lancet* 2020.

6 Vos T, Lim SS, Abbafati C, *et al.* Global burden of 369 diseases and injuries in 204 countries and territories, 1990–2019: a systematic analysis for the Global Burden of Disease Study 2019. *The Lancet* 2020; **396**: 1204–22.

7 Wang H, Abbas KM, Abbasifard M, *et al.* Global age-sex-specific fertility, mortality, healthy life expectancy (HALE), and population estimates in 204 countries and territories, 1950–2019: a comprehensive demographic analysis for the Global Burden of Disease Study 2019. *The Lancet* 2020; **396**: 1160–203.

8 Nolte E, McKee M. Measuring the health of nations: analysis of mortality amenable to health care. *BMJ* 2003; **327**: 1129.

9 Nolte E, McKee M. Does health care save lives? Avoidable mortality revisited. London: The Nuffield Trust, 2004.

10 Nolte E, McKee M. Measuring the health of nations: updating an earlier analysis. *Health Aff (Millwood)* 2008; **27**: 58–71.

11 Forouzanfar MH, Afshin A, Alexander LT, *et al.* Global, regional, and national comparative risk assessment of 79 behavioural, environmental and occupational, and metabolic risks or clusters of risks, 1990–2015: a systematic analysis for the Global Burden of Disease Study 2015. *The Lancet* 2016; **388**: 1659–724.

Part 4. Cause write-ups

Tuberculosis

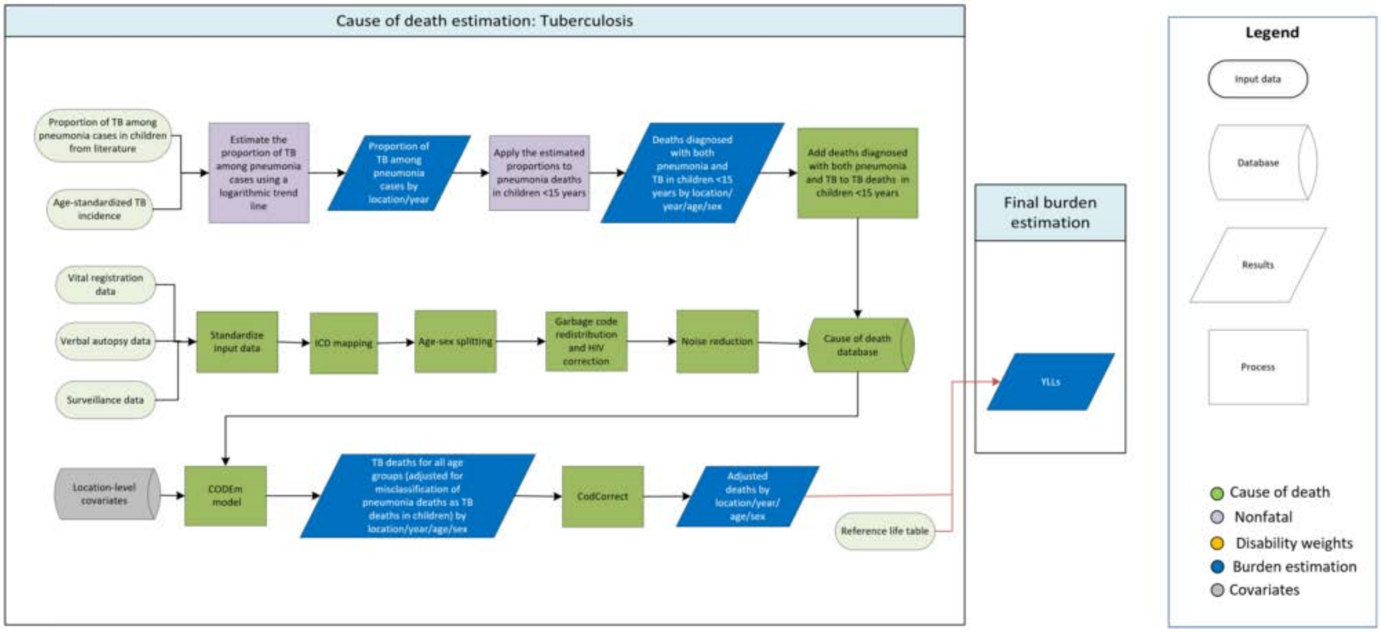

Input data

Input data for modelling tuberculosis (TB) mortality among HIV-negative individuals include vital registration, verbal autopsy, and surveillance data. Vital registration data were adjusted for garbage coding (including ill-defined codes and the use of intermediate causes) following GBD algorithms and misclassified HIV deaths (ie, HIV deaths being assigned to other underlying causes of death such as tuberculosis or diarrhoea because of stigma or misdiagnosis).

Verbal autopsy data in countries with age-standardised HIV prevalence greater than 5% were removed because of a high probability of misclassification, as verbal autopsy studies have poor validity in distinguishing HIV deaths from HIV-TB deaths.

Modelling strategy

A general CODEm modelling strategy was used. In GBD 2019, we made a small change with regard to the alcohol litres per capita covariate where we exchanged it for an all-age and both-sex equivalent that aligns better with the covariate framework for CODEm. We continued to use the TB strain prevalence-weighted transmission risk and cigarettes per capita covariate that were introduced in GBD 2017. Other location-level covariates included in the CODEm model were the same as in previous GBD cycles: adult underweight proportion, alcohol (litres per capita), diabetes (fasting plasma glucose mmol/L), education (years per capita), Healthcare Access and Quality Index, lag-distributed income, indoor air pollution, outdoor air pollution, population density, prevalence of active tuberculosis, prevalence of latent tuberculosis infection, smoking prevalence, Socio-demographic Index, and a summary exposure variable reflecting the average exposure to all of the risk factors.

Covariate table

	Covariate	Direction
Level 1	TB prevalence<br>Latent TB infection prevalence<br>SEV scalar<br>Litres of alcohol consumed per capita<br>Smoking prevalence<br>Cigarettes per capita<br>Fasting plasma glucose<br>TB strain prevalence-weighted transmission risk	+<br>+<br>+<br>+<br>+<br>+<br>+<br>+
Level 2	HAQ Index<br>Adult underweight proportion<br>Indoor air pollution<br>Outdoor air pollution<br>Population density	-<br>+<br>+<br>+<br>+
Level 3	Log LDI<br>Education (years per capita)<br>Socio-demographic Index (SDI)	-<br>-<br>-

Correcting for a potential misclassification of tuberculosis deaths as pneumonia deaths in children

Since GBD 2017, we have addressed the potential for misclassification of TB deaths as pneumonia deaths among children in locations with high TB burden. First, we estimated the proportion of tuberculosis among pneumonia cases as a function of age-standardised TB incidence using data from eight clinical studies[2,3,4,5,6,7,8,9] reporting the proportion of pneumonia cases that had tuberculosis (or the data to calculate them) and the age-standardised TB incidence estimates. We used a logarithmic trend line to fit these data. In GBD 2019, we applied the estimated proportions to pneumonia deaths reported in data among children younger than 15 years to compute the number of deaths diagnosed with both pneumonia and TB, which were then added to child TB data. Following this correction in our input data, the CODEm model was run to provide location-year-age-sex specific estimates. This is a departure from GBD 2017, where the estimated proportions were applied after CODEm. Finally, the CODEm estimates were adjusted using CoDCorrect, which ensures that the number of deaths from each cause add up to all-cause mortality deaths for a given year.

References

1. Graham SM, Sismanidis C, Menzies HJ, Marais BJ, Detjen AK, Black RE. Importance of tuberculosis control to address child survival. *Lancet* 2014; **383**(9928): 1605-7.
2. Adegbola RA, Falade AG, Sam BE, et al. The etiology of pneumonia in malnourished and well-nourished Gambian children. Pediatr Infect Dis J 1994; 13: 975–82.

3. Chisti MJ, Graham SM, Duke T, et al. A prospective study of the prevalence of tuberculosis and bacteraemia in Bangladeshi children with severe malnutrition and pneumonia including an evaluation of Xpert MTB/RIF assay. PloS One 2014; 9: e93776.
4. Madhi SA, Petersen K, Madhi A, Khoosal M, Klugman KP. Increased disease burden and antibiotic resistance of bacteria causing severe community-acquired lower respiratory tract infections in human immunodeficiency virus type 1-infected children. Clin Infect Dis 2000; 31: 170–76.
5. McNally LM, Jeena PM, Gajee K, et al. Effect of age, polymicrobial disease, and maternal HIV status on treatment response and cause of severe pneumonia in South African children: a prospective descriptive study. Lancet 2007; 369: 1440–51.
6. Moore DP, Klugman KP, Madhi SA. Role of Streptococcus pneumoniae in hospitalisation for acute community-acquired pneumonia associated with culture-confirmed Mycobacterium tuberculosis in children: a pneumococcal conjugate vaccine probe study. Pediatr Infect Dis J 2010; 29: 1099–104.
7. Nantongo JM, Wobudeya E, Mupere E, et al. High incidence of pulmonary tuberculosis in children admitted with severe pneumonia in Uganda. BMC Pediatr 2013; 13: 16.
8. Zar HJ, Hanslo D, Tannenbaum E, et al. Aetiology and outcome of pneumonia in human immunodeficiency virus-infected children hospitalized in South Africa. Acta Paediatr 2001; 90: 119–25.
9. Moore DP, Higdon MM, Hammitt LL, Prosperi C, DeLuca AN, Da Silva P, Baillie VL, Adrian PV, Mudau A, Deloria Knoll M, Feikin DR. The incremental value of repeated induced sputum and gastric aspirate samples for the diagnosis of pulmonary tuberculosis in young children with acute community-acquired pneumonia. Clinical Infectious Diseases. 2017 May 27;64(suppl_3):S309-16.

TB strain prevalence-weighted transmission risk covariate

In GBD 2017, we incorporated a TB covariate that incorporated data on the global distribution of TB strains and the relative risk of transmission associated with those strains. We continued the use of this covariate in GBD 2019. For this covariate, we defined TB strains according to the seven phylogenetic lineages of the *Mycobacterium tuberculosis* complex (MTBC) identified by S. Gagneaux and colleagues.[1] We determined the global distribution of these strains using a systematic review of human TB molecular epidemiology studies from 1990 to 2017 in PubMed and Scopus, as described in greater detail elsewhere.[2] All studies that used population-based sampling methods or collected isolates from all culture-positive TB cases in a given location and time period were included. All genotypes that could be converted to phylogenetic lineages were extracted, including genotypes determined by spoligotyping, MIRU-VNTR typing, and PCR or whole-genome sequencing. Studies of sub-populations, such as prison populations or drug-resistant cases only, were excluded. In total, 206 studies representing 85 countries and over 200,000 bacterial isolates were included. In GBD 2019, the systematic review was updated, which yielded an additional 18 studies published between 2017 and 2019. A map of these strains highlighted the widespread global distribution of Euro-American Lineage 4 strains and East Asian Lineage 2 strains, and the geographical restriction of Lineage 5 and 6 strains to West Africa. Thirty of these studies also reported transmission chains associated with bacterial genotypes, as defined by genetic clustering.[3]

We used spatiotemporal Gaussian process regression (ST-GPR) to model the distribution of each strain in each GBD location across all ages and sexes, as described in greater detail elsewhere.[4] The covariates tested in each model included HIV age-standardised prevalence, population density, and a custom-made human movement covariate. The human movement covariate took into account (1) immigration and emigration patterns[5] and (2) airplane passenger flow[6] to and from each country. In the ST-GPR models we assumed strong correlation and smoothing over both space and time. We then used a random-effects meta-analysis to determine the relative risk (RR) of transmission associated with each strain, as defined by genetic clustering. We used the most widespread strains, Euro-American Lineage 4 strains, as the reference group. We found that East Asian Lineage 2 strains were associated with increased risk of transmission overall (relative risk [95% CI] = 1.24 [1.07, 1.45]), while West African Lineage 5 and 6 strains were associated with reduced transmission (relative risk [95% CI] = 0.61 [0.43, 0.86]). We used the following formula to calculate a TB strain prevalence-weighted risk of transmission based on these estimates:

$$\sum_{i=1}^{n} Pr_i RR_i$$

i=TB strain; Pr=proportion; RR=relative risk

References

1. Comas I, Coscolla M, Luo T, *et al.* Out-of-Africa migration and Neolithic coexpansion of *Mycobacterium tuberculosis* with modern humans. *Nat Genet* 2013; **45**: 1176–82.
2. Wiens KE, Woyczynski LP, Ledesma JR, *et al*. Global variation in bacterial strains that cause tuberculosis disease: a systematic review and meta-analysis. *BMC Medicine 2018*; 16:196.
3. Dheda K, Gumbo T, Maartens G, *et al.* The epidemiology, pathogenesis, transmission, diagnosis, and management of multidrug-resistant, extensively drug-resistant, and incurable tuberculosis. *Lancet Respir Med* 2017; **5**: 291–360.
4. Manuscript in preparation.
5. United Nations Population Division. United Nations Trends in International Migrant Stock: The 2015 Revision. New York City, United States: United Nations Population Division, 2015.
6. Huang Z, Wu X, Garcia AJ, *et al*. An open-access modeled passenger flow matrix for the global air network in 2010. *PLoS ONE* **8(5)**: e64317.

Lower respiratory infections

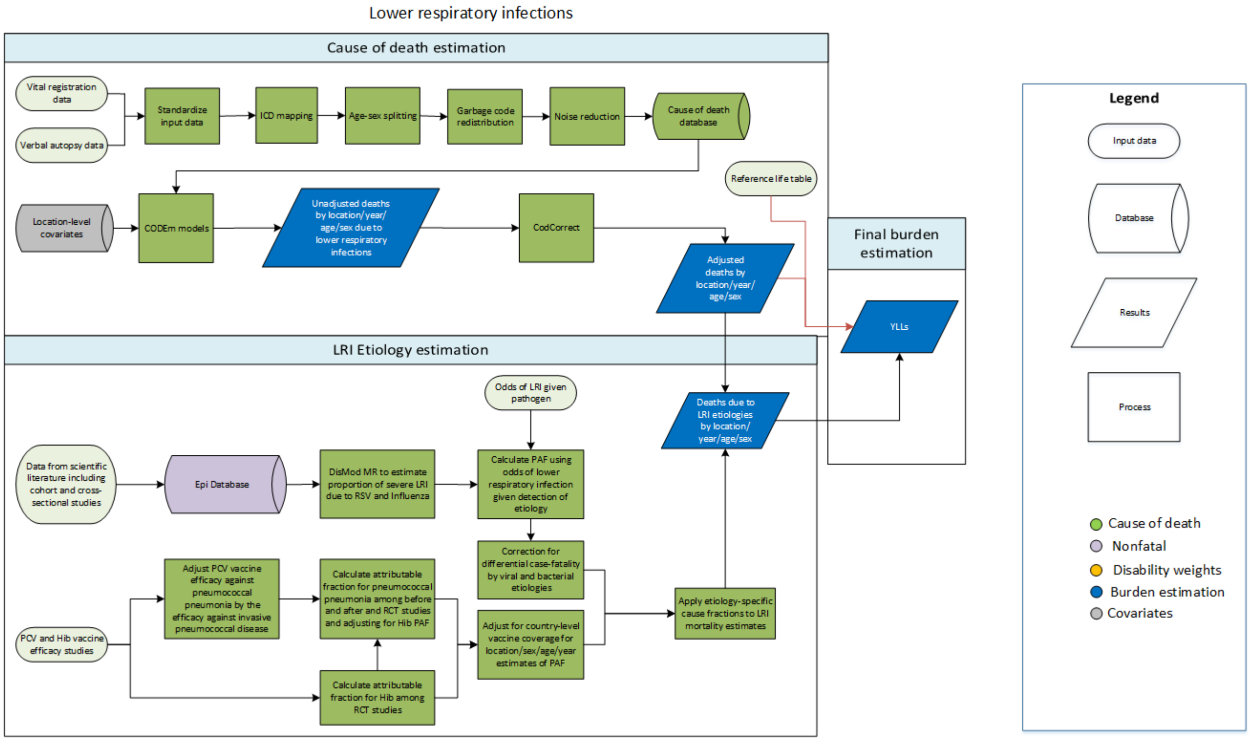

Input data

Cause of death

Lower respiratory infection (LRI) mortality was estimated in CODEm. We estimated LRI mortality separately for males and females and for children under 5 years and older than 5 years. We used all available data from vital registration systems, surveillance systems, and verbal autopsy. We checked for and excluded outliers from our data by country or region. We also excluded ICD9-coded mortality data in Sri Lanka (1982, 1987–1992), ICD9-coded neonatal mortality data in Guatemala (1980, 1981, 1984, 2000–2004), and medically coded cause of death data and Civil Registration System data in many Indian states (1986–2013).

Aetiologies

We updated our systematic review of scientific literature for the proportion of LRI that tested positive for influenza and respiratory syncytial virus (RSV) to include all data from GBD 2017 and from studies published between August 1, 2018 and February 7, 2019. We performed the search using PubMed and the following search string:

*((“lower respiratory”[title] OR pneumonia[title]) AND (**2018/08/01[PDat] : 2019/2/7[PDat]**) AND ((incidence OR prevalence OR epidemiology) OR (etiolog*[title/abstract] OR influenza[title/abstract] OR “respiratory syncytial virus”[title/abstract])) AND Humans[MeSH Terms]) NOT(autoimmune[title/abstract] OR COPD [title/abstract] OR “cystic fibrosis”[title/abstract] OR **Review[ptyp]**)*

Inclusion criteria were studies that had a sample size of at least 100, studies that were at least one year in duration, and studies describing lower respiratory infections, pneumonia, or bronchiolitis as the case definition. During our literature review we identified 121 studies, of which two met our inclusion criteria and were extracted. We excluded studies that described pandemic H1N1 influenza solely and studies that used influenza-like illness as the case definition. An age pattern based on age-specific data was estimated and then used to split data where the age range was more than 25 years.

We also conducted a systematic literature review of studies on the *Haemophilus influenzae* type B (Hib) vaccine and pneumococcal conjugate vaccine (PCV) effectiveness studies against x-ray-confirmed pneumonia and against pneumococcal and Hib disease until May 2017. This review was not updated for GBD 2019. For PCV studies, we extracted, if available, the distribution of pneumococcal pneumonia serotypes and the serotypes included in the PCV used in the study. We excluded observational and case-control studies due to implausibly high vaccine efficacy estimates. Hib trial data were exclusively from children under 5 years, so we did not include the effect of Hib on ages over 5 years. PCV trial data are also frequently limited to younger populations. To understand the contribution of pneumococcal pneumonia in older populations, we also included PCV efficacy studies that used before-after approaches.

Modelling strategy

Cause of death. LRI fatal modelling occurs using CODEm. Because of starkly different patterns, LRI CODEm models include under-5 years and 5–95+ years. Like all models of mortality in GBD, LRI mortality models are single-cause, requiring in effect that the sum of all mortality models must be equal to the all-cause mortality envelope. We correct LRI mortality estimates, and other causes of mortality, by rescaling them according to the uncertainty around the cause-specific mortality rate. This process is called CoDCorrect and is essential to ensure internal consistency among causes of death.

Table 1. Covariates used in LRI mortality modelling. Table 1A is for children under 5 and Table 1B shows the covariates used for ages 5–95+. The *Level* is the associated strength of relationship between the covariate and LRI mortality, ranked from 1 (proximally related) to 3 (distally related). *Direction* is the direction of the association between the covariate and LRI mortality.

Table 1A. Covariates used in under 5 years model

Level	Covariate	Direction
1	Childhood stunting summary exposure value (SEV)	+
	Childhood underweight SEV	+
	Childhood wasting SEV	+
	Indoor air pollution	+
	LRI SEV	+
	Antibiotics for LRI	-
	Hib vaccine coverage	-
	PCV coverage	-
	Vitamin A deficiency	+
2	Secondhand smoking prevalence	+
	Zinc deficiency	+
	DTP3 vaccine coverage	-
	Healthcare Access and Quality Index	-

	Ambient particulate matter SEV	+
	Household air pollution	+
	Outdoor air pollution ($PM_{2.5}$)	+
	Handwashing SEV	+
3	Sanitation SEV	+
	Population density > 1000/km^2	+
	Population density < 150/km^2	+
	Maternal education	-
	Socio-demographic Index	-

Table 1B. Covariates used in 5-95+ years model

Level	Covariate	Direction
1	Indoor air pollution	+
	LRI SEV	+
	Outdoor air pollution	+
	Secondhand smoking prevalence	+
	Smoking prevalence	+
2	DTP3 vaccine coverage	-
	Adult underweight	+
	Healthcare Access and Quality Index	-
	PCV coverage	-
	Handwashing access	+
3	Education years per capita	-
	Lag distributed income per capita	-
	Socio-demographic Index	-
	Sanitation SEV	+

Aetiologies

We estimated LRI aetiologies separately from overall LRI mortality using two distinct counterfactual modelling strategies to estimate population attributable fractions (PAFs), described in detail below. The PAF represents the relative reduction in LRI mortality if there was no exposure to a given aetiology. As LRIs can be caused by multiple pathogens and the pathogens may co-infect, PAFs can overlap and are not scaled to sum to 100%. Separate strategies were used for viral (influenza and RSV) and bacterial (*Streptococcus pneumoniae* and Hib) aetiologies. We did not attribute aetiologies to neonatal pneumonia deaths due to a dearth of reliable data in this age group. We calculated uncertainty of our PAF estimates from 1,000 draws of each parameter using normal distributions in log space.

Influenza and RSV. We calculated the PAF from the proportion of severe LRI cases positive for influenza and RSV. We assumed that hospitalised LRI cases are a proxy of severe cases. We used the following formula to estimate the PAF:[1]

$$PAF = Proportion\ (modelled) * (1 - \frac{1}{OR})$$

Where *Proportion* is the proportion of LRI cases that test positive for influenza or RSV and *OR* is the odds ratio of LRI given the presence of the pathogen. There are two published estimates of the odds ratios of influenza and RSV. One is based on detection in children younger than 5 years and the second is based on adults over 65 years. We applied the separate odds ratios for those age groups and log-linearly interpolated values between those ages to determine odds ratios for ages between those groups.[2, 3]

We modelled the proportion data using the meta-regression tool DisMod-MR to estimate the proportion of LRI cases that are positive for influenza and RSV, separately, by location/year/age/sex. To make disparate data types directly comparable such as the diagnostic technique (detection by PCR served as our reference), studies that investigated RSV or influenza exclusively (multi-pathogen studies were our reference), and studies from inpatient populations (community-based sample populations was our reference), we performed a meta-regression of the ratios of the reference to non-reference definitions. These meta-regression results were used to adjust the mean and variance of nonreference data. The value for the ratio of community to inpatient LRI was used as a scalar in our final estimate of fatal attributable fractions because we assumed that the frequency of influenza or RSV in hospitalised episodes of LRI represented the frequency in fatal LRI.

As the case-fatality of viral causes of pneumonia is lower than for bacterial causes, we adjusted for differential case-fatality by determining the aetiological fractions for mortality attributable to RSV and influenza (**Table 2**). We measured the aetiological fractions by applying a relative case-fatality adjustment based on in-hospital case-fatality, which we coded to specific pneumonia aetiologies. Hospital admissions data of this type were limited to data from Austria, Brazil, Chile, China, Ecuador, Italy, Kenya, Mexico, New Zealand, the Philippines, Portugal, and the United States. We generated the pooled estimate of the case-fatality differential between bacterial (pneumococcus, Hib) and viral aetiologies (RSV, influenza) using DisMod-MR to determine an age pattern for this ratio. Therefore, the final attributable fraction for fatal LRI was:

$$Fatal\ PAF = Proportion * \left(1 - \frac{1}{OR}\right) * Inpatient\ scalar * Case\ fatality\ scalar$$

Pneumococcal pneumonia and Hib. For *Streptococcus pneumoniae* (pneumococcal pneumonia) and Hib, we calculated the PAF using a vaccine probe design.[4,5] The ratio of vaccine effectiveness against nonspecific pneumonia to pathogen-specific disease represents the fraction of pneumonia cases attributable to each pathogen.

To estimate the PAF for Hib and pneumococcal pneumonia, we calculated the ratio of vaccine effectiveness against nonspecific pneumonia to pathogen-specific pneumonia (equations 1 and 3). We estimated a study-level estimate of the PAF from a meta-analysis of these ratios. To estimate the PAF for Hib, we only used randomised controlled trials because of implausibly high values of vaccine efficacy in case-control studies. To estimate the PAF for pneumococcal pneumonia, we included RCTs and before and after vaccine introduction longitudinal studies.

We adjusted the study-level PAF estimate by vaccine coverage and expected vaccine performance to estimate country- and year-specific PAF values. For pneumococcal pneumonia, we adjusted the PAF by the final Hib PAF estimate and by vaccine serotype coverage. Finally, we used an age distribution of the PAF modelled in DisMod to determine the PAF by age. Because of an absence of data describing vaccine

efficacy against Hib in children older than 2 years, we did not attribute Hib to episodes of LRI in ages 5 years and older.

We used a vaccine probe design to estimate the PAF for pneumococcal pneumonia and Hib by first calculating the ratio of vaccine effectiveness against nonspecific pneumonia to pathogen-specific pneumonia at the study level (equations 1 and 2).[4–6] We then adjusted this estimate by vaccine coverage and expected vaccine performance to estimate country- and year-specific PAF values (equations 3 and 4).

$$1)\quad HibPAF_{Base} = \frac{VE_{Pneumonia}}{VE_{Hib}}$$

$$2)\quad PneumoPAF_{Base} = \frac{VE_{Pneumonia} * (1 - PAF_{Hib} * VE_{Hib\ Optimal})}{VE_{Streptococcus} * Cov_{Serotype}}$$

$$3)\quad PAF_{Hib} = PAF_{Base} * \frac{(1 - Cov_{Hib} * VE_{Hib\ Optimal})}{(1 - PAF_{Base} * Cov_{Hib} * VE_{Hib\ Optimal})}$$

$$4)\quad PAF_{Pneumo} = \frac{PAF_{Base} * (1 - Cov_{PCV} * VE_{PCV\ Optimal})}{(1 - PAF_{Hib} * Cov_{Hib} * VE_{Hib\ Optimal}) * \left(1 - \frac{PAF_{Base} Cov_{PCV} * VE_{PCV\ Optimal}}{(1 - PAF_{Hib} * Cov_{Hib} * VE_{Hib\ Optimal})}\right)}$$

Where $VE_{Pneumonia}$ is the vaccine efficacy against nonspecific pneumonia, VE_{Hib} is the vaccine efficacy against invasive Hib disease, $VE_{Streptococcus}$ is the vaccine efficacy against serotype-specific pneumococcal pneumonia, $Cov_{serotype}$ is the serotype-specific vaccine coverage for PCV,[7] $VE_{Hib\ Optimal}$ is the Hib effectiveness in the community (0.8),[8] PAF_{Hib} is the final PAF for Hib, Cov_{PCV} is the PCV coverage, Cov_{Hib} is the Hib coverage by country, and $VE_{PCV\ Optimal}$ is the vaccine effectiveness in the community (0.8).[9]

For Hib, we assumed that the vaccine efficacy against invasive Hib disease is the same against Hib pneumonia. For pneumococcal pneumonia, a recent study in adults[10] found that the vaccine efficacy against invasive pneumococcal disease may be significantly higher than against pneumococcal pneumonia. We used this ratio to adjust estimates of vaccine efficacy against invasive pneumococcal disease from other studies. However, recognising that the study is unique in that it uses a urine antigen test among adults, we added uncertainty around our adjustment using a wide uniform distribution (median 0.65, 0.3–1.0). This has increased the estimates of pneumococcal pneumonia mortality in a meaningful way.

Table 2: **The median values for the ratio of viral to bacterial pneumonia case-fatality ratio by age is shown.** These estimates are modelled using hospital-based, ICD-coded admissions and mortality for aetiology-specified pneumonia. Values in parentheses represent 95% uncertainty interval.

Age group	Ratio
Early neonatal	0.59 (0.36–0.84)
Late neonatal	0.58 (0.37–0.84)
Post neonatal	0.58 (0.41–0.77)
1 to 4	0.69 (0.64–0.74)
5 to 9	0.85 (0.77–0.93)
10 to 14	0.84 (0.79–0.89)
15 to 19	0.83 (0.78–0.87)
20 to 24	0.82 (0.77–0.87)
25 to 29	0.82 (0.78–0.86)
30 to 34	0.82 (0.79–0.85)
35 to 39	0.82 (0.8–0.85)
40 to 44	0.82 (0.8-0.85)
45 to 49	0.82 (0.8–0.85)
50 to 54	0.82 (0.79–0.85)
55 to 59	0.82 (0.79–0.86)
60 to 64	0.82 (0.79–0.86)
65 to 69	0.82 (0.8–0.85)
70 to 74	0.82 (0.79–0.85)
75 to 79	0.82 (0.78–0.85)
80 to 84	0.83 (0.8–0.87)
85 to 89	0.86 (0.83–0.89)
90 to 94	0.89 (0.85–0.93)
95 to 99	0.92 (0.86–0.97)

Changes from GBD 2017

The main changes from GBD 2017 involved methods used in determining the attributable fractions for influenza and RSV. For GBD 2019, we applied a consistent and reproducible approach to estimating the ratio of reference to nonreference data. For example, we found the ratio of the proportion of LRI that tested positive for RSV among community episodes and divided that by the proportion positive in inpatient populations.

$$\frac{Proportion_{Community}}{Proportion_{Inpatient}}$$

This value was the input in a meta-regression to find the mean relative difference in those values. This scalar was used to adjust all inpatient data to the *expected* value if it used a community sample instead. The approach described here was used to make inpatient, non-PCR, and single etiology studies more similar to our reference definitions.

The second main change implemented in GBD 2019 was the differential odds ratios by age. Previously, we used a single study of the odds ratio of influenza and RSV for children younger than 5 and applied that to all ages. With a recently published article on the odds for these pathogens in adults over 65 years, we were able to have different values by age.

References

1 Miettinen OS. Proportion of disease caused or prevented by a given exposure, trait or intervention. *Am J Epidemiol* 1974; **99**: 325–32.

2 Shi T, McLean K, Campbell H, Nair H. Aetiological role of common respiratory viruses in acute lower respiratory infections in children under five years: A systematic review and meta-analysis. *J Glob Health* 2015; **5**: 10408.

3 Shi T, Arnott A, Semogas I, Falsey AR, Openshaw P, Wedzicha JA, Campbell H, Nair H, RESCEU Investigators. The etiological role of common respiratory viruses in acute respiratory infections in older adults: a systematic review and meta-analysis. J Infect Dis. 2019 Mar 8. doi: 10.1093/infdis/jiy662

4 Feikin DR, Scott JAG, Gessner BD. Use of vaccines as probes to define disease burden. *Lancet Lond Engl* 2014; **383**: 1762–70.

5 O'Brien KL, Wolfson LJ, Watt JP, *et al.* Burden of disease caused by Streptococcus pneumoniae in children younger than 5 years: global estimates. *Lancet Lond Engl* 2009; **374**: 893–902.

6 Watt JP, Wolfson LJ, O'Brien KL, *et al.* Burden of disease caused by Haemophilus influenzae type b in children younger than 5 years: global estimates. *Lancet Lond Engl* 2009; **374**: 903–11.

7 Johnson HL, Deloria-Knoll M, Levine OS, *et al.* Systematic evaluation of serotypes causing invasive pneumococcal disease among children under five: the pneumococcal global serotype project. *PLoS Med* 2010; **7**. DOI:10.1371/journal.pmed.1000348.

8 Swingler G, Fransman D, Hussey G. Conjugate vaccines for preventing Haemophilus influenzae type B infections. *Cochrane Database Syst Rev* 2007; : CD001729.

9 Lucero MG, Dulalia VE, Nillos LT, *et al.* Pneumococcal conjugate vaccines for preventing vaccine-type invasive pneumococcal disease and X-ray defined pneumonia in children less than two years of age. *Cochrane Database Syst Rev* 2009; : CD004977.

10 Bonten MJM, Huijts SM, Bolkenbaas M, *et al.* Polysaccharide conjugate vaccine against pneumococcal pneumonia in adults. *N Engl J Med* 2015; **372**: 1114–25.

Upper respiratory infections

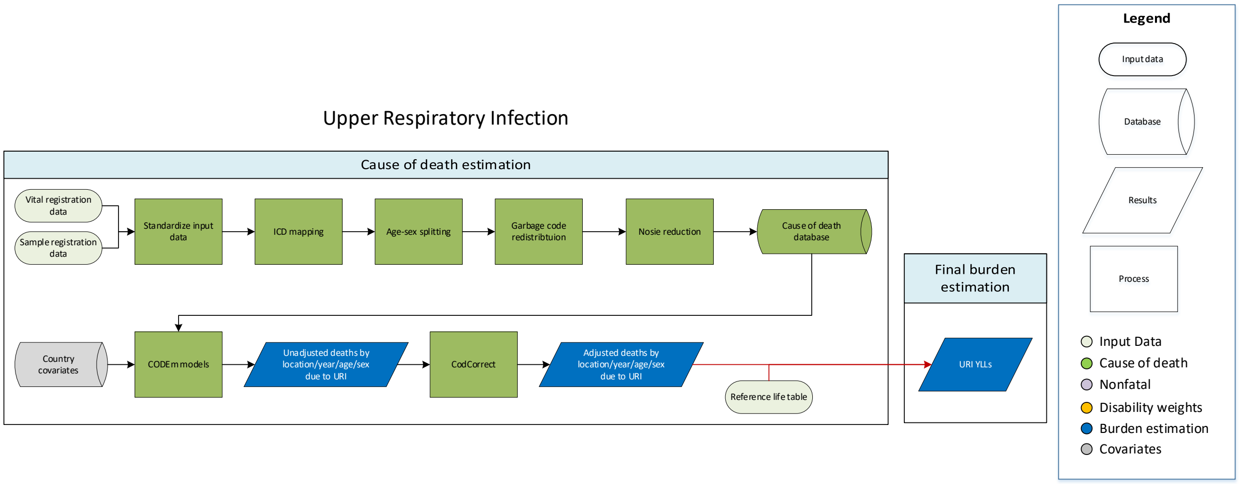

Input data and methodological summary for upper respiratory infections

Input data

Vital registration and surveillance data from the cause of death (CoD) database were used. Outliers were identified by systematic examination of datapoints. Datapoints that violated well-established age or time trends, were inconsistent with other country- or region-specific points, or that resulted in extremely high or low mortality rates were determined to be outliers.

Modelling strategy

A generic CODEm approach was used to estimate mortality due to upper respiratory infections (URI) in GBD 2019. In GBD 2016, mortality from URI was modelled using a negative binomial regression. It was determined that a negative binomial regression was an appropriate approach for estimating URI due to a small number of deaths due to URI in the CoD database. However, due to changes in how we redistribute cause of death codes, more deaths were attributed to URI in the CoD database, and thus it was determined that a generic CODEm approach was feasible for estimating URI mortality in GBD 2017. The covariates used are displayed below. We have made no substantive changes to the modelling strategy in 2019.

Level	Covariate	Direction
1	Smoking prevalence	+
2	Indoor pollution	+
	Outdoor pollution ($PM_{2.5}$)	+
	Healthcare Access and Quality Index	-
3	Socio-demographic Index	-
	Lag distributed income	-
	Education (years per capita)	-

Diarrhoeal diseases

Flowchart

Diarrheal diseases

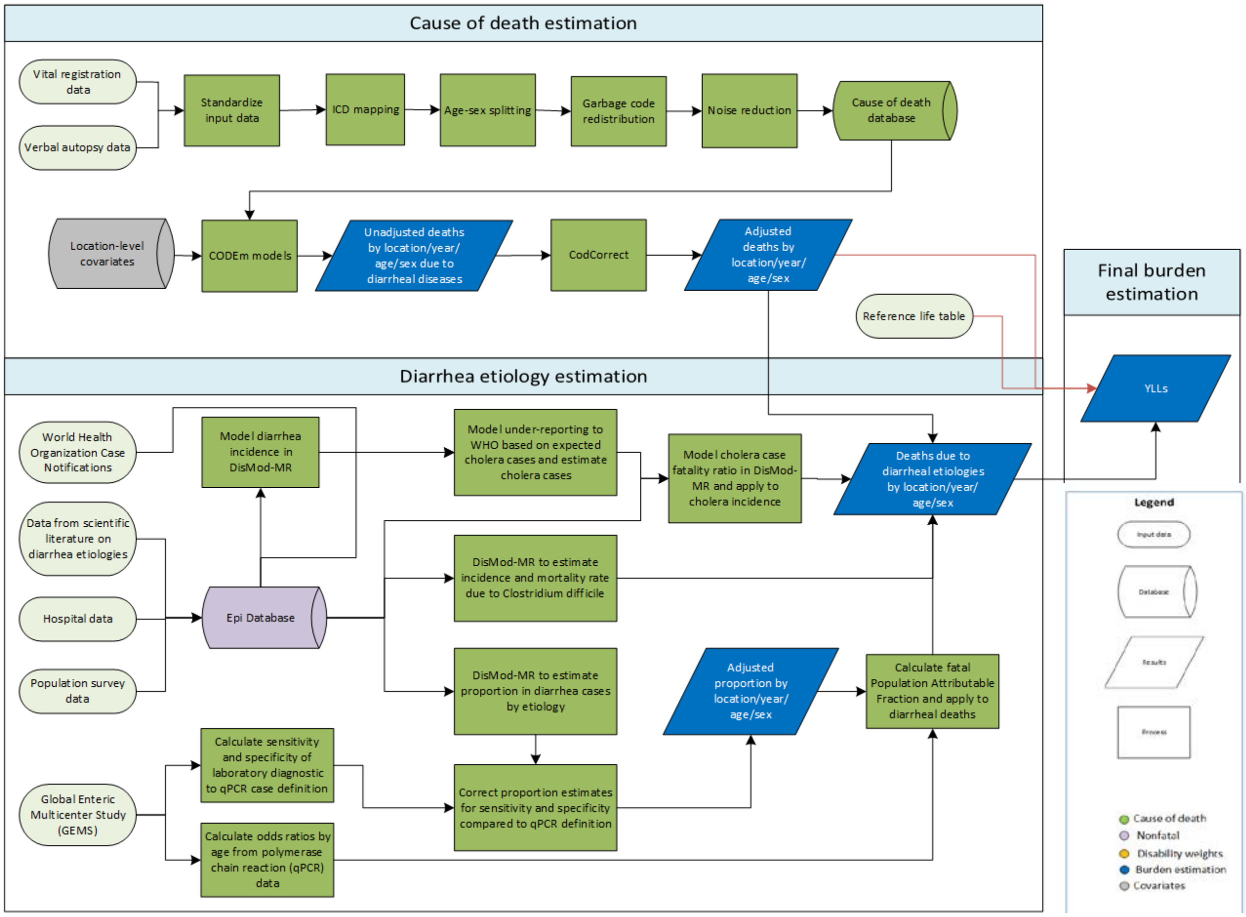

Diarrhoeal diseases are a cause of death in GBD. We also estimated the attributable deaths from 13 diarrhoeal aetiologies using an independent modelling strategy. These pathways are shown in the flowchart above and will both be described in this report.

Input data

Cause of death. We used all available data from vital registration systems, surveillance systems, and verbal autopsy. Data points that violated well-established age or time trends were determined to be outliers. We also excluded early neonatal mortality data in the Philippines (1994–1998), India Civil Registration System data, and medically certified cause of death (MCCD) data in all states (1986–2013).

Aetiologies. The second type of data describes diarrhoea aetiologies. There are 13 aetiologies in GBD 2019 for diarrhoea: adenovirus, *aeromonas, campylobacter, vibrio cholerae, clostridium difficile, cryptosporidium*, *entamoeba histolytica,* typical enteropathogenic *E. coli* (typical EPEC)*,* heat-stable toxin producing enterotoxigenic *E. coli* (ST-ETEC)*,* norovirus, rotavirus, non-

typhoidal salmonella, and shigella. We extracted data on all aetiologies except *C. difficile* from scientific literature that reported the proportion of diarrhoea cases that tested positive for each pathogen. We completed a systematic literature review covering the time period May 2018 to February 2019 for diarrhoea prevalence, incidence, and all diarrhoea aetiologies. Inclusion criteria included diarrhoea as the case definition, studies with a sample size of at least 100, and studies with at least one year of follow up. We excluded studies that reported on diarrhoeal outbreaks exclusively and those that used acute gastroenteritis with or without diarrhoea.

We searched articles using a PubMed search term that combined nonspecific and aetiology-specific diarrhoea in February 2019 using the following search string:

(diarrhoea[title/abstract] OR diarrhea[title/abstract]) AND (2018/07/30:2019/2/7[PDat]) AND Humans[MeSH Terms] AND (incidence[title/abstract] OR prevalence[title/abstract] OR epidemiology[title/abstract] OR salmonella[title/abstract] OR aeromona[title/abstract] OR shigell*[title/abstract] OR enteropathogenic[title/abstract] OR enterotoxigenic[title/abstract] OR campylobacter[title/abstract] OR amoebiasis[title/abstract] OR entamoeb*[title/abstract] OR cryptosporid*[title/abstract] OR rotavirus[title/abstract] OR norovirus[title/abstract] OR adenovirus[title/abstract] OR etiology[title/abstract]) NOT (appendicitis[title/abstract] OR esophag*[title/abstract] OR surger*[title/abstract] OR gastritis[title/abstract] OR liver[title/abstract] OR case report[title] OR case-report[title] OR therapy[title] OR treatment[title] Crohn[title/abstract] OR "inflammatory bowel"[title/abstract] OR irritable[title/abstract] OR travel*[title] OR Outbreak[title] OR Review[ptyp] OR vomiting[title/abstract).*

We identified 82 studies, of which three met our inclusion criteria. We extracted data for location, sex, year, and age.

We used the Global Enteric Multicenter Study (GEMS), a seven-site, case-control study of moderate-to-severe diarrhoea in children under 5 years,[1] and the MAL-ED study,[2] a multi-site birth cohort, to calculate odds ratios for the diarrhoeal pathogens. We analysed raw data for a systematic reanalysis, representative of the distribution of cases and controls by age and site that were tested for the presence of pathogen using quantitative polymerase chain reaction (qPCR).[3]

Data that did not use qPCR for detection were adjusted for sensitivity and specificity prior to modelling in order to standardize data regardless of detection method. Adjusting these data prior to modelling allowed us to adjust only data that did not use qPCR, as well as better control for values at extreme bounds, and capture uncertainty in modelling.

Modelling strategy

Cause of death. Diarrhoeal disease mortality was estimated in the Cause of Death Ensemble modelling platform (CODEm). We estimated diarrhoea mortality separately for males and

females and for children under 5 years and older than 5 years. We used country-level covariates to inform our CODEm models (**Table 1**).

Table 1. The covariates used in diarrhoea mortality modelling. Table 1A shows the covariates used in the 0–4 years model, and Table 2B shows the covariates used in the 5–95+ years model. The *Level* represents the strength of the association between the covariate and diarrhoea mortality from 1 (proximally related) to 3 (distally related). The *Direction* indicates the positive or negative association between the covariate and diarrhoea mortality.

Table 1A. The covariates used in the 0–4 years model

Level	Covariate	Direction
1	Oral rehydration solution treatment	-
	Safe sanitation access	-
	Safe water access	-
	Rotavirus vaccine	-
2	Vitamin A deficiency	+
	Zinc deficiency	+
	Zinc treatment for diarrhoea	-
3	Handwashing access	-
	Lag distributed income (LDI) per capita	-
	Maternal education years	-
	Healthcare Access and Quality Index	-
	Socio-demographic Index (SDI)	-

Table 1B. The covariates used in the 5–95+ years model.

Level	Covariate	Direction
1	Diarrhoea summary exposure value (SEV)	+
	Unsafe sanitation SEV	+
	Unsafe water SEV	+
	Sanitation access	-
	Improved water source access	-
2	Healthcare Access and Quality Index	-
	Rotavirus vaccine coverage	-
3	Education years per capita	-
	LDI per capita	-
	Adult underweight	+
	SDI	-
	Oral rehydration accesss	-
	Population density less than 150/km^2	+
	Population density greater than 1000/km^2	+

Aetiologies. We estimated diarrhoeal disease aetiologies independently from overall diarrhoea mortality using a counterfactual strategy for enteric adenovirus, *aeromonas*, *entamoeba histolytica* (amoebiasis), *campylobacter*, *cryptosporidium*, typical EPEC, enterotoxigenic *Escherichia coli* (ETEC), norovirus, non-typhoidal salmonella infections, rotavirus, and shigella. *Vibrio cholerae* and *C. difficile* were modelled separately.

Diarrhoeal aetiologies are attributed to diarrhoeal deaths using a counterfactual approach. We calculated a population attributable fraction (PAF) from the proportion of severe diarrhoea cases that are positive for each aetiology. The PAF represents the relative reduction in diarrhoea mortality if there was no exposure to a given aetiology. As diarrhoea can be caused by multiple pathogens and the pathogens may co-infect, PAFs can overlap and are not scaled to sum to 100%. We calculated the PAF from the proportion of severe diarrhoea cases that are positive for each aetiology. We assumed that hospitalised diarrhoea cases are a proxy of severe and fatal cases. We used the following formula to estimate PAF:[4]

$$PAF = Proportion * (1 - \frac{1}{OR})$$

Where *Proportion* is the proportion of diarrhoea cases positive for an aetiology and *OR* is the odds ratio of diarrhoea given the presence of the pathogen.

We dichotomised the continuous qPCR test result using the value of the cycle threshold (Ct) that most accurately discriminated between cases and controls. The Ct values range from 0 to 35 cycles representing the relative concentration of the target gene in the stool sample. A low value indicates a higher concentration of the pathogen while a value of 35 indicates the absence of the target in the sample. We used the lower Ct value when we had multiple Ct values for the cutpoint. The case definition for each pathogen is a Ct value that is below the established cutoff point.

We used a mixed effects conditional logistic regression model to calculate the odds ratio for under 1 year and 1–4 years old for each of our pathogens. The stool samples from cases and controls in GEMS were used exclusively to calculate these odds ratios as we assumed that the association between pathogens and moderate-to-severe diarrhoea is a proxy for fatal outcomes. The odds ratio for 1–4 years was applied to all GBD age groups over 5 years. There were three pathogen-age odds ratios that were not statistically significant: aeromonas and amoebiasis in under 1 year and campylobacter in 1–4 years. The mean value of the odds ratio was above 1 in all three cases, so we transformed the odds ratios for these three exceptions only in log space such that exponentiated values could not be below 1. The transformation was:

Odds ratio = exp(log(OR) – 1)) + 1

We modelled the proportion data using the Bayesian meta-regression tool DisMod-MR to estimate the proportion of positive diarrhoea cases for each separate aetiology by location/year/age/sex and to adjust for the covariates. We used the estimated sensitivity and specificity of the original laboratory diagnostic test results from the pooled GEMS and MAL-ED qPCR stool samples compared to the qPCR test result to adjust our proportion before we modelled the proportions:[5]

$$Proportion_{True} = \frac{(Proportion_{Observed} + Specificity - 1)}{(Sensitivity + Specificity - 1)}$$

We used this correction to account for the fact that the proportions we used are based on a new test that is not consistent with the laboratory-based case definition (qPCR versus GEMS conventional laboratory testing for pathogens).[6] Because differences in the type of PCR used in the original (nonreference qPCR diagnostic) between GEMS and MAL-ED in detecting norovirus, we combined the sensitivity and specificity results for norovirus such that 50% of the draws were coming from GEMS test results exclusively and 50% of the draws were coming from MAL-ED test results exclusively. Additionally, because the original laboratory diagnostic technique used for *campylobacter* in MAL-ED was one not commonly used, we only used GEMS to determine the sensitivity and specificity of bacterial culture compared to qPCR in detecting *campylobacter.*[7]

Our literature review extracted the proportion of any EPEC without differentiating between typical (tEPEC) and atypical (aEPEC). In order to be consistent with the odds ratios that we obtained, we adjusted our proportion estimates of any EPEC to typical EPEC only. This adjustment was informed by a subset of our literature review that reported both atypical and typical EPEC. We estimated a ratio by super-region of tEPEC to any EPEC and adjusted our proportion estimates accordingly. We found that the majority of EPEC diarrhoea cases were positive for atypical EPEC, consistent with other published work.[8] We applied the same approach to differentiate between heat-stable toxin (ST) and heat labile toxin producing (LT) ETEC. For the first time, GBD 2019 split these serotypes so that estimates in GBD 2019 represent the diarrhoeal disease burden attributable to ST-ETEC. This was based on work showing that ST-ETEC was much more pathogenic than LT-ETEC. As our proportion data were extracted for any ETEC, we determined a proportion of all ETEC that produced ST from the GEMS and MAL-ED studies and applied that ratio to our input data so that they represented ST-ETEC only. We re-estimated the sensitivity and specificity values as well as the odds ratios for our new definition of ST-ETEC.

For *vibrio cholerae* (cholera), we used the literature review to estimate the expected number of cholera cases for each country-year using the incidence of diarrhea (estimated using DisMod-MR) and the proportion of diarrhoea cases that are positive for cholera. We assigned cholera PAF using odds ratios from the qPCR results to estimate a number of cholera-attributable cases. We compared this expected number of cholera cases to the number reported to the World Health Organization at the country-year level.[9] We modelled the underreporting fraction to correct the cholera case notification data for all countries using health system access and the diarrhoea SEV scalar to predict total cholera cases. We used the age-specific proportion of positive cholera samples in DisMod-MR and our incidence estimates to predict the number of cholera cases for each age/sex/year/location. Finally, we modelled the case fatality ratio of cholera using DisMod-MR and to estimate the number of cholera deaths.

For *C. difficile*, we modelled incidence and mortality in DisMod-MR for each age, sex, year, location. DisMod-MR is a Bayesian meta-regression tool that uses spatiotemporal information

as priors to estimate prevalence, incidence, remission, and mortality for *C. difficile* infection. DisMod-MR uses a compartmental model to relate prevalence, incidence, remission, and mortality. We set remission in our model to 1 month.

For rotavirus, we made a change to the process of estimating attributable fraction to explicitly account for rotavirus vaccine efficacy in GBD 2019. The impact of the rotavirus vaccine is dependent on modelled vaccine coverage for a location-year and on the rotavirus vaccine efficacy (VE). There are numerous studies that demonstrate a difference in VE by location.[10] We determined that SDI was the best predictor of rotavirus VE, and we used a meta-regression with this covariate to predict the rotavirus VE by location where the VE was higher in areas with larger SDI values and followed a logit-linear distribution.

For GBD 2019, we explicitly incorporated the results from our analysis of VE to produce more robust estimates of the proportion of diarrhoea that has rotavirus over time and space. We assumed that the impact of the vaccine can be represented as one minus the product of the estimated vaccine coverage and VE.

$$Vaccine\ impact = 1 - vaccine\ coverage * vaccine\ efficacy$$

Both of these values vary in time and space but not by age. To avoid discontinuities in our model, we adjusted the input proportion data to remove the impact of the rotavirus vaccine by dividing the observed proportion by the vaccine impact.

$$Rotavirus\ proportion_{Adjusted} = \frac{Rotavirus\ proportion}{1 - Cov_{RotaV} * VE_{Modeled}}$$

The result is the modelled proportion of diarrhoea positive for rotavirus in the absence of the vaccine. This modelled value is then multiplied by the impact of the rotavirus vaccine to determine the estimated proportion of diarrhoea positive for rotavirus in the presence of the vaccine. Our modified attributable fraction is then:

$$DisModPAF = Modeled\ Proportion\ (from\ DisMod)\ * \left(1 - \frac{1}{OR}\right)$$

The last step is to account for the expected impact of the rotavirus vaccine. We do this using the equation below:

$$PAF_{Rota} = DisModPAF * \frac{(1 - Cov_{RotaV} * VE_{Modeled})}{(1 - DisModPAF * Cov_{RotaV} * VE_{Modeled})}$$

Where the final attributable fraction for rotavirus is the product of the PAF estimated in DisMod-MR and the expected reduction in that PAF given modelled vaccine coverage and modelled VE by location-year, and this value is only applied to children 28 days to 5 years old. The product of the rotavirus attributable fraction and the number of deaths or cases of diarrhoea is the number of deaths and cases caused by rotavirus.

References

1 Kotloff KL, Nataro JP, Blackwelder WC, *et al.* Burden and aetiology of diarrhoeal disease in infants and young children in developing countries (the Global Enteric Multicenter Study, GEMS): a prospective, case-control study. *Lancet Lond Engl* 2013; **382**: 209–22.

2 Platts-Mills J, Liu J, Rogawski E. Aetiology, burden and clinical characteristics of diarrhoea in children in low-resource settings using quantitative molecular diagnostics: results from the MAL-ED cohort study. *Lancet Glob Health* 2018; : Accepted.

3 Liu J, Gratz J, Amour C, *et al.* A laboratory-developed TaqMan Array Card for simultaneous detection of 19 enteropathogens. *J Clin Microbiol* 2013; **51**: 472–80.

4 Miettinen OS. Proportion of disease caused or prevented by a given exposure, trait or intervention. *Am J Epidemiol* 1974; **99**: 325–32.

5 Reiczigel J, Földi J, Ozsvári L. Exact confidence limits for prevalence of a disease with an imperfect diagnostic test. *Epidemiol Infect* 2010; **138**: 1674–8.

6 Platts-Mills JA, Operario DJ, Houpt ER. Molecular diagnosis of diarrhea: current status and future potential. *Curr Infect Dis Rep* 2012; **14**: 41–6.

7 Platts-Mills JA, Liu J, Gratz J, *et al.* Detection of Campylobacter in stool and determination of significance by culture, enzyme immunoassay, and PCR in developing countries. *J Clin Microbiol* 2014; **52**: 1074–80.

8 Ochoa TJ, Barletta F, Contreras C, Mercado E. New insights into the epidemiology of enteropathogenic Escherichia coli infection. *Trans R Soc Trop Med Hyg* 2008; **102**: 852–6.

9 World Health Organization. Global Health Observatory data repository: Cholera. 2016. http://apps.who.int/gho/data/node.main.174?lang=en (accessed Aug 25, 2016).

10 Lamberti LM, Ashraf S, Walker CLF, Black RE. A Systematic Review of the Effect of Rotavirus Vaccination on Diarrhea Outcomes Among Children Younger Than 5 Years. *Pediatr Infect Dis J* 2016; **35**: 992–8.

Diphtheria

Flowchart

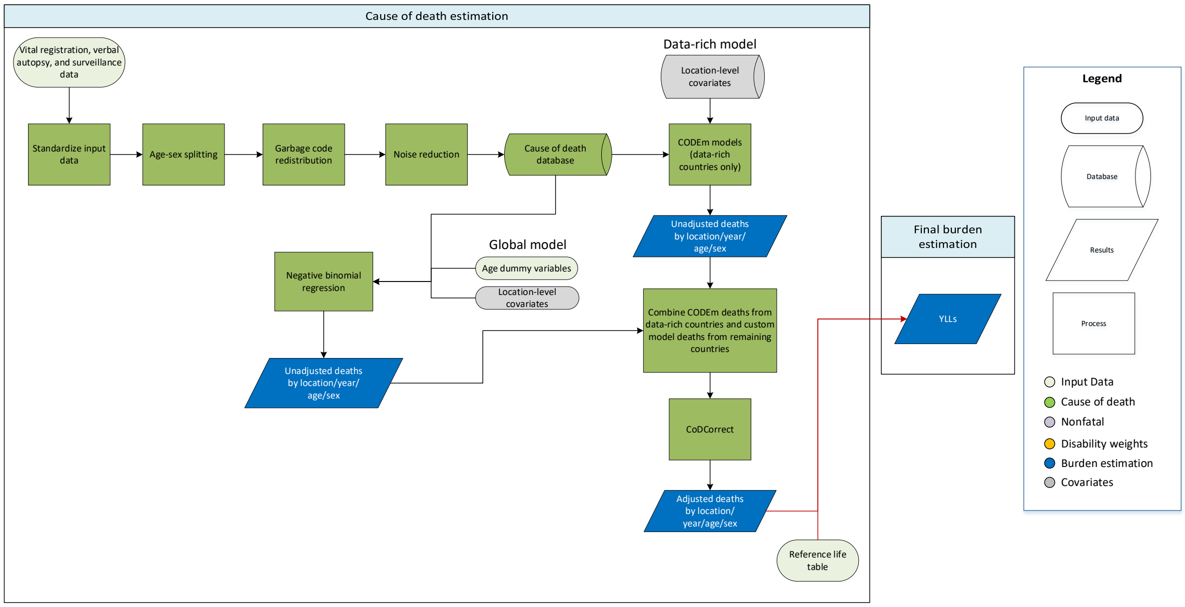

Input data

Diphtheria cause of death (COD) data for GBD 2019 included vital registration, verbal autopsy, and surveillance sources from all locations as available. We excluded COD data if they were highly incongruent with other available data from the same location or locations with similar sociodemographic characteristics.

Modelling strategy

We used two distinct methods to estimate diphtheria mortality for different countries based on the quality of vital registration data available. We used a counts-based Cause of Death Ensemble modeling strategy (CODEm) for countries with well-defined vital registration (ie, “data-rich” countries), and for remaining countries a custom count negative binomial regression model. Each approach is further described in more detail below.

1. Data-rich countries

We used CODEm counts models rather than standard rate-space CODEm models, as the models in count space had lower out-of-sample root mean squared error (RMSE) than those in rate-space. For data-rich locations, we used the covariates outlined in Table 1 to inform CODEm predictions. New covariates in the GBD 2019 models were age- and sex-specific summary exposure values (SEV) for child wasting to replace the wasting proportion covariate; Healthcare Access and Quality (HAQ) Index and Socio-

demographic Index (SDI) were used to capture the effect of the maternal care and immunisation (MCI) covariate used in prior GBD cycles.

Table 1. Covariates. Summary of covariates used in the data-rich diphtheria cause of death model

Level	Covariate	Direction
1	Diphtheria-tetanus-pertussis third-dose vaccination coverage (DTP3)	-
	Healthcare Access and Quality (HAQ) Index	-
	Age- and sex-specific SEV for child wasting	+
3	Lag-distributed income (LDI)	-
	Socio-demographic Index (SDI)	-
	Mean years of education per capita	-

2. Custom count model

Our custom counts mortality model for all non-data-rich locations also used COD data as available by location. We excluded data with extremely high cause fractions (ie, greater than the 99th percentile of all diphtheria cause fractions). Using a negative binomial regression with a log link, cause fractions representing the number of deaths due to diphtheria as a proportion of the all-cause mortality envelope were regressed using five-year rolling diphtheria-pertussis-tetanus third-dose (DPT3) vaccine coverage as a covariate, with dummy variables for each GBD age group as predictors:

$$Y_{ij} = \beta_0 + \beta_1 \, DTP3_{ij} + \beta_a \, age_a + e_{ij} \, ,$$

where Y_{ij} is the log-transformed cause fraction (counts of deaths with an offset of the total number of deaths); β_0 is the fixed-effect intercept; β_1 is the fixed-effects slope on vaccine coverage; β_a is the fixed-effects slope on age_a, the dummy variable for each GBD age group in the estimation; e_{ij} is the residual; i is the year; and j is the location. In past GBD cycles, estimates of routine DTP3 coverage among infants in the modeled year were used as the routine immunization input into this model rather than the average DTP3 coverage over the previous five years.

Uncertainty was estimated by predicting 1000 draws based on the variance-covariance matrix, and a random sample of the dispersion parameter from a gamma distribution. Results were summarised as the mean of all draws and an associated 95% uncertainty interval (the 2.5th and 97.5th quantile of all draws).

Pertussis (whooping cough)

Flowchart

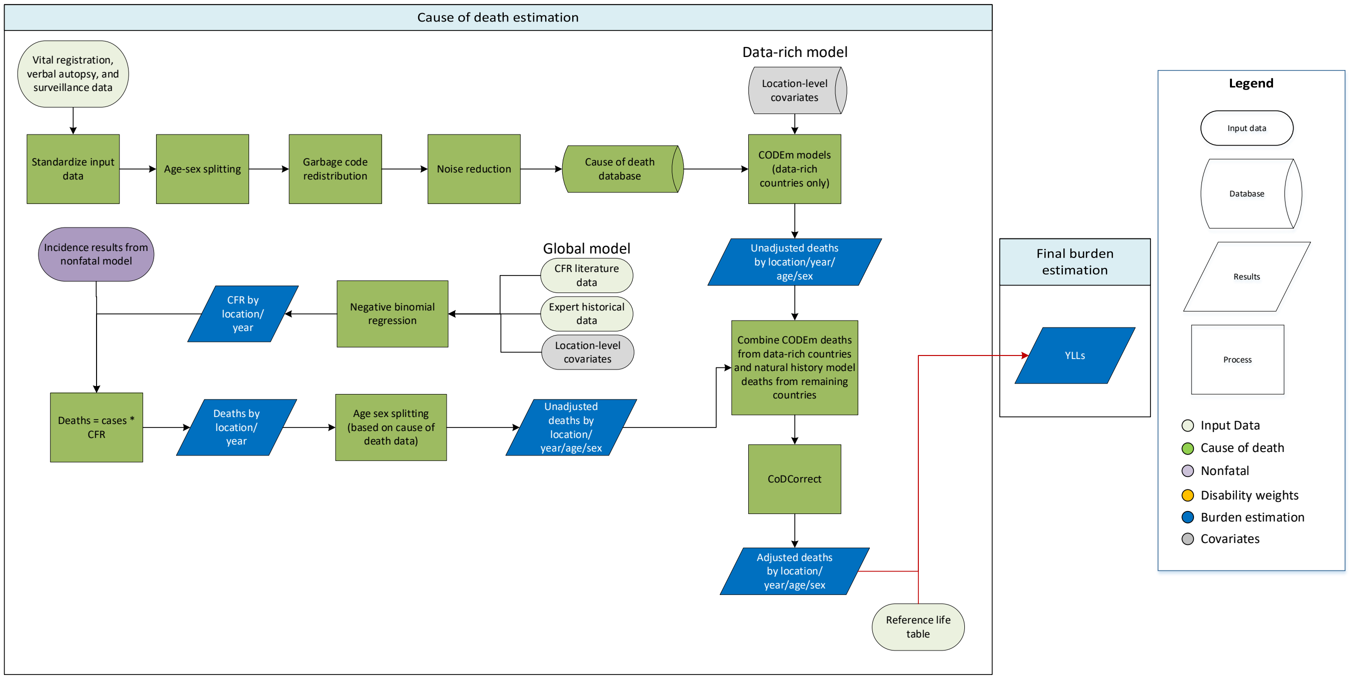

Modelling strategy overview

The GBD 2019 pertussis mortality estimates were generated one of two ways depending on the quality of available vital registration data for the country. For countries with well-defined vital registration (ie, "data-rich" countries), we used a Cause of Death Ensemble model (CODEm). For the remaining countries, we leveraged a natural history model approach, drawing from preceding non-fatal case estimates. For all countries, we made estimates for all age groups between post-neonatal and 59 years.

1. Data-rich countries

For data-rich countries modeled in CODEm, we used the covariates listed in Table 1 to inform predictions. New this cycle, the maternal care and immunisation (MCI) covariate was removed in favor of using measures of health access and quality (HAQ) and sociodemographic index (SDI) to predict. In addition, age- and sex-specific summary exposure values (SEV) for child underweight were added to the model to replace the malnutrition proportion covariate used in prior GBD cycles.

Table 1. Covariates. Summary of covariates used in the data-rich pertussis cause of death model

Level	Covariate	Direction
1	Diphtheria-tetanus-pertussis third-dose vaccination coverage (DTP3)	-
	Age- and sex-specific SEV for child underweight	+
	Healthcare Access and Quality (HAQ) Index	-

3	Lag-distributed income (LDI)	-
	Socio-demographic Index (SDI)	-
	Mean years of education per capita	-

2. Natural history model

The pertussis natural history model uses GBD estimates of non-fatal pertussis cases and an intermediate, custom model of pertussis case fatality rate (CFR) to produce estimates in non-data-rich locations where pertussis mortality data are sparse. As described in the non-fatal pertussis modelling text, case notifications informing the pertussis non-fatal model come from the World Health Organization (WHO) Joint Reporting Form (JRF) and historical documentation of pertussis cases and vaccination from the UK. The pertussis CFR data are compiled through systematic reviews of the literature. This systematic review was not updated for GBD 2019.

With the available pertussis CFR input data, we make location- and year-specific estimates using a negative binomial model with the Healthcare Access and Quality (HAQ) Index as a covariate:

$$Y_{ij} = \beta_0 + \beta_1 HAQ_{ij} + u_j + e_{ij} \,,$$

Pertussis log-transformed incidence – modelled independently – is generated from a mixed effects linear regression model predicting pertussis cases as a function of vaccination coverage. Combining these estimates of incidence for every estimated location and year with location-/year-specific estimates of pertussis CFR, pertussis deaths were calculated as:

$$deaths = incidence * CFR \,.$$

This calculation was replicated at the draw level 1000 times in order to produce estimates of total deaths by location and year and associated uncertainty. These draw-level estimates were age- and sex-split using an age-sex distribution based on global-level age- and sex-specific patterns found in the cause of death data, then summarised as the mean of the draws and a 95% uncertainty interval (the 2.5th and 97.5th quantile of all draws).

Tetanus

Flowchart

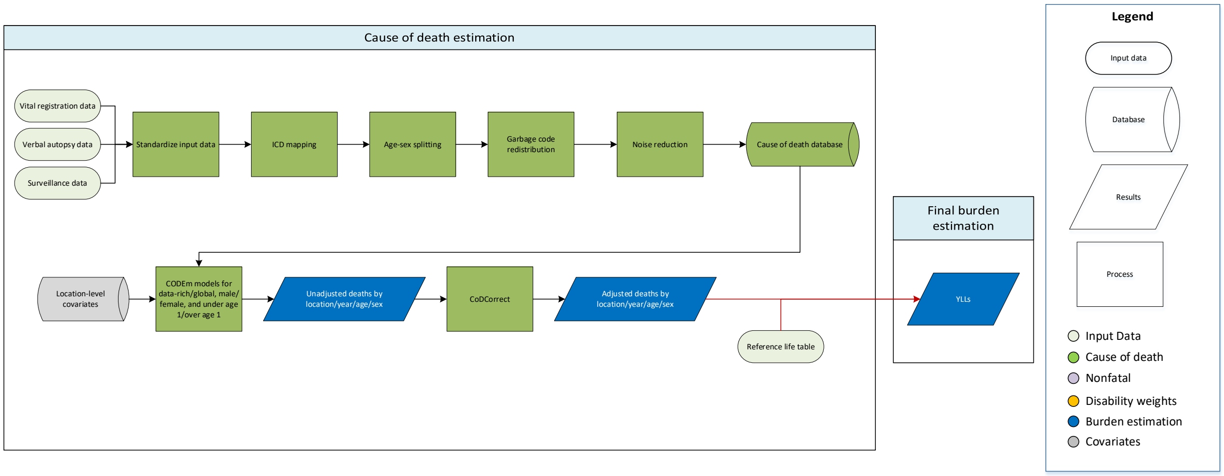

Input data

Tetanus cause of death (COD) data for GBD 2019 included vital registration, verbal autopsy, and surveillance sources from all locations as available. We excluded prepared COD data if they were highly incongruent with other available data from the same location or locations of similar sociodemographic characteristics.

Modelling strategy

We used a Cause of Death Ensemble modelling approach (CODEm) to compute age-, sex-, location-, and year-specific estimates. Given the relative rarity of tetanus mortality, we modelled directly in count-space. These models in count space had lower out-of-sample root mean squared error (RMSE) than rate-space models, and thus were frequently the top models selected in the ensemble.

Separate, sex-specific models were run for neonatal tetanus (under-1-year age groups) and all other tetanus (1 year to 95+ age groups). We also stratified models by vital registration data quality, running both “data-rich” and global models for each age- and sex-specific group. Following model completion, the data-rich and global model outputs were combined to produce a single set of estimates for all locations by sex and age (under-1 and over-1 age groups).

Table 1a lists the covariates used in the data-rich and global under-1 models, and table 1b the covariates in the over-1 model. In both the under-1 and over-1 models, Healthcare Access and Quality (HAQ) Index and Socio-demographic Index (SDI) were used to capture the effect of the maternal care and immunisation (MCI) covariate used in prior GBD cycles.

Table 1a. Covariates. Summary of covariates used in the under-1 tetanus cause of death model

Level	Covariate	Direction
1	Diphtheria-tetanus-pertussis third-dose vaccination coverage (DTP3)	-
	Tetanus toxoid coverage	-
2	In-facility deliveries (proportion)	-
	Skilled birth attendance (proportion)	-
	Healthcare Access and Quality (HAQ) Index	-
3	Lag-distributed income (LDI)	-
	Socio-demographic Index (SDI)	-
	Mean years of education per capita	-

Table 1b. Covariates. Summary of covariates used in the over-1 tetanus cause of death model

Level	Covariate	Direction
1	Diphtheria-tetanus-pertussis third-dose vaccination coverage (DTP3)	-
2	Healthcare Access and Quality (HAQ) Index	-
3	Sanitation access (proportion)	-
	Lag-distributed income (LDI)	-
	Socio-demographic Index (SDI)	-
	Mean years of education per capita	-

Measles

Model flowchart

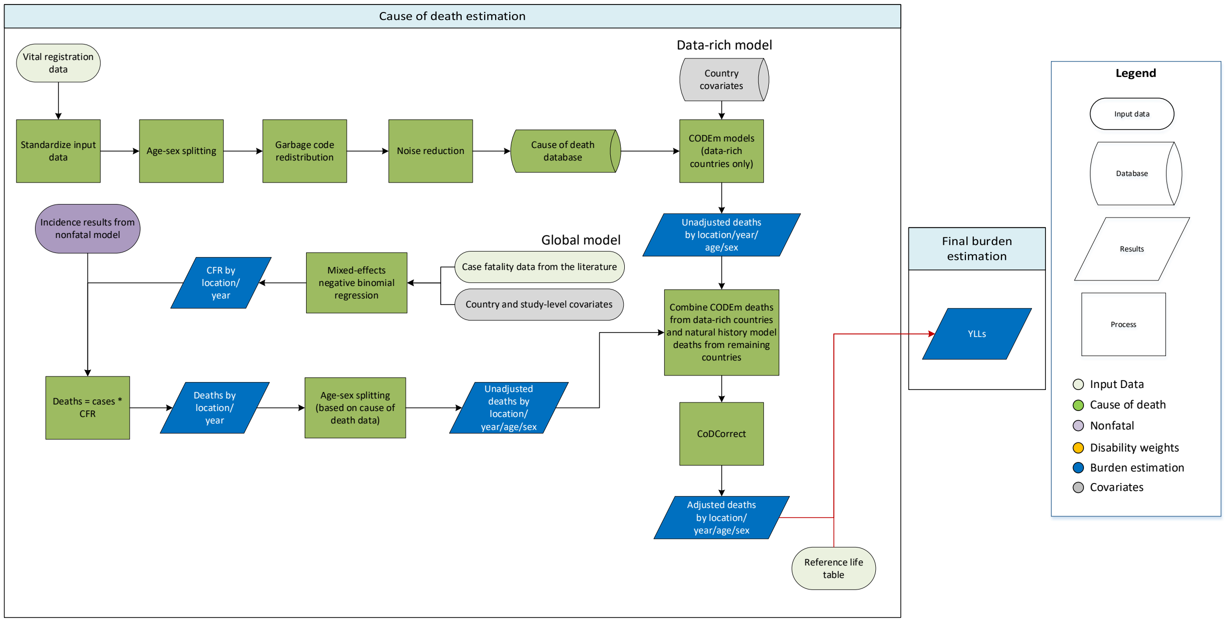

Modelling strategy overview

The GBD 2019 measles mortality estimates were generated in one of two ways depending on the quality of available vital registration data for the country. For countries with well-defined vital registration (ie, "data-rich" countries), we used a Cause of Death Ensemble model (CODEm). For the remaining countries, we leveraged a natural history model approach, drawing from preceding non-fatal case estimates. For all countries, we made estimates for all age groups between post-neonatal and 59 years.

Data-rich countries

For data-rich countries modeled in CODEm, we used the covariates listed in Table 1 to inform predictions. New this cycle, the Healthcare Access and Quality (HAQ) Index and Socio-demographic Index (SDI) covariates were used to capture the effect of the maternal care and immunisation (MCI) covariate used in prior GBD cycles.

Table 1. Covariates. Summary of covariates used in the data-rich measles cause of death model

Level	Covariate	Direction
1	Measles-containing vaccination dose one (MCV1)	-
2	Healthcare Access and Quality (HAQ) Index	-
3	Socio-demographic index (SDI)	-
	Mean years of education per capita	-

Natural history model

A natural history model is used to estimate measles mortality in non-data-rich locations where mortality data are sparse. GBD estimates of non-fatal measles cases are combined with estimates of measles case-fatality rate (CFR) generated by an intermediate, custom CFR model to produce this output. As described in the non-fatal measles modelling methods text, case notifications informing the measles non-fatal model come from the World Health Organization (WHO) Joint Reporting Form (JRF) and additional case notification sources identified by collaborators (eg, Japan and USA subnational measles surveillance data). The measles CFR data are compiled through systematic reviews of the literature, and this search was updated in GBD 2019. This search was conducted in PubMed using the following search string: *((((measles[MeSH Terms] OR measles) AND (mortality[MeSH Terms] OR mortality OR "case fatality rate" OR "case fatality ratio" OR "case fatality"))) AND ("2016"[Date - Publication] : "2019"[Date - Publication]))*.

With the available measles CFR input data, we make location- and year-specific death estimates using a negative binomial model with Socio-demographic Index (SDI) as a country-level covariate, additionally accounting for three indicators (hospital-based or not; outbreak or not; and rural or urban/mixed) as study-level covariates, with country random effects:

$$Y_{ij} = \beta_0 + \beta_1 SDI_{ij} + \beta_2 hospital_{ij} + \beta_3 outbreak_{ij} + \beta_4 rural_{ij} + u_j + e_{ij}$$

where Y_{ij} is the number of deaths (using measles cases as the offset term); β_0 is the fixed-effect intercept; β_1, β_2, β_3, and β_4 are the fixed-effects slopes on the Socio-demographic Index (SDI) and hospital, outbreak, and rurality study-level covariates; u_j is country-level random effects; e_{ij} is the residual; *i* is the year; and *j* is the location. Uncertainty was estimated by taking 1000 iterations of the predictions based on the variance-covariance matrix and uncertainty in country random effects.

Measles log-transformed incidence – modelled independently – is generated from a mixed effects linear regression model predicting measles cases as a function of vaccination coverage (rolling means of MCV1 and MCV2 over the preceding five years, and five-year lagged SIA coverage) given WHO case notification data from countries in the high-income, central Europe/eastern Europe/central Asia, and Latin America and Caribbean super-regions. Combining these estimates of incidence for every estimated location-year with location- and year-specific estimates of measles CFR, measles deaths were calculated as:

$$deaths = incidence * CFR$$

This calculation was replicated at the draw level 1000 times, producing draw-level estimates of total measles deaths for each location and year, which were then split by age and sex using an age-sex distribution based on global-level age- and sex-specific patterns found in the cause of death data. All draw-level estimates were then summarised as the mean of the draws along with a 95% uncertainty interval (the 2.5th and 97.5th quantile of all draws).

Maternal disorders

Flowchart

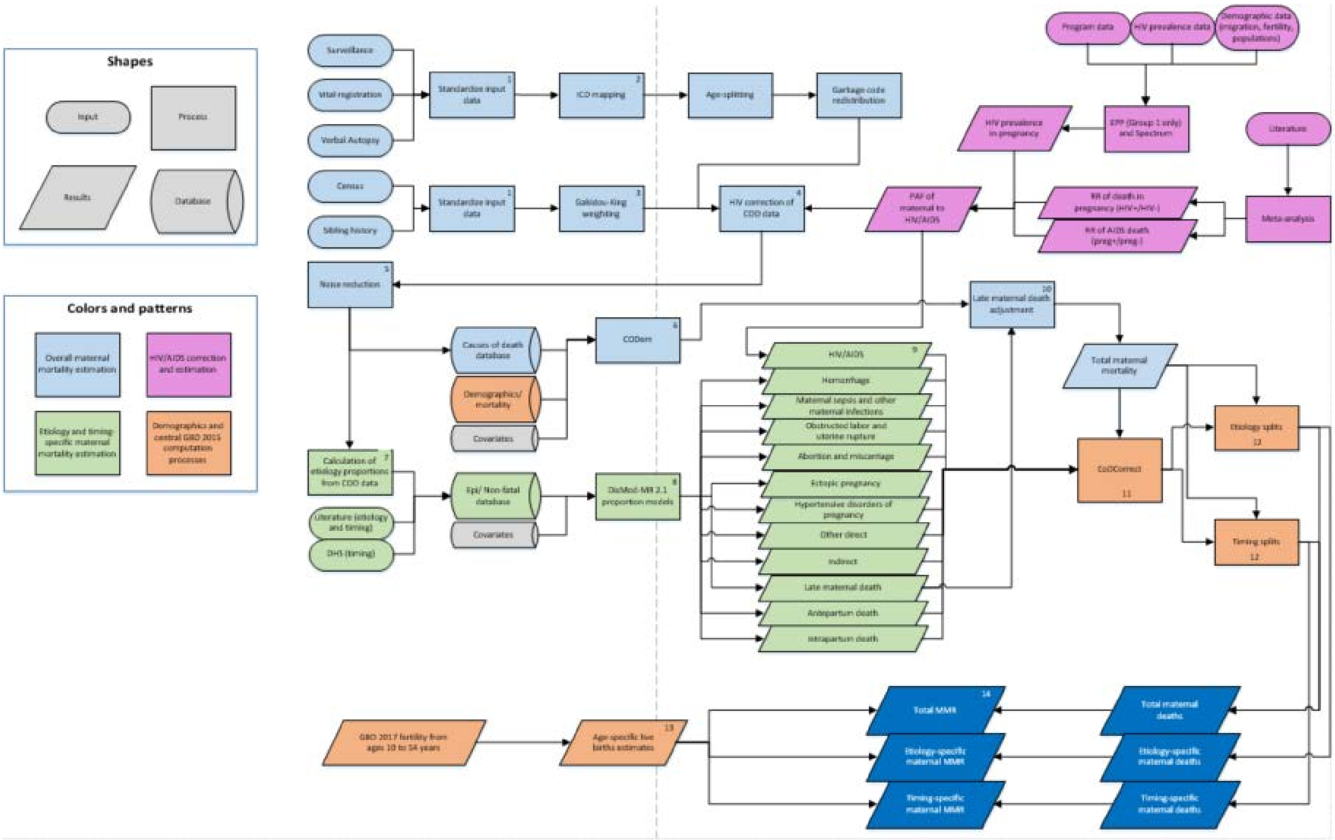

Input data

CODEm models of overall maternal mortality were informed by centrally prepped data stored in the cause of death (COD) database. All data were corrected for incidental HIV deaths by combining estimated HIV prevalence in pregnancy with relative risk (RR) of mortality during pregnancy for HIV-positive women to calculate a population attributable fraction (PAFs) that was then divided between incidental and maternal deaths based on RR of death in HIV-positive women during pregnancy. Incidental HIV deaths were removed from sibling history and census data, while maternal HIV deaths were added to vital registration, verbal autopsy, and surveillance data. This process is described in more detail in the appendix section on HIV/AIDS estimation.

For cause-specific maternal mortality, we used data from the COD database, other data sources and reports from the Global Health Data Exchange, and data from published studies identified through the search below. All data from all geographies were reviewed in CODEm models. Outliers were identified as those data where age patterns or temporal patterns were inconsistent with neighbouring age groups or locations or where sparse data were predicting implausible overall temporal or age patterns for a given location.

Our systematic literature review for maternal disorders is completed annually and encompasses all aspects of maternal disorder burden estimation including overall maternal mortality, cause-specific

maternal mortality, incidence of pregnancy complications by type, relative risk of mortality in pregnancy in HIV-positive versus HIV-negative women, and relative risk of mortality in HIV-positive women who are pregnant versus non-pregnant. We completed this search May 10, 2019, using the following search string:

> (((("Postpartum Hemorrhage" OR "Uterine Hemorrhage") OR (maternal[Title/Abstract] OR pregnan*[Title/Abstract] OR mothers) AND (haemorrhag*[Title/Abstract] OR hemorrhag*[Title/Abstract]) NOT "case report"[All fields]) OR (("induced abortion" OR "Therapeutic abortion" OR "legal Abortion" OR "medical abortion" OR "miscarriage" OR "Abortion, Induced"[Mesh] OR "Abortion, Therapeutic"[Mesh] OR "Abortion, Legal"[Mesh] OR "ectopic Pregnancy") NOT ("case report"[Title/Abstract] OR "birth defect"[Title/Abstract] OR congenital[Title/Abstract])) OR ("obstructed labour" OR "obstructed labor" OR "labour dystocia" OR "labor dystocia" OR dystocia OR "cephalopelvic disproportion" OR "cephalo-pelvic disproportion") OR (("obstetric fistula" OR "vesicovaginal fistula") OR "rectovaginal fistula") OR (("Puerperal Infection"[Mesh] OR "Puerperal Infection" OR ((maternal[Title/Abstract] OR pregnan*[Title/Abstract]) AND (Sepsis OR infection[Title/Abstract]))) NOT "case report") OR ((pre-eclampsia[Title/Abstract] OR preeclampsia[Title/Abstract] OR eclampsia[Title/Abstract] OR Pre-Eclampsia[Mesh] OR Eclampsia[Mesh] OR "Hypertension, Pregnancy-Induced"[Mesh] OR "pregnancy induced hypertension"[Title/Abstract] OR "gestational hypertension"[Title/Abstract] OR "Hypertensive disorders of pregnancy"[Title/Abstract]) NOT ("case report" OR "kidney donor"[Title/Abstract] OR "kidney donors"[Title/Abstract] OR polymorphism*[Title/Abstract] OR endotheli*[Title/Abstract]))) OR((("maternal mortality"[Title/Abstract] OR "maternal death"[Title/Abstract] OR "maternal deaths"[Title/Abstract] OR "MM"[Title/Abstract] OR "confidential enquiry"[Title/Abstract] OR "confidential inquiry"[Title/Abstract] OR ((obstetric[Title/Abstract] OR pregnan*[Title/Abstract]) AND (etiology[Title/Abstract] OR cause[Title/Abstract] OR pattern[Title/Abstract]) AND (death[Title/Abstract] OR mortality[Title/Abstract]))) NOT (fetal[Title/Abstract] OR newborn*[Title/Abstract] OR neonatal[Title/Abstract] OR "case report" [Title/Abstract] OR "case study" [Title/Abstract] OR pathogenesis[Title/Abstract] OR thromboprophylaxis[Title/Abstract])) OR ((("maternal mortality"[Title/Abstract] OR "maternal death"[Title/Abstract] OR "maternal deaths"[Title/Abstract] OR "MMR"[Title/Abstract]) AND ("Afghanistan"[Title/Abstract] OR "Albania"[Title/Abstract] OR "Algeria"[Title/Abstract] OR "Andorra"[Title/Abstract] OR "Angola"[Title/Abstract] OR "Antigua and Barbuda"[Title/Abstract] OR "Argentina"[Title/Abstract] OR "Armenia"[Title/Abstract] OR "Azerbaijan"[Title/Abstract] OR "Bahrain"[Title/Abstract] OR "Bangladesh"[Title/Abstract] OR "Barbados"[Title/Abstract] OR "Belarus"[Title/Abstract] OR "Belize"[Title/Abstract] OR "Benin"[Title/Abstract] OR "Bhutan"[Title/Abstract] OR "Bolivia"[Title/Abstract] OR "Bosnia and Herzegovina"[Title/Abstract] OR "Botswana"[Title/Abstract] OR "Brazil"[Title/Abstract] OR "Brunei"[Title/Abstract] OR "Bulgaria"[Title/Abstract] OR "Burkina Faso"[Title/Abstract] OR "Burundi"[Title/Abstract] OR "Cambodia"[Title/Abstract] OR "Cameroon"[Title/Abstract] OR "Cape Verde"[Title/Abstract] OR "Central African Republic"[Title/Abstract] OR "Chad"[Title/Abstract] OR "China"[Title/Abstract] OR "Colombia"[Title/Abstract] OR "Comoros"[Title/Abstract] OR "Congo"[Title/Abstract] OR "Costa Rica"[Title/Abstract] OR "Croatia"[Title/Abstract] OR "Cuba"[Title/Abstract] OR "Cyprus"[Title/Abstract] OR "Côte d'Ivoire"[Title/Abstract] OR "Democratic Republic of the Congo"[Title/Abstract] OR "Djibouti"[Title/Abstract] OR "Dominica"[Title/Abstract] OR "Dominican Republic"[Title/Abstract] OR "Ecuador"[Title/Abstract] OR "Egypt"[Title/Abstract] OR "El Salvador"[Title/Abstract] OR "Equatorial Guinea"[Title/Abstract] OR "Eritrea"[Title/Abstract] OR "Ethiopia"[Title/Abstract] OR "Federated States of Micronesia"[Title/Abstract] OR "Fiji"[Title/Abstract] OR "Gabon"[Title/Abstract] OR "Georgia"[Title/Abstract] OR "Ghana"[Title/Abstract] OR "Grenada"[Title/Abstract] OR "Guatemala"[Title/Abstract] OR "Guinea"[Title/Abstract] OR "Guinea-Bissau"[Title/Abstract] OR "Guyana"[Title/Abstract] OR "Haiti"[Title/Abstract] OR "Honduras"[Title/Abstract] OR "India"[Title/Abstract] OR "Indonesia"[Title/Abstract] OR "Iran"[Title/Abstract] OR "Iraq"[Title/Abstract] OR "Jamaica"[Title/Abstract] OR "Jordan"[Title/Abstract] OR "Kazakhstan"[Title/Abstract] OR "Kenya"[Title/Abstract] OR "Kiribati"[Title/Abstract] OR "Kuwait"[Title/Abstract] OR "Kyrgyzstan"[Title/Abstract] OR "Laos"[Title/Abstract] OR "Latvia"[Title/Abstract] OR "Lebanon"[Title/Abstract] OR "Lesotho"[Title/Abstract] OR "Liberia"[Title/Abstract] OR "Libya"[Title/Abstract] OR "Lithuania"[Title/Abstract] OR "Macedonia"[Title/Abstract] OR "Madagascar"[Title/Abstract] OR "Malawi"[Title/Abstract] OR "Malaysia"[Title/Abstract] OR "Maldives"[Title/Abstract] OR "Mali"[Title/Abstract] OR "Malta"[Title/Abstract] OR "Marshall Islands"[Title/Abstract] OR "Mauritania"[Title/Abstract] OR "Mauritius"[Title/Abstract] OR "Moldova"[Title/Abstract] OR "Mongolia"[Title/Abstract] OR "Montenegro"[Title/Abstract] OR "Morocco"[Title/Abstract] OR "Mozambique"[Title/Abstract] OR "Myanmar"[Title/Abstract] OR "Namibia"[Title/Abstract] OR

"Nepal"[Title/Abstract] OR "Nicaragua"[Title/Abstract] OR "Niger"[Title/Abstract] OR "Nigeria"[Title/Abstract] OR "North Korea"[Title/Abstract] OR "Oman"[Title/Abstract] OR "Pakistan"[Title/Abstract] OR "Palestine"[Title/Abstract] OR "Panama"[Title/Abstract] OR "Papua New Guinea"[Title/Abstract] OR "Paraguay"[Title/Abstract] OR "Peru"[Title/Abstract] OR "Philippines"[Title/Abstract] OR "Qatar"[Title/Abstract] OR "Romania"[Title/Abstract] OR "Russia"[Title/Abstract] OR "Rwanda"[Title/Abstract] OR "Saint Lucia"[Title/Abstract] OR "Saint Vincent and the Grenadines"[Title/Abstract] OR "Samoa"[Title/Abstract] OR "Saudi Arabia"[Title/Abstract] OR "Senegal"[Title/Abstract] OR "Serbia"[Title/Abstract] OR "Seychelles"[Title/Abstract] OR "Sierra Leone"[Title/Abstract] OR "Singapore"[Title/Abstract] OR "Solomon Islands"[Title/Abstract] OR "Somalia"[Title/Abstract] OR "South Africa"[Title/Abstract] OR "South Sudan"[Title/Abstract] OR "Sri Lanka"[Title/Abstract] OR "Sudan"[Title/Abstract] OR "Suriname"[Title/Abstract] OR "Swaziland"[Title/Abstract] OR "Syria"[Title/Abstract] OR "São Tomé and Principe"[Title/Abstract] OR "Taiwan"[Title/Abstract] OR "Tajikistan"[Title/Abstract] OR "Tanzania"[Title/Abstract] OR "Thailand"[Title/Abstract] OR "The Bahamas"[Title/Abstract] OR "The Gambia"[Title/Abstract] OR "Timor-Leste"[Title/Abstract] OR "Togo"[Title/Abstract] OR "Tonga"[Title/Abstract] OR "Trinidad and Tobago"[Title/Abstract] OR "Tunisia"[Title/Abstract] OR "Turkmenistan"[Title/Abstract] OR "Uganda"[Title/Abstract] OR "Ukraine"[Title/Abstract] OR "United Arab Emirates"[Title/Abstract] OR "Uruguay"[Title/Abstract] OR "Uzbekistan"[Title/Abstract] OR "Vanuatu"[Title/Abstract] OR "Venezuela"[Title/Abstract] OR "Vietnam"[Title/Abstract] OR "Yemen"[Title/Abstract] OR "Zambia"[Title/Abstract] OR "Zimbabwe"[Title/Abstract])) NOT ("demographic and health survey"[Title/Abstract] OR "demographic and health surveys "[Title/Abstract] OR DHS[Title/Abstract] OR "reproductive health survey"[Title/Abstract] OR "reproductive health surveys"[Title/Abstract] OR RHS[Title/Abstract])) OR ((HIV[Title/Abstract] OR "Acquired Immunodeficiency Syndrome"[Title/Abstract] OR AIDS[Title/Abstract]) AND (pregnan*[Title/Abstract] OR "postpartum"[Title/Abstract] OR "post partum"[Title/Abstract]) AND ("mortality"[Title/Abstract] OR "death"[Title/Abstract]) NOT "case report")) AND (2017/07/01[PDat] : 3000[PDat]) NOT (animals[MeSH] NOT humans[MeSH]))

A total of 12 964 literature sources were reviewed for their title and abstract. Of the 272 sources selected for full text review, 81 were extracted to inform maternal disorder models (fatal and non-fatal). There were no new sources extracted for maternal deaths aggravated by HIV. All cause-specific maternal mortality data were extracted as maternal mortality ratio (MMR; cause-specific deaths per live birth). All cause-specific COD data, along with any sources that reported cause-specific maternal deaths in cause fraction or population rate terms, were converted to MMR using all-cause mortality, population, and age-specific fertility results estimated in GBD 2019.

One exception was late maternal death, where only raw, unprocessed COD data were included from the COD database, and only for the subset of locations where the proportion of late maternal deaths coded in VR exceeded the lowest published rate from a comprehensive study.[1] Our assumption is that any location that has never reported a late maternal death in its VR does not capture any late maternal deaths. These data were supplemented with late maternal death data, all of which was extracted and prepped as proportion of the total. for the subset of locations where they were reliably coded in raw VR. All cause-specific MMR and proportion (late only) data were uploaded to the non-fatal database.

Modelling strategy

Overall maternal mortality

Overall maternal mortality was estimated with CODEm. Covariates included in this model, their level, and directionality are show in the table below:

Table 1: Covariates used in CODEm models of overall maternal mortality

Level	Covariate	Direction
Level 1	Age-specific fertility rate	+
	Total fertility rate (log-transformed)	+
	Maternal education (years per capita)	–
	In-facility delivery (proportion)	–
	Skilled birth attendance (proportion)	–
	Neonatal mortality ratio (log-transformed)	+
	Age-specific HIV mortality in females 10-54 (log-transformed)	+
Level 2	Antenatal care 1-visit coverage (proportion)	-
	Antenatal care 4-visits coverage (proportion)	-
	Age-standardised wasting (weight-for-height) summary exposure value (SEV)	+
	Age-standardised stunting (height-for-age) SEV	+
	Healthcare Access and Quality Index	-
	Age- and sex-specific SEV for high body-mass index (BMI)	+
	Age- and sex-specific SEV for high blood pressure (SBP)	+
	Underweight women of reproductive age	+
Level 3	Socio-demographic Index	-
	Mortality shock (cumulative rate in last 10 years)	+
	LDI (log-transformed)	-
	Hospital beds (per 1,000 population)	–

Cause-specific maternal mortality

We used spatiotemporal Gaussian process regression (ST-GPR) to estimate MMRs for each of the eight maternal subcauses. This modeling strategy requires data to be in standard GBD age groups. To achieve this, we used the global age pattern of the COD data for each cause and applied it to all data that were not in the standard GBD age groups. ST-GPR also requires variance for each datapoint. In order to compute variance, we ran a Lowess regression on the data by year and used the variance of the residuals resulting from the difference between the data and the predicted values.

The first step in the past has been a mixed-effects ordinary least squares regression of the quantity of interest and a specified set of location-level covariates. For GBD 2019 we revised this first step to instead be informed by an ensemble of regressions where weighting of each component model was based on out-of-sample coverage prediction performance. This approach allowed us to test a larger number of covariates and also specify the directionality of relationships between location-level covariates and the outcome of interest. Country covariates were specific for each subcause model, as shown in the table below:

Table 2: Covariates used in generation of ensemble stage 1 predictions of cause-specific maternal mortality ST-GPR models

Maternal subcause	Country-level covariates	Direction
Maternal haemorrhage	In-facility delivery (proportion)<br>Skilled birth attendance (proportion)<br>Age- and sex-specific SEV for unsafe sanitation<br>Neonatal mortality ratio (log-transformed)<br>Maternal education<br>Healthcare Access and Quality Index	-<br>-<br>+<br>+<br>-<br>-
Maternal hypertensive disorders	Age- and sex-specific SEV for fasting plasma glucose (FPG)<br>Age- and sex-specific SEV for high body-mass index (BMI)<br>Age- and sex-specific SEV for high blood pressure (SBP)<br>Neonatal mortality ratio (log-transformed)<br>Hospital beds (per 1000 population)<br>Antenatal care 1-visit coverage (proportion)<br>Antenatal care 4-visits coverage (proportion)<br>Healthcare Access and Quality Index	+<br>+<br>+<br>+<br>-<br>-<br>-<br>-
Obstructed labour and uterine rupture	In-facility delivery (proportion)<br>Skilled birth attendance (proportion)<br>Underweight women of reproductive age<br>Neonatal mortality ratio (log-transformed)<br>Hospital beds (per 1000 population)<br>Age-standardised wasting (weight-for-height) SEV<br>Age-standardised stunting (height-for-age) SEV	-<br>-<br>+<br>+<br>-<br>+<br>+
Abortion and miscarriage	Abortion legality<br>Antenatal care 1-visit coverage (proportion)<br>Antenatal care 4-visits coverage (proportion)<br>Hospital beds (per 1,000 population)<br>Maternal education<br>Healthcare Access and Quality Index	-<br>-<br>-<br>-<br>-<br>-
Ectopic pregnancy	Abortion legality<br>Pelvic inflammatory disease age-standardised prevalence<br>Antenatal care 1-visit coverage (proportion)<br>Antenatal care 4-visits coverage (proportion)<br>Hospital beds (per 1,000 population)<br>Maternal education<br>Healthcare Access and Quality Index	-<br>+<br>-<br>-<br>-<br>-<br>-
Maternal sepsis and other maternal infections	In-facility delivery (proportion)<br>Skilled birth attendance (proportion)<br>Age- and sex-specific SEV for unsafe sanitation<br>Age- and sex-specific SEV for fasting plasma glucose (FPG)<br>Antenatal care 1-visit coverage (proportion)<br>Antenatal care 4-visits coverage (proportion)<br>LDI (log-transformed)<br>Healthcare Access and Quality Index	-<br>-<br>+<br>+<br>-<br>-<br>-<br>-
Other maternal deaths	In-facility delivery (proportion)<br>Skilled birth attendance (proportion)<br>Antenatal care 1-visit coverage (proportion)<br>Antenatal care 4-visits coverage (proportion)	-<br>-<br>-<br>-

	LDI (log-transformed)<br>Age- and sex-specific SEV for high body-mass index (BMI)<br>Maternal education<br>Healthcare Access and Quality Index	-<br>+<br>-<br>-
Indirect maternal deaths	In-facility delivery (proportion)<br>Skilled birth attendance (proportion)<br>Antenatal care 1-visit coverage (proportion)<br>Antenatal care 4-visits coverage (proportion)<br>LDI (log-transformed)<br>Age- and sex-specific SEV for high body-mass index (BMI)<br>Maternal education<br>Healthcare Access and Quality Index	-<br>-<br>-<br>-<br>-<br>+<br>-<br>-

Late maternal death and model processing

Aetiology-specific estimates were derived by scaling the results from the ST-GPR subcause-specific models scaled in relation to each other to equal one and then multiplying them by the total maternal deaths, corrected for late maternal deaths, for that age group, location, and year. A single parameter proportion model was run in Dismod-MR 2.1 for late maternal deaths using the data described above. The proportions coming for the VR data sources were taken before any of the central data processing. We used the Healthcare Access and Quality Index as a country-level covariate for the model.

Neonatal disorders

Flowchart

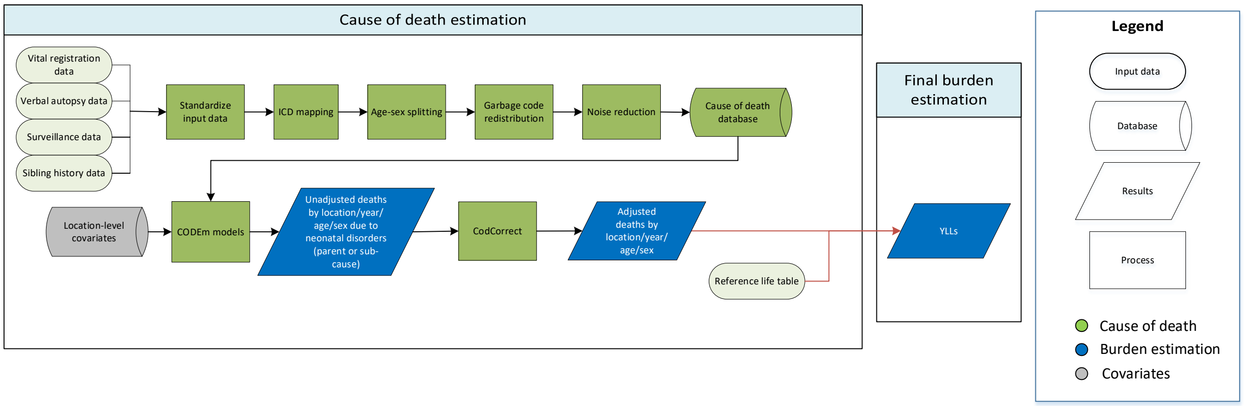

Input Data and Methodological Summary for Neonatal Disorders

Mortality for five causes are modeled within "neonatal disorders": neonatal preterm birth complications, neonatal encephalopathy due to birth asphyxia and trauma, neonatal sepsis and other neonatal infections, hemolytic disease and other neonatal jaundice, and other neonatal disorders. An overall neonatal disorders "parent" envelope is also estimated, to which all neonatal causes are squeezed.

Input data

Vital registration and surveillance were the majority of data sources used for GBD 2019 to estimate number of deaths from each condition. In Indian states, only verbal autopsy were used to inform estimates. Only deaths among males and females under age 5 were modelled, in four separate age groups: early neonatal period, late neonatal period, post-neonatal period, and 1-4 years. Data points were selected as outliers if they were implausibly high, low, or significantly conflicted with established age or temporal patterns. A significant new data source in GBD 2019 is Child Health and Mortality Prevention Surveillance (CHAMPS) in Bangladesh, Kenya, Mozambique, South Africa and Mali.

Modeling strategy

The standard CODEm modelling approach was used to model each of the neonatal conditions. Varying levels of data quality and coding issues may have affected our results. Validation studies suggest that verbal autopsy methods tend to be less accurate for cause of death ascertainment in the neonatal age groups.[1-4] Thus, for GBD 2019, except for the Indian states, the majority of verbal autopsy data were excluded. All neonatal causes used the following pool of covariates in covariate selection:

Table 1. Covariates used in neonatal disorders mortality modelling

Level	Covariate	Direction
1	Maternal care and immunization	-
	Age-standardized SEV for Ambient particulate matter	+
	Age-standardized SEV for Household air pollution	+
	Age-standardized SEV for Short gestation	+
	Age-standardized SEV for Low birth weight	+
	Age-standardized SEV for Smoking	+

2	Proportion of the population with at least 12 years of education, maternal	-
	Proportion of the population with at least 6 years of education, maternal	-
	Live Births 35+ (proportion)	+
	Socio-demographic Index	-
	Healthcare access and quality index	-
3	Antenatal Care (1 visit) Coverage (proportion)	-
	Antenatal Care (4 visits) Coverage (proportion)	-
	In-Facility Delivery (proportion)	-
	LDI (I$ per capita)	-
	Skilled Birth Attendance (proportion)	-
	Total Fertility Rate	+

References

1. Anker M, Black RE, Coldham C, *et al.* A Standard Verbal Autopsy Method for Investigating Causes of Death in Infants and Children. Geneva, Switzerland: World Health Organization Department of Communicable Disease Surveillance and Response; The Johns Hopkins School of Hygiene and Public Health; The London School of Hygiene and Tropical Medicine, 1999.

2. Kalter HD, Gray RH, Black RE, Gultiano SA. Validation of postmortem interviews to ascertain selected causes of death in children. *Int J Epidemiol* 1990; **19**: 380–6.

3. Quigley MA, Armstrong Schellenberg JR, Snow RW. Algorithms for verbal autopsies: a validation study in Kenyan children. *Bull World Health Organ* 1996; **74**: 147–54.

4. Snow RW, Armstrong JR, Forster D, *et al.* Childhood deaths in Africa: uses and limitations of verbal autopsies. *The Lancet* 1992; **340**: 351–5.

Cancers

Input data and methodological summary for all cancers except for non-melanoma skin cancer

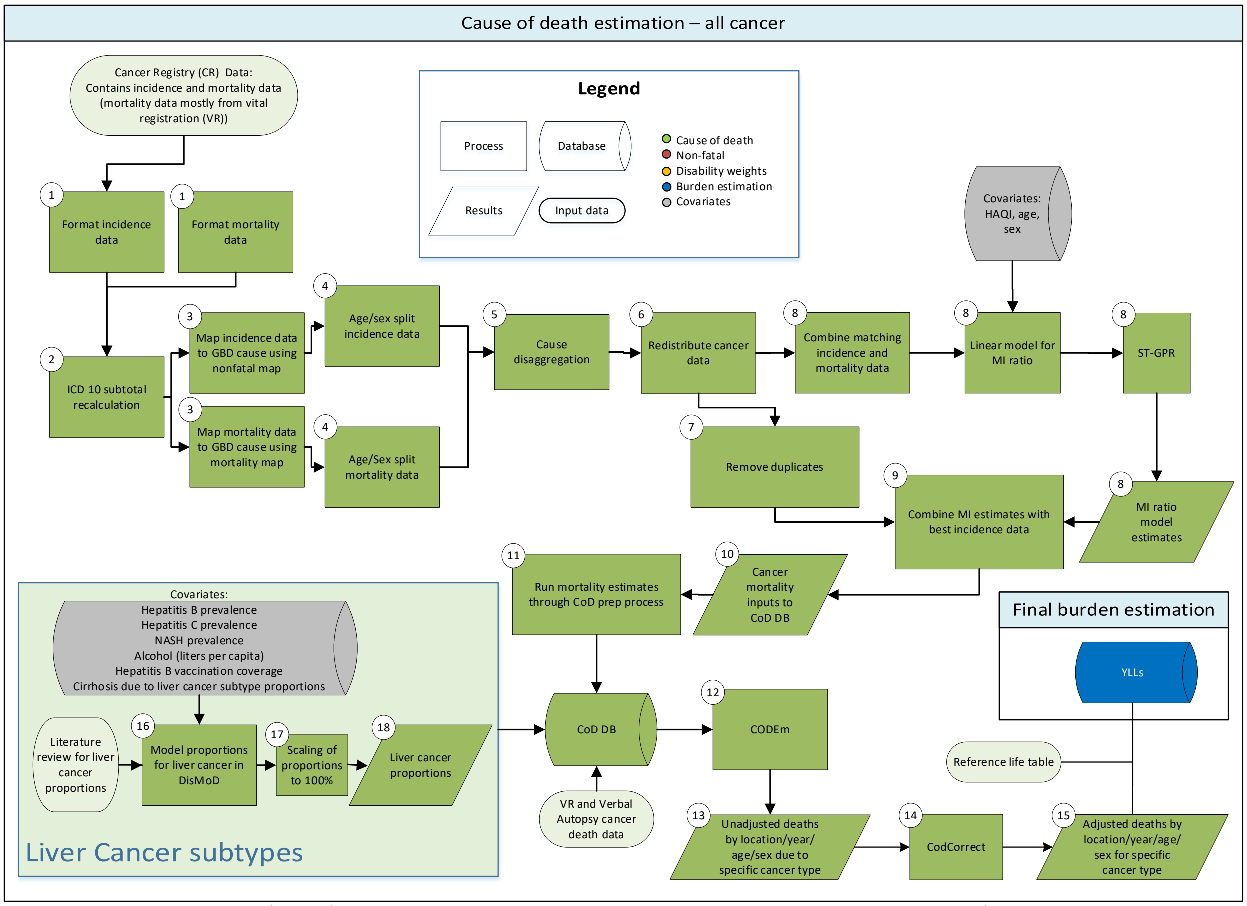

Abbreviations: ICD: International classification of diseases; DB: database, ST-GPR: Space-time smoothing, Gaussian process regression, COD: Causes of death

Data

The cause of death (COD) database contains multiple sources of cancer mortality data. These sources include vital registration, verbal autopsy, and cancer registry data. The cancer registry mortality estimates that are uploaded into the COD database stem from cancer registry incidence data that have been transformed to mortality estimates through the use of mortality-to-incidence ratios (MIR).

Data-seeking processes

Cancer mortality data in the cause of death database other than cancer registry data

Sources for cancer mortality data other than cancer registry data are described in the COD database description (Appendix Section 2.2).

Cancer registry data

Cancer registry data were used from publicly available sources or provided by collaborators. We used all data from GBD 2017 and added registry data from Argentina, Australia, Austria, Bermuda, Canada, Chile, China, Colombia, Germany, Netherlands, Switzerland, United Kingdom, Uruguay, and Yemen.

Inclusion and exclusion criteria

Only population-based cancer registries were included, and only those that included all cancers (no specialty registries), data for all age groups (except for paediatric cancer registries), and data for both sexes. Pathology-based cancer registries were included if they had a defined population. Hospital-based cancer registries were excluded.

Cancer registry data were excluded from either the final incidence data input or the MI model input if a more detailed source (eg, providing more detailed age or diagnostic groups) was available for the same population. Preference was given to registries with national coverage over those with only local coverage, except those from countries where the GBD study provides subnational estimates.

Data were excluded if the coverage population was unknown.

Bias of categories of input data

Cancer registry data can be biased in multiple ways. A high proportion of ill-defined cancer cases in the registry data requires redistribution of these cases to other cancers, which introduces a potential for bias. Changes between coding systems can lead to artificial differences in disease estimates; however, we adjust for this bias by mapping the different coding systems to the GBD causes. Underreporting of cancers that require advanced diagnostic techniques (eg, leukaemia, brain, pancreatic, and liver cancer) can be an issue in cancer registries from low-income countries. On the other hand, misclassification of metastatic sites as primary cancer can lead to overestimation of cancer sites that are common sites for metastases, like the brain or liver. Since many cancer registries are located in urban areas, the representativeness of the registry for the general population can also be problematic. The accuracy of mortality data reported in cancer registries usually depends on the quality of the vital registration system. If the vital registration system is incomplete or of poor quality, the mortality-to-incidence ratio can be biased to lower ratios.

Data for liver cancer aetiology splits

To find the proportion of liver cancer cases due to the five aetiology groups included in GBD (1. Liver cancer due to hepatitis B, 2. Liver cancer due to hepatitis C, 3. Liver cancer due to alcohol, 4. Liver cancer due to non-alcoholic steatohepatitis (NASH), 5. Liver cancer due to other causes), a systematic literature search was performed in PubMed on 10/24/2016 using the following search string: “("liver neoplasms"[All Fields] OR "HCC"[All Fields] OR "liver cancer"[All Fields] OR "Carcinoma, Hepatocellular"[Mesh]) AND (("hepatitis B"[All Fields] OR "Hepatitis B"[Mesh] OR "Hepatitis B virus"[Mesh] OR "Hepatitis B Antibodies"[Mesh] OR "Hepatitis B Antigens"[Mesh]) OR ("hepatitis C"[All Fields] OR "Hepatitis C"[Mesh] OR "hepatitis C antibodies"[MESH] OR "Hepatitis C Antigens"[Mesh] OR "Hepacivirus"[Mesh]) OR ("alcohol"[All Fields] OR "Alcohol Drinking"[Mesh] OR "Alcohol-Related Disorders"[Mesh] OR "Alcoholism"[Mesh] OR "Alcohol-Induced Disorders"[Mesh])) NOT (animals[MeSH] NOT humans[MeSH])”. Also, studies not found through this search but included in the meta-analysis by de Martel and colleagues were included.[10] We also included the study by Hong and colleagues after the authors provided us with additional data on the overlap in risk factors.[11]

Studies were included if the study population was representative of liver cancer for the respective location. For each study, the proportions of liver cancer due to the five specific risk factors were calculated. Cases were considered to be due to NASH when the manuscript explicitly listed the aetiology to be NASH or non-alcoholic fatty liver disease (NAFLD). Cases where the aetiology was listed as “cryptogenic”, “idiopathic”, or “unknown” were included within the “other causes” category. In

manuscripts where the aetiology for a case was not known but major categories could not be ruled out (for example, the study tested for hepatitis B and C, but did not assess alcohol use), these cases were excluded from the numerator of the study (in other words, did not contribute to the proportion of any aetiology). Remaining risk factors were included under a combined “other” group (for example, haemochromatosis, autoimmune hepatitis, Wilson’s disease, etc.). If multiple risk factors were reported for an individual patient, these were apportioned proportionally to the individual risk factors. These estimated proportions are then used to split the overall liver cancer estimates into estimates for their respective aetiologies.

Methods

Steps of analysis and data transformation processes

Cancer registry data went through multiple processing steps before integration with the COD database. First, the original data were transformed into standardised files, which included standardisation of format, categorisation, and registry names (#1 in flowchart).

Second, some cancer registries report individual codes as well as aggregated totals (eg, C18, C19, and C20 are reported individually, but the aggregated group of C18-C20 [colorectal cancer] is also reported in the registry data). The data-processing step “subtotal recalculation” (#2 in flowchart) verifies these totals and subtracts the values of any individual codes from the aggregates.

In the third step (#3 in the flowchart), cancer registry incidence data and cancer registry mortality data are mapped to GBD causes. A different map is used for incidence data and for mortality data because of the assumption that there are no deaths for certain cancers. One example is basal-cell carcinoma of the skin. In the cancer registry incidence data, basal-cell carcinoma is mapped to “non-melanoma skin cancer (basal-cell carcinoma)”. However, if basal-cell skin cancer is recorded in the cancer registry mortality data, the deaths are instead mapped to “non-melanoma skin cancer (squamous-cell carcinoma)” under the assumption that they were indeed squamous-cell skin cancers that had been misclassified as basal-cell skin cancers. Other examples are benign or in situ neoplasms. Benign or in situ neoplasms found in the cancer registry incidence dataset were simply dropped from that dataset. The same neoplasms reported in a cancer registry mortality dataset were mapped to the respective invasive cancer (eg, melanoma in situ in the cancer registry incidence dataset was dropped from the dataset; melanoma in situ in the cancer registry mortality dataset was mapped to melanoma).

In the fourth data-processing step (#4 in the flowchart) cancer registry data were standardised to the GBD age groups. Age-specific incidence rates were generated using all datasets that include microdata, and datasets that report age groups up to 95+ years of age, while age-specific mortality rates were generated from the CoD data through a method described in Appendix section 2.5. Age-specific proportions were then generated by applying the age-specific rates to a given registry population that required age-splitting to produce the expected number of cases/deaths for that registry by age. The expected number of cases/deaths for each sex, age, and cancer were then normalised to 1, creating final, age-specific proportions. These proportions were then applied to the total number of cases/deaths by sex and cancer to get the age-specific number of cases/deaths.

In the rare case that the cancer registry only contained data for both sexes combined, the now-age-specific cases/deaths were split and reassigned to separate sexes using the same weights that are used for the age-splitting process. Starting from the expected number of deaths, proportions were generated by sex for each age (eg, if for ages 15 to 19 years old there are six expected deaths for males and four expected deaths for females, then 60% of the combined-sex deaths for ages 15-19 years would be assigned to males and the remaining 40% would be assigned to females).

In the fifth step (#5 in the flowchart) data for cause entries that are aggregates of GBD causes were redistributed. Examples of these aggregated causes include some registries reporting ICD10 codes C00-C14 together as, "lip, oral cavity, and pharyngeal cancer." These groups were broken down into sub-causes that could be mapped to single GBD causes. In this example, those include lip and oral cavity cancer (C00-C08), nasopharyngeal cancer (C11), cancer of other parts of the pharynx (C09-C10, C12-C13), and "Malignant neoplasm of other and ill-defined sites in the lip, oral cavity, and pharynx" (C14). To redistribute the data, weights were created using the same "rate-applied-to-population" method employed in age-sex splitting (see step four above). For the undefined code (C14 in the example) an "average all cancer" weight was used, which was generated by adding all cases from SEER/NORDCAN/CI5 and dividing the total by the combined population. Then, proportions were generated by sub-cause for each aggregate cause as in the sex-splitting example above (see step four). The total number of cases from the aggregated group (C00-C14) was then recalculated for each subgroup and the undefined code (C14). C14 was then redistributed as a "garbage code" in step six. Distinct proportions were used for C44 (non-melanoma skin cancer) and C46 (Kaposi's sarcoma). Non-melanoma skin cancer processing is described under section "Input data and methodological summary for non-melanoma skin cancer (squamous-cell carcinoma)." C46 entries were redistributed as "other cancer" and HIV using proportions described in Appendix Section 2.

In the sixth step (#6 in the flowchart) unspecified codes ("garbage codes") were redistributed. Redistribution of cancer registry incidence and mortality data mirrored the process of the redistribution used in the cause of death database (Appendix Section 2.7).

In the seventh step (#7 in the flowchart) duplicate or redundant sources were removed from the processed cancer registry dataset. Duplicate sources were present if, for example, the cancer registry was part of the CI5 database but we also had data from the registry directly. Redundancies occurred and were removed as described in "Inclusion and Exclusion Criteria," where more detailed data were available, or when national registry data could replace regionally representative data. From here, two parallel selection processes were run to generate input data for the MI models and to generate incidence for final mortality estimation. When creating the final incidence input, higher priority was given to registry data from the most standardised source; whereas for the MI model input, only sources that reported both incidence and mortality were used.

In the eighth step (#8 in the flowchart) the processed incidence and mortality data from cancer registries were matched by cancer, age, sex, year, and location to generate MI ratios. These MI ratios were used as input for a three-step modelling approach using ST-GPR, with HAQ Index as a covariate in the linear step mixed effects model using a logit link function. Predictions were made without the random effects. The ST-GPR model has three main hyper-parameters that control for smoothing across time, age, and geography, which were adjusted for GBD 2019. The time adjustment parameter lambda

(λ) aims to borrow strength from neighbouring time points (ie, the exposure in this year is highly correlated with exposure in the previous year but less so further back in time). Lambda was lowered from 2 to 0.05, reducing the weight of more distant years. The age adjustment parameter omega (ω) borrows strength from data in neighbouring age groups and was set to 0.5 (unchanged). The space adjustment parameter zeta (ξ) aims to borrow strength across the hierarchy of geographical locations.[12] Zeta was lowered from 0.95 to 0.01, reducing the weight of more distant geographical data. For the remaining parameters in the Gaussian process regression, we lowered the amplitude from 2 to 1 (reducing fluctuation from the mean function) and reduced the scale value from 15 to 10 (reducing the time distance over which points are correlated). These model specification changes generally led to less smoothing of the data compared to GBD 2017 models.

Data-cleaning steps were similar as for GBD 2017. For each cancer, MI ratios from locations in HAQ quintiles 1-4 were dropped if they were below the median of MI ratios from locations in HAQ quintile 5. We also dropped MI ratios from locations in HAQ quintiles 1-4 if the MI ratios were above the third quartile + 1.5 * IQR (inter-quartile range). We dropped all MIR that were based on less than 15 (this was 25 in 2017) cases to avoid noise due to small numbers, except for mesothelioma and acute myeloid leukaemia, where we dropped MIR that were based on less than ten cases because of lower data availability for these two cancers. We also aggregated incidence and mortality to the youngest five-year age bin where SEER reported at least 50 cases from 1990 to 2015, to avoid unstable MIR predictions in young age groups on too few datapoints. The MIR in the minimum age-bin was used to backfill the MIR down to the lowest age group estimated for that cancer.

Since MI ratios can be above 1, especially in older age groups and cancers with low cure rates, we used the 95th percentile (by age group) of the cleaned dataset (detailed above) to cap the MIR input data. This "upper cap" was used to allow MIR over 1 but to constrain the MIR to a maximum level. To run the logit model, the input data were divided by the upper caps to get data from 0 to 1. Model predictions from ST-GPR were then rescaled back by multiplying them by the upper caps.
To constrain the MIRs at the lower end, we used the fifth percentile of the cancer and age-specific cleaned MIR input data to replace all model predictions with this lower cap.

Final MI ratios were matched with the cancer registry incidence dataset in the ninth step (#9 in the flowchart) to generate mortality estimates (Incidence * Mortality/Incidence = Mortality) (#10 in the flowchart). These mortality estimates are then smoothed by a Bayesian noise-reduction algorithm (to deal with problems with zero counts, as also applied to the VR and VA data) and uploaded into the COD database (#11 in the flowchart). Cancer-specific mortality modelling then followed the general CODEm process.

Liver cancer aetiology split models

The proportion data found through the systematic literature review were used as input for five separate DisMod-MR 2.1 models to determine the proportion of liver cancers due to the five subgroups for all locations, both sexes, all years, and all age groups (step #16 in the flowchart). For GBD 2019 we used MR-BRT to split sex-combined input data into sex-specific proportion data. For liver cancer due to hepatitis C and hepatitis B, a prior value of 0 was set between age 0 and 0.01. For liver cancer due to alcohol, a prior value of 0 was set for ages 0 to 5 years. For liver cancer due to hepatitis C, hepatitis C (IgG) seroprevalence was used as a covariate, forcing a positive relationship between the hepatitis C

seroprevalence covariate and the outcome of liver cancer due to hepatitis C proportion. For liver cancer due to hepatitis B, seroprevalence of HBsAg was used as a covariate as well as the population coverage of three-dose Hepatitis B vaccination, forcing a negative relationship between vaccination and the outcome of liver cancer due to hepatitis B proportion. For liver cancer due to alcohol, alcohol (litres per capita) was used as a covariate as well as a covariate for proportion of alcohol abstainers, forcing a negative relationship between the proportion of alcohol abstainers and the outcome of liver cancer due to alcohol proportion. For liver cancer due to NASH, NASH/NAFLD prevalence was used as a covariate as well as a covariate for obesity prevalence and mean body-mass index (BMI), forcing a positive relationship between these covariates and the outcome of liver cancer due to NASH proportion. All covariates used were modelled independently. To ensure consistency between cirrhosis and liver cancer estimates and to take advantage of the data for the respective other related cause (eg, liver cancer due to hepatitis C and the related cause cirrhosis due to hepatitis C), we generated covariates from the liver cancer proportion models that were subsequently used in separate cirrhosis aetiology proportion models. We then created covariates from the cirrhosis aetiology proportion models and used those in final liver cancer aetiology models.

Since the proportion models are run independently of each other, the final proportion models were scaled to sum to 100% within each age, sex, year, and location, by dividing each proportion by the sum of the five (step # 17). For the liver cancer subtype mortality estimates, we multiplied the parent cause "liver cancer" by the corresponding scaled proportions (step # 18). Single cause estimates were adjusted to fit into the separately modelled all-cause mortality envelope in the GBD-wide CoDCorrect process.

Results

Interpretation of results

Cancer mortality estimates for GBD 2019 can differ from the GBD 2017 results for multiple reasons. Updated cancer mortality data were added from vital registration system data, verbal autopsy studies, and cancer registry incidence data. Previously some deaths mapped to liver cancer contained deaths from liver metastases rather than primary liver cancer; for GBD 2019, these deaths were instead mapped as garbage codes and redistributed. The mortality-to-incidence ratio estimation was updated with lower case inclusion criteria and different model hyperparameters compared to GBD 2017, leading to more training data and less smoothing across time and geography. Covariates used in CODEm models were updated for GBD 2019. This included removing or replacing covariates that had been updated by other GBD teams (most of the dietary covariates), assigning a direction of association prior to all covariates (previously covariates such as income and Socio-demographic Index had been allowed to have agnostic direction priors), and changing the minimum age ranges for which the models estimated mortality. Compared to GBD 2017, large differences in the incidence and prevalence estimates for the benign and in-situ neoplasms is due to changes in how the clinical informatics data are processed for these causes. These data are now adjusted for HAQ Index and corrected for outpatient encounters, which should capture significantly more of these cases than before (since that relied on hospital admissions).

The other group producing country-level cancer mortality estimates is the International Agency for Research on Cancer (IARC) with their GLOBOCAN database. Significantly different methods between the GBD study and GLOBOCAN can lead to differences in results. Whereas estimates in GLOBOCAN are based on the assumption that there are "In theory, […] as many methods as countries,"[13] the cancer

estimation process for the GBD study follows a coherent, well-documented method for all cancers, which allows cross-validation of models as well as determination of uncertainty. Another major difference is the ability in the GBD study to adjust single cause estimates to the all-cause mortality, which is being determined independently. This also allows us to adjust individual causes of death to the all-cause mortality envelope, which permits us to correct for the underdiagnosis of cancer in countries with inadequate diagnostic resources. Redistribution of a fraction of undefined causes of death to certain cancers is another methodological advantage the GBD study has over GLOBOCAN, and estimates for cancer mortality can therefore differ substantially in countries with a large proportion of undefined causes of deaths in their vital registration data or a large proportion of undefined cancer cases in their cancer registry data.

Limitations

There are certain limitations to consider when interpreting the GBD mortality cancer estimates. First, even though every effort is made to include the most recently available data for each country, data-seeking resources are not limitless and new data cannot always be accessed as soon as they are made available. It is therefore possible that the GBD study does not include all available data sources for cancer incidence or cancer mortality. Second, different redistribution methods can potentially change the cancer estimates substantially if the data sources used for the estimated location contain a large number of undefined causes; however, neglecting to account for these undefined deaths would likely introduce an even greater bias in the disease estimates. Third, using mortality-to-incidence ratios to transform cancer registry incidence data to mortality estimates requires accurate MIR. For GBD 2019 we have made further changes to the MIR estimation, but the method remains sensitive to underdiagnosis of cancer cases or under-ascertainment of cancer deaths. However, given that the majority of data used for the cancer mortality estimation come from vital registration data and not cancer registry data, this is not a major limitation.

Non-melanoma skin cancer (squamous-cell carcinoma)

Data

Data-seeking processes

Since squamous-cell carcinomas are only very infrequently recorded by cancer registries, only vital registration system data were used as input for the squamous-cell carcinoma mortality modelling.

Inclusion and exclusion criteria

Inclusion and exclusion criteria followed the same methods as described for the vital registration data sources (Appendix Section 2).

Bias of categories of input data

The potential biases of the input data are the same as for other cancers (see above).

Methods

Overall methodological process

Vital registration system data were used as input to model deaths due to squamous-cell skin cancer.

Steps of analysis and data transformation processes

Since mortality estimates for non-melanoma skin cancer are only produced for squamous-cell carcinoma

under the assumption that basal-cell carcinoma causes almost no deaths, all mortalities reported as "C44" or "173" were mapped to the "squamous-cell carcinoma" GBD cause.

Model selection

The modelling strategy for non-melanoma skin cancer (squamous-cell carcinoma) followed the general CODEm process.

Model performance and sensitivity

The modelling performance and sensitivity for non-melanoma skin cancer (squamous-cell carcinoma) mirrored that of the general CODEm process.

Uncertainty intervals

Uncertainty was determined using standard CODEm methodology.

Results

Interpretation of results

Non-melanoma skin cancer mortality estimates are not available from other sources. GLOBOCAN, for example, does not report deaths due to non-melanoma skin cancer. Even though the data availability for non-melanoma skin cancer is poor, the fact that it is the most common incident cancer, with rates expected to rise, makes it a necessity to include the disease in the GBD framework.

Limitations

Cancer registry data for non-melanoma skin cancer incidence have to be interpreted with caution due to a substantial amount of underreporting or rules that only the first non-melanoma skin cancer has to be registered. Many cancer registries therefore do not include non-melanoma skin cancers at all. However, the information if registries capture NMSC or not is not consistently available. Therefore, no cancer registry data were used to estimate deaths due to squamous-cell carcinoma of the skin. For vital registration data, we make the assumption that there are no deaths due to basal-cell non-melanoma skin cancer, and therefore all deaths attributed to basal-cell carcinoma were included instead as squamous-cell carcinoma.

Covariates by cancer:

Lip and oral cavity cancer

Level	Covariate	Direction
1	Litres of alcohol consumed per capita	+
	Cumulative cigarettes (10 years)	+
	Cumulative cigarettes (20 years)	+
	Tobacco (cigarettes per capita)	+
	Log-transformed SEV scalar: Mouth Cancer	+
2	Age- and sex-specific SEV for high red meat	+
	Age- and sex-specific SEV for low vegetables	+
	Age- and sex-specific SEV for low fruit	+
	Healthcare Access and Quality Index	−
3	Education (years per capita)	−
	LDI (I$ per capita)	+
	Socio-demographic Index	+

Nasopharynx cancer

Level	Covariate	Direction
1	Litres of alcohol consumed per capita	+
	Cumulative cigarettes (10 years)	+
	Cumulative cigarettes (20 years)	+
	Tobacco (cigarettes per capita)	+
	Log-transformed SEV scalar: Nasopharynx Cancer	+
2	Age- and sex-specific SEV for low vegetables	+
	Population density (over 1000 ppl/sqkm, proportion)	+
	Healthcare Access and Quality Index	−
3	Education (years per capita)	−
	Age- and sex-specific SEV for low fruit	+
	LDI (I$ per capita)	-
	Socio-demographic Index	+

Oesophageal cancer

Level	Covariate	Direction
1	Litres of alcohol consumed per capita	+
	Log-transformed age-standardised SEV scalar: Oesophageal Cancer	+
	Mean BMI	+
	Smoking prevalence	+
2	Indoor air pollution (all cooking fuels)	+
	Tobacco (cigarettes per captia)	+
	Age- and sex-specific SEV for low vegetables	+
	Age- and sex-specific SEV for low fruit	+
	Healthcare Access and Quality Index	−
3	Education (years per capita)	−
	Sanitation (proportion with access)	−
	Improved water source (proportion with access)	−
	LDI (I$ per capita)	+
	Socio-demographic Index	+

Other pharynx cancer

Level	Covariate	Direction
1	*Litres of alcohol consumed per capita*	+
	Smoking prevalence	+
	Log-transformed SEV scalar: Other Pharynx Cancer	+
2	*Cumulative cigarettes (5 years)*	+
	Age- and sex-specific SEV for low fruit	+
	Age- and sex-specific SEV for low vegetables	+
	Population density (over 1000 ppl/sqkm, proportion)	+
	Population density (under 150 ppl/sqkm, proportion)	+
	Healthcare Access and Quality Index	–
3	*Education (years per capita)*	–
	LDI (I$ per capita)	+
	Socio-demographic Index	+

Stomach cancer

Level	Covariate	Direction
1	*Diet high in sodium*	+
	Tobacco (cigarettes per capita)	+
	Log-transformed SEV scalar: Stomach Cancer	+
	Log-transformed SEV scalar: Stomach Cancer	+
2	*Cumulative cigarettes (20 years)*	+
	Age- and sex-specific SEV for unsafe water	+
	Age- and sex-specific SEV for unsafe sanitation	+
	Mean BMI	+
	Sanitation (proportion with access)	–
	Improved water source (proportion with access)	–
	Healthcare Access and Quality Index	–
3	*Education (years per capita)*	–
	Age- and sex-specific SEV for low fruits	+
	Age- and sex-specific SEV for low vegetables	+
	LDI (I$ per capita)	+
	Socio-demographic Index	–

Testicular cancer

Level	Covariate	Direction
2	*Cumulative cigarettes (5 years)*	+
	Cumulative cigarettes (10 years)	+
	Cumulative cigarettes (15 years)	+
	Cumulative cigarettes (20 years)	+
	Tobacco (cigarettes per capita)	+
	Smoking prevalence	+
	Age- and sex-specific SEV for low fruits	+
	Age- and sex-specific SEV for low vegetables	+
	Healthcare Access and Quality Index	–
3	*Education (years per capita)*	–
	LDI (I$ per capita)	+
	Socio-demographic Index	+

Liver Cancer

Level	Covariate	Direction
1	*Litres of alcohol consumed per capita*	+
	HIV age-standardised prevalence	+
	Hepatitis B seroprevalence (HBsAg) age-standardised	+
	Hepatitis C seroprevalence (anti-HCV) age-standardised	+
	Log-transformed SEV scalar: Liver Cancer	+
2	*Hepatitis B 3-dose coverage (proportion)*	–
	Hepatitis B vaccine coverage (proportion), aged through time	–
	Intravenous drug use (age-standardised proportion)	+
	Cumulative cigarettes (20 years)	+
	Mean BMI	+
	Tobacco (cigarettes per capita)	+
	Healthcare Access and Quality Index	–
	Diabetes fasting plasma glucose (mmol/L), age-standardised 25+	+

Liver cancer (continued)

Level	Covariate	Direction
3	*Education (years per capita)*	–
	Age- and sex-specific SEV for high red meat	+
	LDI (I$ per capita)	–
	Socio-demographic Index	–

Gallbladder and biliary tract cancer

Level	Covariate	Direction
1	*Log-transformed SEV scalar: Gallbladder Cancer*	+
	Mean BMI	+
2	*Litres of alcohol consumed per capita*	+
	Cumulative cigarettes (5 years)	+
	Cumulative cigarettes (10 years)	+
	Smoking prevalence	+
	Tobacco (cigarettes per capita)	+
	Age- and sex-specific SEV for low fruit	+
	Age- and sex-specific SEV for low vegetables	+
	Diabetes age-atandardised prevalence (proportion)	+
	Healthcare Access and Quality Index	–
3	*Education (years per capita)*	–
	LDI (I$ per capita)	+
	Socio-demographic Index	-

Pancreatic cancer

Level	Covariate	Direction
1	*Cumulative cigarettes (10 years)*	+
	Cumulative cigarettes (20 years)	+
	Tobacco (cigarettes per capita)	+
	Log-transformed SEV scalar: Pancreas Cancer	+
	Mean BMI	+
2	*Age- and sex-specific SEV for high red meat*	+
	Litres of alcohol consumed per capita	+
	Age- and sex-specific SEV for low vegetables	+
	Energy unadjusted (kcal)	+
	Diabetes fasting plasma glucose (mmol/L), age-standardised 25+	+
	Diabetes age-standardised prevalence (proportion)	+
	Healthcare Access and Quality Index	–
3	*Education (years per capita)*	–
	Age- and sex-specific SEV for low fruit	+
	LDI (I$ per capita)	+
	Socio-demographic Index	+

Larynx cancer

Level	Covariate	Direction
1	*Litres of alcohol consumed per capita*	+
	Log-transformed SEV scalar: Larynx Cancer	+
2	*Smoking prevalence*	+
	Asbestos consumption (metric tons per year per capita)	+
	Age- and sex-specific SEV for low vegetables	+
	Cumulative cigarettes (10 years)	+
	Cumulative cigarettes (20 years)	+
	Population density (over 1000 ppl/sqkm, proportion)	+
	Healthcare Access and Quality Index	–
3	*Age- and sex-specific SEV for low fruit*	+
	LDI (I$ per capita)	+
	Socio-demographic Index	+

Tracheal, bronchus, and lung cancer

Level	Covariate	Direction
1	*Asbestos consumption (metric tons per year per capita)*	+
	Smoking prevalence	+
	Secondhand smoke	+
	Log-transformed SEV scalar: Lung Cancer	+
	Log-transformed age-standardised SEV scalar: Lung Cancer	+
2	*Indoor air pollution (all cooking fuels)*	+
	Cumulative cigarettes (10 years)	+
	Cumulative cigarettes (20 years)	+
	Outdoor air pollution ($PM_{2.5}$)	+
	Residential radon	+
	Diabetes fasting plasma glucose (mmol/L), age-standardised 25+	+
	Healthcare Access and Quality Index	–
3	*Education (years per capita)*	–
	LDI (I$ per capita)	+
	Socio-demographic Index	+

Malignant skin melanoma

Level	Covariate	Direction
1	*Litres of alcohol consumed per capita*	+
2	*Latitude under 15 (proportion)*	–
	Latitude 15 to 30 (proportion)	–
	Latitude 30 to 45 (proportion)	–
	Latitude over 45 (proportion)	–
	Healthcare Access and Quality Index	–
3	*Education (years per capita)*	–
	LDI (I$ per capita)	–
	Socio-demographic Index	+

Non-melanoma skin cancer

Level	Covariate	Direction
1	*Cumulative cigarettes (5 years)*	+
	Cumulative cigarettes (10 years)	+
	Cumulative cigarettes (15 years)	+
	Smoking prevalence	+
2	*Average latitude*	–
	Healthcare Access and Quality Index	–
3	*Education (years per capita)*	–
	LDI (I$ per capita)	–
	Socio-demographic Index	+

Breast cancer

Level	Covariate	Direction
1	*Litres of alcohol consumed per capita*	+
	Mean BMI	+
	Log-transformed SEV scalar: Breast Cancer	+
2	*Age-specific fertility rate*	–
	Total fertility rate	–
	Age- and sex-specific SEV for low fruit	+
	Age- and sex-specific SEV for low vegetables	+
	Cumulative cigarettes (10 years)	+
	Cumulative cigarettes (20 years)	+
	Smoking prevalence	+
	Diabetes fasting plasma glucose (mmol/L), age-standardised 25+	+
	Healthcare Access and Quality Index	–
3	*LDI (I$ per capita)*	–
	Socio-demographic Index	+

Cervical cancer

Level	Covariate	Direction
1	*Cumulative cigarettes (5 years)*	+
	HIV age-standardised prevalence	+
2	*Age-specific fertility rate*	+
	Total fertility rate	+
	Smoking prevalence	+
	Age- and sex-specific SEV for low fruit	+
	Age- and sex-specific SEV for low vegetables	+
	Healthcare Access and Quality Index	–
3	*Education (years per capita)*	–
	LDI (I$ per capita)	–
	Socio-demographic Index	–

Uterine cancer

Level	Covariate	Direction
1	*Log-transformed SEV scalar: Uterus Cancer*	+
	Mean BMI	+
2	*Cumulative cigarettes (5 years)*	+
	Cumulative cigarettes (10 years)	+
	Smoking prevalence	+
	Tobacco (cigarettes per capita)	+
	Diabetes age-standardized prevalence (proportion)	+
	Total fertility rate	–
	Age- and sex-specific SEV for low fruit	+
	Age- and sex-specific SEV for low vegetables	+
	Healthcare Access and Quality Index	–
3	*Education (years per capita)*	–
	LDI (I$ per capita)	+
	Socio-demographic Index	+

Prostate cancer

Level	Covariate	Direction
1	*Log-transformed SEV scalar: Prostate Cancer*	+
2	*Smoking prevalence*	+
	Healthcare Access and Quality Index	–
3	*Education (years per capita)*	–
	LDI (I$ per capita)	–
	Socio-demographic Index	+

Kidney cancer

Level	Covariate	Direction
1	Cumulative cigarettes (5 years)	+
	Cumulative cigarettes (10 years)	+
	Cumulative cigarettes (15 years)	+
	Mean BMI	+
	Log-transformed SEV scalar: Kidney Cancer	+
2	Litres of alcohol consumed per capita	+
	Diabetes age-standardised prevalence (proportion)	+
	Systolic blood pressure (mmHg)	+
	Smoking prevalence	+
	Healthcare Access and Quality Index	–
3	Education (years per capita)	–
	LDI (I$ per capita)	+
	Socio-demographic Index	+

Bladder cancer

Level	Covariate	Direction
1	Schistosomiasis prevalence (proportion)	+
	Cumulative cigarettes (10 years)	+
	Smoking prevalence	+
	Log-transformed SEV scalar: Bladder Cancer	+
2	Litres of alcohol consumed per capita	+
	Diabetes fasting plasma glucose (mmol/L), age-standardised 25+	+
	Age- and sex-specific SEV for low vegetables	+
	Healthcare Access and Quality Index	–
3	Age- and sex-specific SEV for low fruits	+
	LDI (I$ per capita)	+
	Socio-demographic Index	+

Brain and nervous system cancer

Level	Covariate	Direction
1	Litres of alcohol consumed per capita	+
	Cumulative cigarettes (10 years)	+
	Smoking prevalence	+
2	Cholesterol (total, mean per capita)	+
	Systolic blood pressure (mmHg)	+
	Age- and sex-specific SEV for high red meat	+
	Age- and sex-specific SEV for low vegetables	+
	Age- and sex-specific SEV for low fruit	+
	Healthcare Access and Quality Index	–
3	Education (years per capita)	–
	LDI (I$ per capita)	+
	Socio-demographic Index	+

Thyroid cancer

Level	Covariate	Direction
1	*Litres of alcohol consumed per capita*	+
	Log-transformed SEV scalar: Thyroid Cancer	+
2	*Age- and sex-specific SEV for low vegetables*	+
	Age- and sex-specific SEV for high red meat	+
	Tobacco (cigarettes per capita)	+
	Mean BMI	+
	Healthcare Access and Quality Index	–
3	*Education (years per capita)*	–
	Sanitation (proportion with access)	–
	Improved water source (proportion with access)	–
	Age- and sex-specific SEV for low fruits	+
	LDI (I$ per capita)	+
	Socio-demographic Index	+

Mesothelioma

Level	Covariate	Direction
1	*Asbestos consumption (metric tons per year per capita)*	+
	Cumulative cigarettes (5 years)	+
	Log-transformed SEV scalar: Mesothelioma	+
	Log-transformed age-standardized SEV scalar: Mesothelioma	+
	Smoking prevalence	+
2	*Gold production (binary)*	+
	Indoor air pollution (all cooking fuels)	+
	Population density (over 1000 ppl/sqkm, proportion)	+
	Healthcare Access and Quality Index	–
3	*Education (years per capita)*	–
	LDI (I$ per capita)	–
	Socio-demographic Index	+

Hodgkin lymphoma

Level	Covariate	Direction
2	*Healthcare Access and Quality Index*	–
3	*Education (years per capita)*	–
	LDI (I$ per capita)	–
	Socio-demographic Index	–

Non-Hodgkin lymphoma

Level	Covariate	Direction
2	*Cumulative cigarettes (5 years)*	+
	Cumulative cigarettes (10 years)	+
	Cumulative cigarettes (15 years)	+
	Cumulative cigarettes (20 years)	+
	Litres of alcohol consumed per capita	+
	Smoking prevalence	+
	Mean BMI	+
	Healthcare Access and Quality Index	–
3	*Total fertility rate*	–
	LDI (I$ per capita)	+
	Socio-demographic Index	+

Multiple myeloma

Level	Covariate	Direction
1	*Litres of alcohol consumed per capita*	+
	Smoking prevalence	+
	Tobacco (cigarettes per capita)	+
2	*Age- and sex-specific SEV for low vegetables*	+
	Age- and sex-specific SEV for low fruits	+
	Age- and sex-specific SEV for high red meat	+
	Mean BMI	+
	Sanitation (proportion with access)	–
	Improved water source (proportion with access)	–
	Healthcare Access and Quality Index	–
3	*Education (years per capita)*	–
	LDI (I$ per capita)	+
	Socio-demographic Index	+

Leukaemia

Level	Covariate	Direction
1	*Log-transformed age-standardised SEV scalar: Leukaemia*	+
	Log-transformed SEV scalar: Leukaemia	+
2	*Litres of alcohol consumed per capita*	+
	Mean BMI	+
	Cumulative cigarettes (10 years)	+
	Cumulative cigarettes (20 years)	+
	Tobacco (cigarettes per capita)	+
	Healthcare Access and Quality Index	–
3	*Education (years per capita)*	–
	LDI (I$ per capita)	+
	Socio-demographic Index	–

Myelodysplastic, myeloproliferative, other haemopoietic neoplasms

Level	***Covariate***	***Direction***
1	*Log-transformed age-standardised SEV scalar: Leukaemia*	+
	Log-transformed SEV scalar: Leukaemia	+
2	*Litres of alcohol consumed per capita*	+
	Cumulative cigarettes (5 years)	+
	Cumulative cigarettes (10 years)	+
	Cumulative cigarettes (15 years)	+
	Cumulative cigarettes (20 years)	+
	Smoking prevalence	+
	Tobacco (cigarettes per capita)	+
	Healthcare Access and Quality Index	–
3	*Education (years per capita)*	–
	LDI (I$ per capita)	+
	Socio-demographic Index	+

Other malignant cancers

Level	***Covariate***	***Direction***
1	*Smoking prevalence*	+
	Tobacco (cigarettes per capita)	+
2	*Age- and sex-specific SEV for low vegetables*	+
	Age- and sex-specific SEV for low fruits	+
	Age- and sex-specific SEV for low nuts and seeds	+
	PUFA adjusted (percent)	–
	Healthcare Access and Quality Index	–
3	*Education (years per capita)*	–
	LDI (I$ per capita)	+
	Socio-demographic Index	+

Other neoplasms

Level	***Covariate***	***Direction***
2	*Healthcare Access and Quality Index*	–
3	*Education (years per capita)*	–
	LDI (I$ per capita)	+
	Socio-demographic Index	–

Colon and rectum cancer

Level	Covariate	Direction
1	Mean BMI	+
	Tobacco (cigarettes per capita)	+
	Total physical activity (MET-min/week), age-specific	–
	Log-transformed SEV scalar: Colorectal Cancer	+
	Age- and sex-specific SEV for high red meat	+
2	Litres of alcohol consumed per capita	+
	PUFA adjusted (percent)	–
	Age- and sex-specific SEV for low vegetables	+
	Age- and sex-specific SEV for low fibre	+
	Age- and sex-specific SEV for low calcium	+
	Cumulative cigarettes (5 years)	+
	Diabetes fasting plasma glucose (mmol/L), age-standardised 25+	+
3	Education (years per capita)	–
	Age- and sex-specific SEV for low milk	+
	Age- and sex-specific SEV for low fruit	+
	Age- and sex-specific SEV for low nuts and seeds	+
	Healthcare Access and Quality Index	–
	LDI (I$ per capita)	+
	Socio-demographic Index	+

Ovarian cancer

Level	Covariate	Direction
1	Litres of alcohol consumed per capita	+
	Cumulative cigarettes (10 years)	+
	Cumulative cigarettes (20 years)	+
	Contraception (modern) prevalence (proportion)	–
	Log-transformed SEV scalar: Ovary Cancer	+
2	Asbestos consumption (metric tons per year per capita)	+
	Smoking prevalence	+
	Total fertility rate	–
	Energy unadjusted (kcal)	+
	Mean BMI	+
	Diabetes age-standardized prevalence (proportion)	+
	Healthcare Access and Quality Index	`–
3	Education (years per capita)	–
	Age- and sex-specific SEV for low fruits	+
	Age- and sex-specific SEV for low vegetables	+
	LDI (I$ per capita)	–
	Socio-demographic Index	+

References

1 Waterhouse J, Muir C, Shanmugaratnam K, Powell J. Cancer Incidence in Five Continents IV. Lyon: IARC, 1982.

2 Curado M, Edwards B, Shin H, *et al.* Cancer Incidence in Five Continents IX. Lyon: IARC, 2007 http://www.iarc.fr/en/publications/pdfs-online/epi/sp160/CI5vol9-A.pdf.

3 Muir C, Mack T, Powell J, Whelan S. Cancer Incidence in Five Continents V. Lyon: IARC, 1987.

4 Parkin D, Muir C, Whelan S, Gao Y, Ferlay J, Powell J. Cancer Incidence in Five Continents VI. Lyon: IARC, 1992.

5 Parkin D, Whelan S, Ferlay J, Raymond L, Young J. Cancer Incidence in Five Continents VII. Lyon: IARC, 1997.

6 Parkin D, Whelan S, Ferlay J, Teppo L, Thomas D. Cancer Incidence in Five Continents VIII. Lyon: IARC, 2002.

7 Forman D, Bray F, Brewster D, *et al.* Cancer Incidence in Five Continents X. 2013. http://ci5.iarc.fr.

8 Engholm G, Ferlay J, Christensen N, *et al.* NORDCAN: Cancer Incidence, Mortality, Prevalence and Survival in the Nordic Countries, Version 7.3 Association of the Nordic Cancer Registries. Danish Cancer Society. 2016; published online Aug 7. http://www.ancr.nu.

9 Steliarova-Foucher E, O'Callaghan M, Ferlay J, Masuyer E, Forman D, Comber H, Bray F. European Cancer Observatory: Cancer Incidence, Mortality, Prevalence and Survival in Europe. Version 1.0 European Network of Cancer Registries, International Agency for Research on Cancer. 2012; published online Sept. http://eco.iarc.fr.

10 de Martel C, Maucort-Boulch D, Plummer M, Franceschi S. World-wide relative contribution of hepatitis B and C viruses in hepatocellular carcinoma. *Hepatology* 2015; **62**: 1190–200.

11 Hong TP, Gow P, Fink M, *et al.* Novel population-based study finding higher than reported hepatocellular carcinoma incidence suggests an updated approach is needed. *Hepatology* 2016; **63**: 1205–12.

12 GBD 2015 Risk Factors Collaborators. Global, regional, and national comparative risk assessment of 79 behavioural, environmental and occupational, and metabolic risks or clusters of risks, 1990–2015: a systematic analysis for the Global Burden of Disease Study 2015. *The Lancet* 2016; **388**: 1659–724.

13 International Agency for Research on Cancer, World Health Organization. GLOBOCAN estimated cancer incidence, mortality, and prevalence worldwide in 2012. Lyon, France: IARC, 2014 http://globocan.iarc.fr/Default.aspx (accessed April 19, 2016).

14 Karagas MR, Greenberg ER, Spencer SK, Stukel TA, Mott LA. Increase in incidence rates of basal cell and squamous cell skin cancer in New Hampshire, USA. New Hampshire Skin Cancer Study Group. *Int J Cancer* 1999; **81**: 555–9.

Rheumatic heart disease

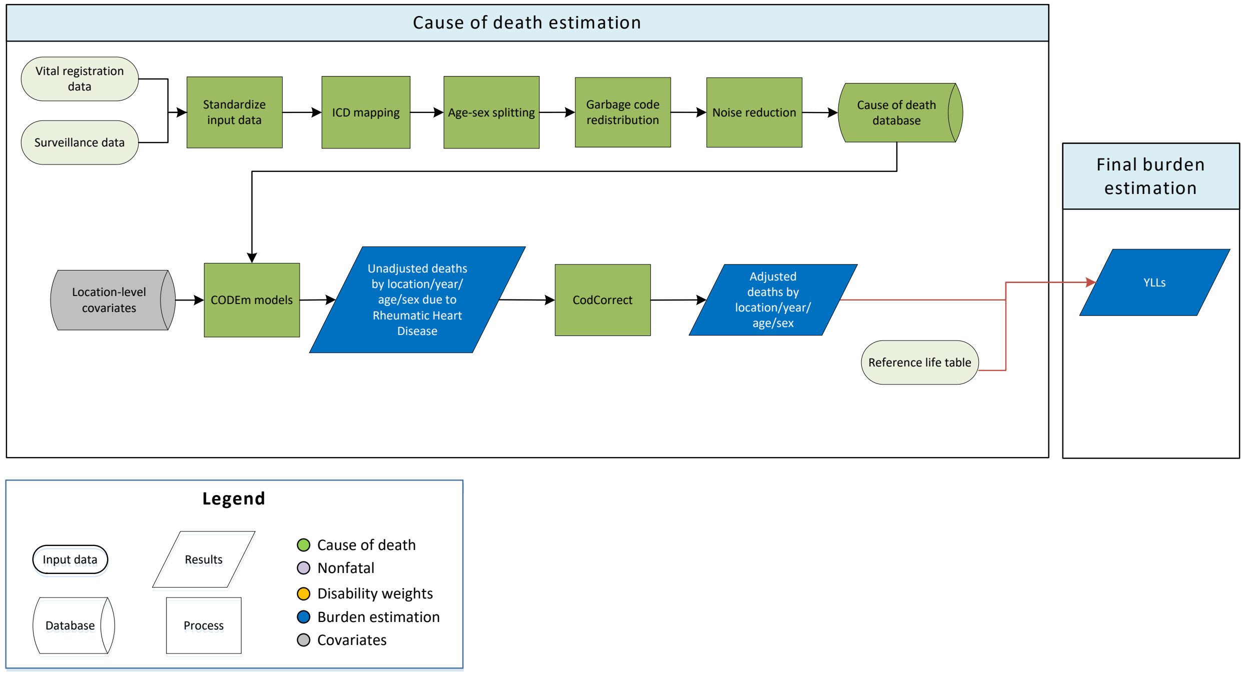

Input data

Vital registration and surveillance data were used to model rheumatic heart disease. We outliered ICD8 and ICD9 BTL datapoints which were inconsistent with the rest of the data and created implausible time trends. We also outliered datapoints which were too high after the redistribution process in a number of age groups. In addition, we outliered verbal autopsy datapoints in Nepal and Pakistan which created an implausibly low cause fraction.

Modelling strategy

We used a standard CODEm approach to model deaths from rheumatic heart disease. There have been no substantive changes from the approach used in GBD 2017, including any covariate changes.

Table 1: Selected covariates for CODEm models, rheumatic heart disease

Level	Covariate	Transformation	Direction
1	Rheumatic heat disease summary exposure value scalar	None	1
1	Improved water (proportion)	None	-1
1	Malnutrition	None	1
1	Sanitation (proportion with access)	None	-1
2	Healthcare access and quality index	None	-1
3	Lag distributed income per capita (I$)	Log	-1
3	Socio-demographic Index	None	-1
3	Education (years per capita)	None	-1

Ischaemic Heart Disease

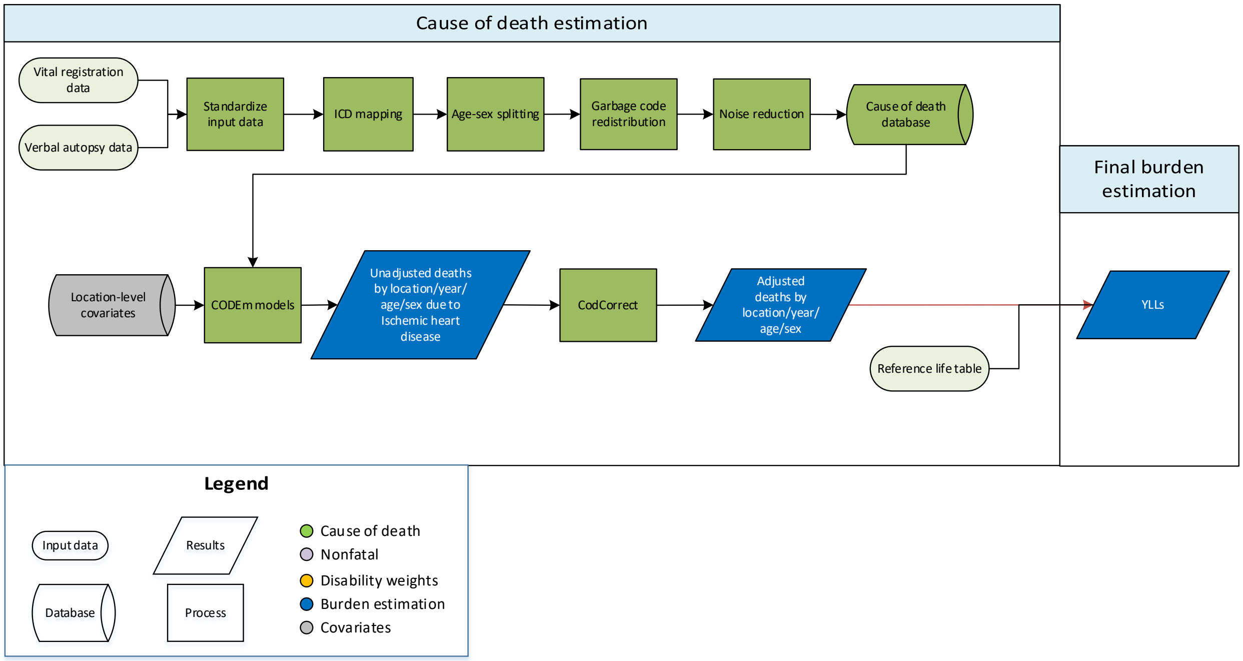

Input data

Vital registration and verbal autopsy data were used to model ischaemic heart disease. We outliered verbal autopsy data in countries and subnational locations where high-quality vital registration data were also available. We also outliered non-representative subnational verbal autopsy data points, ICD8 and ICD9BTL data points which were inconsistent with the rest of the data and created implausible time trends, and data in a number of Indian states identified by experts as poor-quality.

Modelling strategy

We used a standard CODEm approach to model deaths from ischemic heart disease. For GBD 2019, adjusted dietary covariates for consumption of fruits, omega-3 fatty acids, vegetables, nuts and seeds, and polyunsaturated fatty acids were replaced with the summary exposure value scalars for diet low in each of these factors. The direction for each dietary covariate was changed from -1 to 1 to as our *a priori* assumption is that low levels of intake of these dietary factors are associated with increasing mortality risk from ischaemic heart disease. We changed the direction of the alcohol variable from 0 to 1 to reflect our *a priori* hypothesis about the expected direction of the association between this risk factor and mortality risk of ischaemic heart disease. In addition, we changed the level of the covariate for trans fatty acid from 1 to 3. Besides these covariate changes, there are no other substantive changes from the approach used in GBD 2017.

Table: Selected covariates for CODEm models, ischaemic heart disease

Covariate	Transformation	Level	Direction
Summary exposure value, IHD	None	1	1
Cholesterol (total, mean per capita)	None	1	1
Smoking prevalence	None	1	1
Systolic blood pressure (mmHg)	None	1	1
Mean BMI	None	2	1
Elevation over 1500m (proportion)	None	2	-1
Fasting plasma glucose	None	2	1
Outdoor pollution ($PM_{2.5}$)	None	2	1
Indoor air pollution	None	2	1
Healthcare access and quality index	None	2	-1
Lag distributed income per capita (I$)	Log	3	-1
Summary exposure value, omega-3	None	3	1
Summary exposure value, fruits	None	3	1
Summary exposure value, vegetables	None	3	1
Summary exposure value, nuts and seeds	None	3	1
Pulses/legumes (kcal/capita, unadjusted)	None	3	-1
Summary exposure value, PUFA (percent, adjusted)	None	3	1
Alcohol (litres per capita)	None	3	1
Trans fatty acid	None	3	1

Stroke

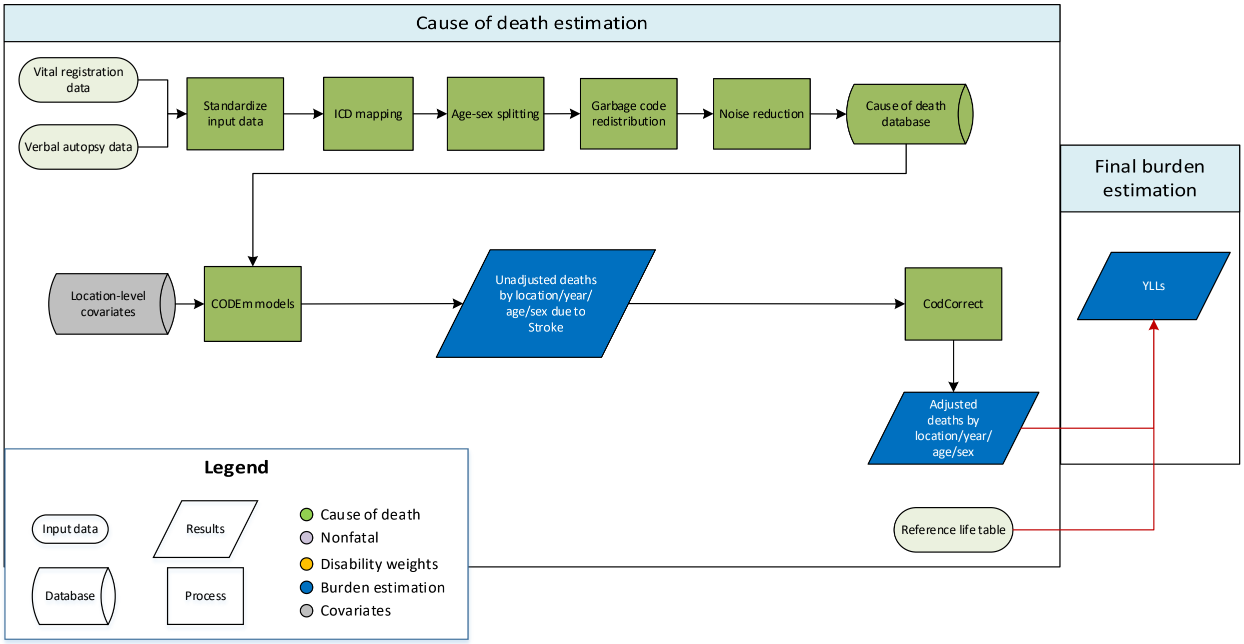

Input data

Verbal autopsy and vital registration data were used to model cerebrovascular disease (stroke). We reassigned deaths from verbal autopsy reports for cerebrovascular disease to the parent cardiovascular disease for both sexes for those under 20 years of age. We outliered non-representative subnational verbal autopsy datapoints. We also outliered ICD8, ICD9BTL, and tabulated ICD10 datapoints which were inconsistent with the rest of the data and created implausible time trends. Datapoints from sources which were implausibly low in all age groups and data points that were causing the regional estimates to be improbably high were outliered.

Modelling strategy

We used a standard CODEm approach to model deaths from stroke. The covariates included in the ensemble modelling process are listed in the table below. For GBD 2019, adjusted dietary covariates for consumption of fruits, omega-3 fatty acids, vegetables, nuts and seeds, and polyunsaturated fatty acids (PUFA) were replaced with the summary exposure value scalars for diet low in each of these factors. The direction for each dietary covariate was changed from -1 to 1 to as our a priori assumption is that low levels of intake of these dietary factors are associated with increasing mortality risk from stroke. We dropped the dietary covariate for whole grains (kcal/capita, adjusted) and the socio-demographic index covariate as exploratory analyses indicated that these variables were not predictive of stroke mortality. In addition, we changed the direction of the alcohol consumption covariate from 0 to 1 to reflect the expected direction of the association for this risk factor with stroke mortality. Apart from these covariate changes, there are no substantive changes from the approach used in GBD 2017.

Table: Selected covariates for CODEm models, stroke

Covariate	Transformation	Level	Direction
Summary exposure variable, stroke	None	1	1
Cholesterol (total, mean per capita)	None	1	1
Smoking prevalence	None	1	1
Systolic blood pressure (mmHg)	None	1	1
Mean BMI	None	2	1
Elevation over 1,500m (proportion)	None	2	-1
Fasting plasma glucose	None	2	1
Outdoor pollution ($PM_{2.5}$)	None	2	1
Indoor air pollution	None	2	1
Healthcare Access and Quality Index	None	2	-1
Lag distributed income per capita (I$)	Log	3	-1
Summary exposure value, omega-3	None	3	1
Summary exposure value, fruits	None	3	1
Summary exposure value, vegetables	None	3	1
Summary exposure value, nuts and seeds	None	3	1
Pulses/legumes (kcal/capita, unadjusted)	None	3	-1
Summary exposure value, PUFA adjusted (percent)	None	3	1
Alcohol (litres per capita)	None	3	1
Trans fatty acid	None	3	1

Hypertensive Heart Disease

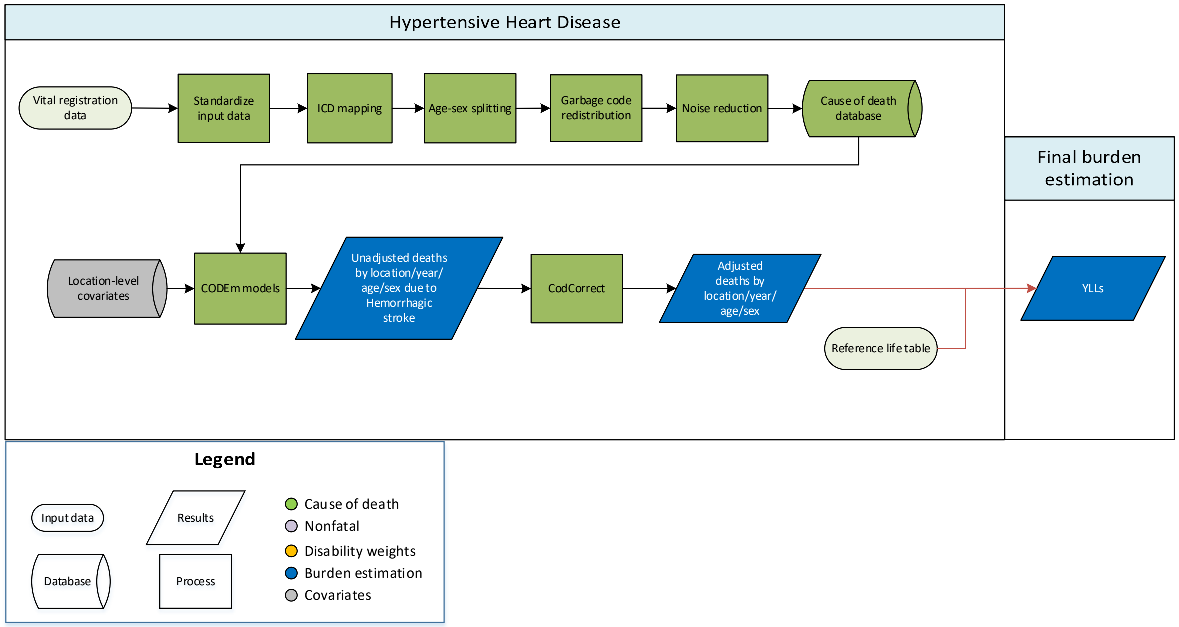

Input data

Vital registration data were used to model cause-specific mortalty for hypertensive heart disease. We outliered ICD9BTL data points, which were inconsistent with the rest of the data and created implausible time trends. In addition, we outliered vital registration data from Grenada in 2017 for being implausibly low across all age groups.

Modelling strategy

We used a standard CODEm approach to model deaths from hypertensive heart disease. For GBD 2019, adjusted dietary covariates for consumption of fruits, omega-3 fatty acids, vegetables, nuts and seeds, and polyunsaturated fatty acids were replaced with the summary exposure value scalars for diet low in each of these factors. The direction for each dietary covariate was changed from -1 to 1 to as our *a priori* assumption is that low levels of intake of these dietary factors are associated with increasing mortality risk from hypertensive heart disease. We also changed the direction of the covariates for alcohol and socio-demographic index from 0 to 1 to reflect the expected direction of these covariates with mortality risk. Apart from these covariate updates, there are no other substantive changes from the approach used in GBD 2017.

Table: Selected covariates for CODEm models, hypertensive heart disease

Covariate	Transformation	Level	Direction
Systolic blood pressure (mmHg)	None	1	1
Cholesterol (total, mean per capita)	None	2	1
Smoking prevalence	None	2	1
Mean BMI	None	2	1
Healthcare access and quality index	None	2	-1
Lag distributed income per capita (I$)	Log	3	-1
Socio-demographic Index	None	3	1
Alcohol (litres per capita)	None	3	1
Summary exposure value, omega-3	None	3	1
Summary exposure value, fruits	None	3	1
Summary exposure value, nuts and seeds	None	3	1
Summary exposure value, PUFA	None	3	1
Summary exposure value, vegetables	None	3	1
Pulses/legumes (kcal/capita, unadjusted)	None	3	-1
Trans fatty acid (percent)	None	3	1

Chronic Respiratory Diseases

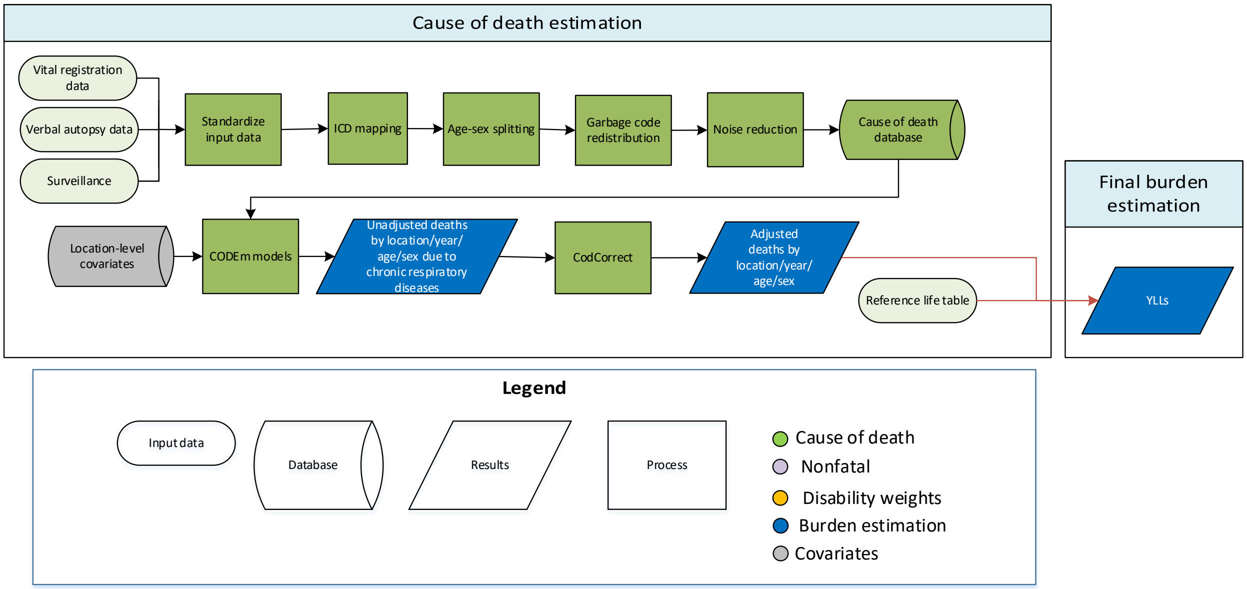

Input data

Sources used to estimate chronic respiratory disease mortality included vital registration, verbal autopsy, and surveillance data from China. Our outlier criteria excluded data points that (1) were implausibly high or low, (2) substantially conflicted with established age or temporal patterns, or (3) significantly conflicted with other data sources conducted from the same locations or locations with similar characteristics (ie, Socio-demographic Index).

Modelling strategy

The standard CODEm modelling approach was applied to estimate deaths due to chronic respiratory diseases. Separate models were conducted for male and female mortality, and the age range for both models was 1 to 95+ years.

Key Changes from GBD 2017

- We added estimates for the following new locations: Monaco, San Marino, Cook Islands, Nauru, Niue, Palau, Tokelau, Tuvalu, Monaco, San Marino, St Kitts and Nevis
- We added subnational location data for the following: Italy, Poland, Pakistan, the Philippines, and Nigeria
- We excluded all MCCD (the very incomplete hospital death data largely from urban areas) and all SCD (earlier verbal autopsy data using lesser quality instruments and analysis) from India, based on discussions with GBD India collaborators. Thus, the estimates are driven by the more recent higher quality SRS verbal autopsy data and covariates.
- Healthcare quality and access index covariate changed to a level 2 covariate from level 1.
- Smoking prevalence and indoor air pollution both moved to a level 1 covariate from level 2.
- We removed the covariate SEV for chronic respiratory disease.
- The SDI covariate was allowed to take a positive or negative direction in GBD 2017, but was specified to only be selected if a negative association was detected in GBD 2019.

The following covariates were used for GBD 2019:

Level	Covariate	Direction
1	indoor air pollution (all cooking fuels)	+
	cumulative cigarettes (10 years)	+
	cumulative cigarettes (5 years)	+
	smoking prevalence	+
2	healthcare quality and access index	-
	outdoor air pollution ($PM_{2.5}$)	+
	population above 1500m elevation (proportion)	+
3	LDI (I$ per capita)	-
	education (years per capita)	-
	socio-demographic index	-
	population between 500 and 1,500m elevation (proportion)	+
	population density over 1,000 people/kilometer^2 (proportion)	+

Chronic respiratory diseases served as a "parent" to the following causes:

- chronic obstructive pulmonary disease
- pneumoconiosis (silicosis, asbestosis, coal worker's pneumoconiosis, other pneumoconiosis)
- asthma
- interstitial lung disease and pulmonary sarcoidosis
- other chronic respiratory diseases

The unadjusted death estimates for all these "child" causes are summed and fit to the distribution of deaths estimated for the "parent" during the CODCorrect adjustment process. This results in deaths recorded using non-specific coding systems, such as verbal autopsy, being included in the parent model and redistributed to the child models proportionately. This approach assumes that deaths reported in non-specific data-sources have the same underlying distribution of specific causes as deaths reported in more specific data-sources.

Covariate Influences:

The following plots show the influence of each covariate on the four CODEm models (male global, male data rich, female global, and female data rich). A positive standardized beta (to the right) means that the covariate was associated with increased death. A negative standardized beta (to the left) means the covariate was associated with decreased death.

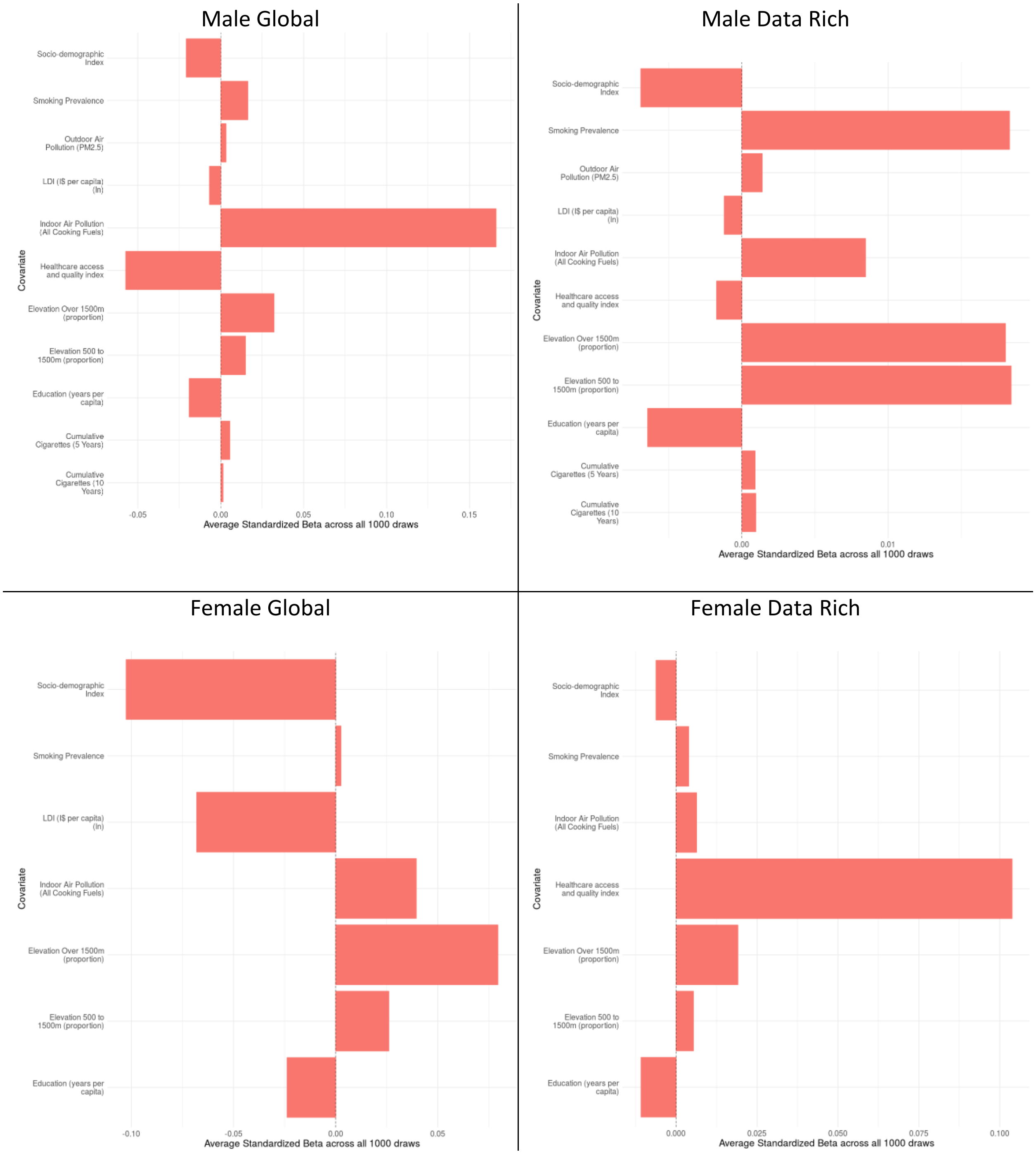

Peptic Ulcer Disease

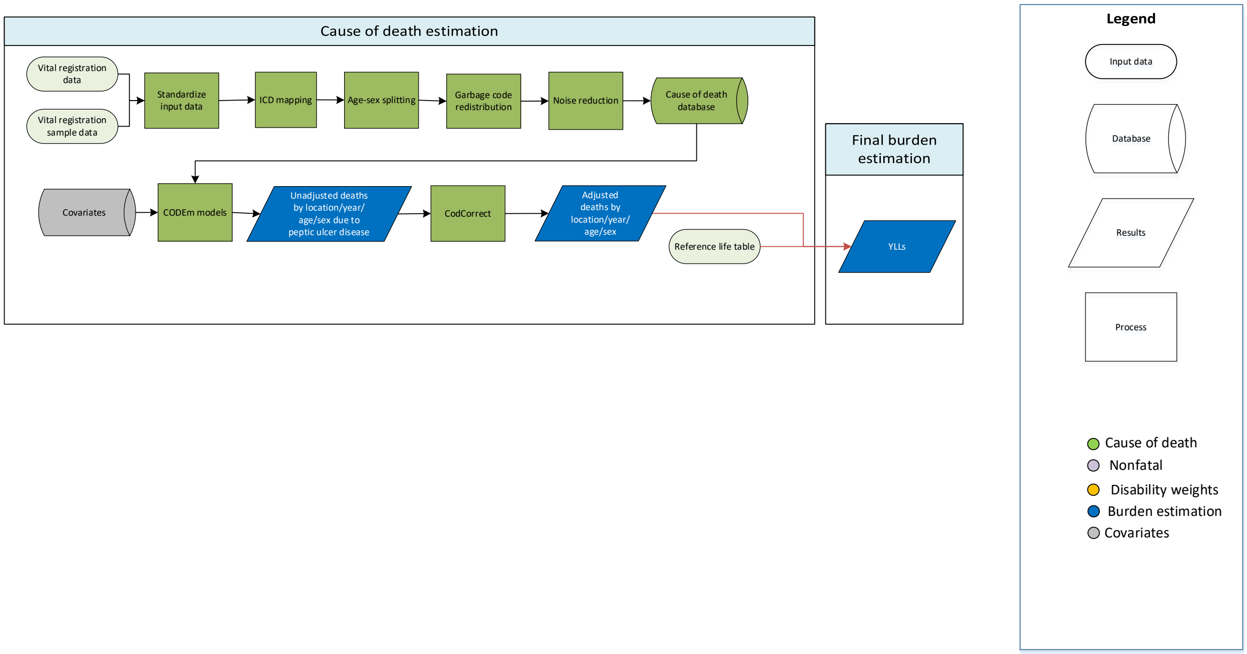

Input data

Data used to estimate unadjusted mortality of peptic ulcer disease consisted of vital registration data and vital registration sample data from those sources in the cause of death (COD) database that use ICD9 or ICD10 codes and report un-tabulated (individual) deaths. We marked data as outliers and excluded them in instances where garbage code redistribution and noise reduction, in combination with small sample sizes, resulted in unreasonable cause fractions or unreasonable time, age, or spatial trends; data from Tibet, Fiji, Kiribati, Palestine, Stockholm, and Mozambique were excluded for these reasons. In situations where unreasonable temporal and spatial trends were observed at transitions between data sources, higher-quality data-sources were retained and lower-quality sources were excluded; this affected Kazakhstan, at the transition between ICD9-BTL and ICD10 coding, and subnational locations in India, where vital registration data biased toward in-hospital deaths (MCCD) were available for urban locations only.

Modelling strategy

We modelled deaths due to peptic ulcer disease with a standard CODEm model. The model followed standard parameters, with the exception that the start age of the model was 1 year instead of 0 and the linear floor rate was lowered to 0.0001 in order to better capture low data.

Covariates entered into CODEm were the same in GBD 2019 as GBD 2017, with the following exceptions: covariates related to water and sanitation were promoted from level 2 to level 1, the alcohol covariate was demoted from level 1 to level 2, maternal education was replaced by a general education covariate, and the adjusted vegetable covariate was replaced by an unadjusted vegetable covariate and forced to take a negative direction (or not be selected). A complete list is provided in the table below.

Covariate	Level	Direction
Sanitation, proportion with access	1	-1
Scaled exposure variable for unsafe water source	1	1

Smoking prevalence	1	1
Cumulative cigarettes (10 years)	1	1
Cumulative cigarettes (5 years)	1	1
Litres of alcohol consumed per capita	2	1
Vegetables (grams, unadjusted)	2	-1
Healthcare access and quality index	2	-1
Lag distributed income (per capita)	3	-1
Education (years per capita)	3	-1
Socio-demographic Index	3	-1

In CoDCorrect estimates for peptic ulcer disease and gastritis and duodenitis were first adjusted to sum to all upper digestive disease deaths, and then to sum to all-cause mortality with all other causes.

Appendicitis

Flowchart

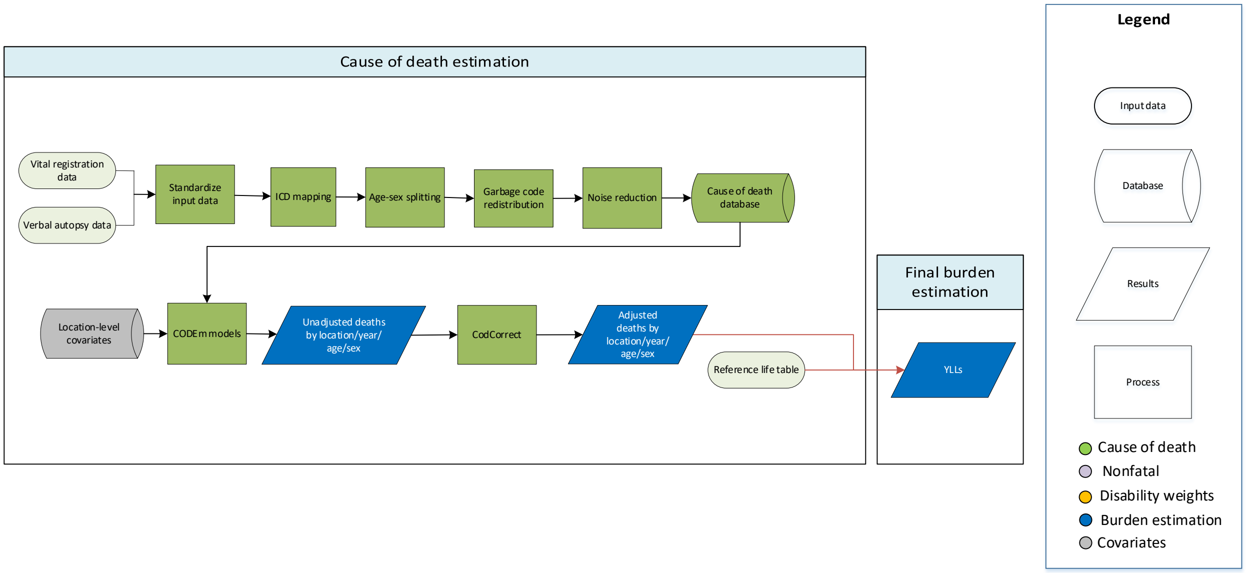

Input data

Data used to estimate mortality of appendicitis consisted of vital registration and verbal autopsy data from the cause of death (COD) database. Outliers were identified if data violated well-established time or age trends. We also excluded data in instances where garbage code redistribution and noise reduction, in combination with small sample sizes, resulted in unreasonable cause fractions.

Modelling strategy

The estimation strategy used for fatal appendicitis is largely similar to methods used in GBD 2017. A standard CODEm model with location-level covariates was used to model deaths due to appendicitis with age restrictions for death estimations of 1 year for lower bound and 95+ for upper bound (see appendix section on CODEm method for details). Separate models were conducted for male and female mortality. We hybridised separate global and data-rich models to acquire unadjusted results, which we finalised and adjusted using CodCorrect to reach final YLLs due to appendicitis.

Key changes from GBD 2017

- We added estimates for the following new locations: Monaco, San Marino, Cook Islands, and Saint Kitts and Nevis.
- We added subnational location data for the following: Italy, Poland, Pakistan, the Philippines, and Nigeria.
- We excluded the maternal care and immunisation (MCI) covariate because it is redundant with the Healthcare Access and Quality Index covariate that was pre-existing in the model. The MCI covariate is often used as a proxy for health system access measured through clinic accessibility, attendance, and immunisation status.
- We replaced adjusted dietary covariates with age-sex specific scaled exposure variable covariates with a direction of 1.
- We changed the direction of Socio-demographic Index covariate from 0 to -1.

The following table has the full list of covariates used for appendicitis.

Table 1. Covariates used in appendicitis mortality modelling

Level	Covariate	Direction
2	Age-sex-specific scaled exposure variable for low fruit consumption	+
	Age-sex-specific scaled exposure variable for low vegetable consumption	+
	Healthcare Access and Quality Index	-
3	Socio-demographic Index	-
	Education (years per capita)	-
	Log LDI ($I per capita)	-

Inguinal, femoral, and abdominal hernia

Flowchart

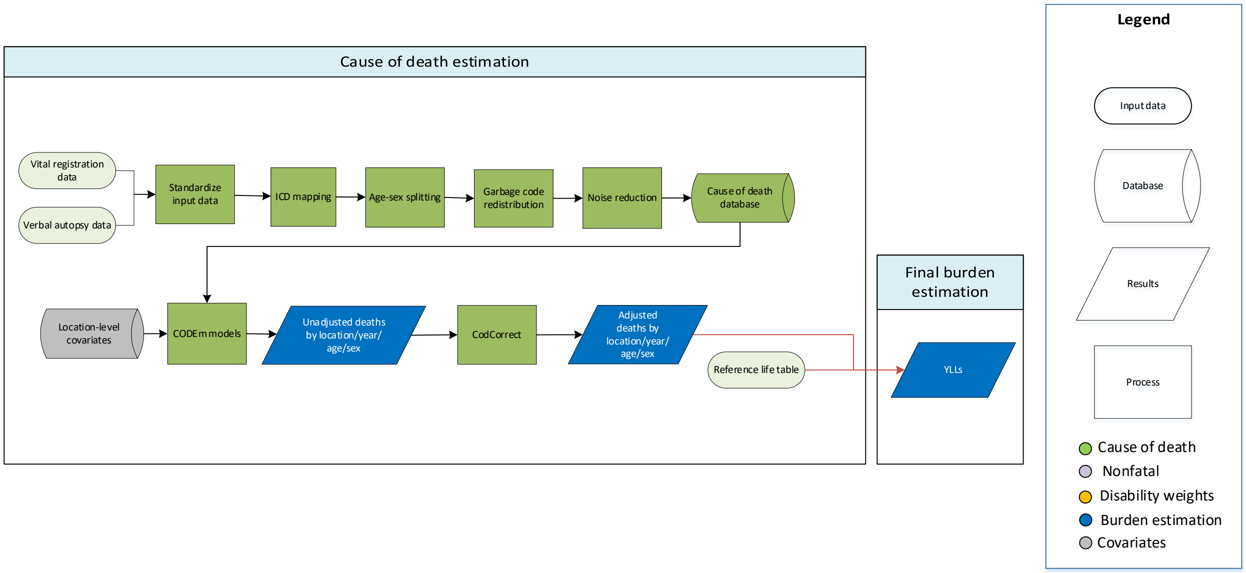

Input data

Data used to estimate mortality of inguinal, femoral, and abdominal hernia consisted of vital registration and verbal autopsy data from the cause of death (COD) database. Outliers were identified by systematic examination of datapoints for all location-years. Data that violated well-established time or age trends were marked as outliers and excluded. Data were also marked as outliers in instances where garbage code redistribution and noise reduction, in combination with small sample sizes, resulted in unreasonable cause fractions. Methods for assigning outlier status were consistent across both vital registration and verbal autopsy data.

Modelling strategy

The estimation strategy used for fatal inguinal, femoral, and abdominal hernia is largely similar to methods used in GBD 2017. A standard CODEm model with location-level covariates was used to model deaths due to inguinal, femoral, and abdominal hernia (see appendix section 3.1 for details). Separate models were conducted for male and female mortality. We hybridised separate global and data-rich models to acquire unadjusted results, which we finalised and adjusted using CodCorrect to reach final YLLs due to inguinal, femoral, and abdominal hernia.

Key changes from GBD 2017

- We added estimates for the following new locations: Monaco, San Marino, and Saint Kitts and Nevis.
- We added subnational location data for the following: Italy, Poland, Pakistan, and the Philippines.
- We excluded ICD9_BTL data sources from both male and female models because they were producing implausibly high estimates compared to ICD9_detail and ICD10_detail data sources.
- We changed the lower bound of age-restrictions for death estimations from 1 year to 0 days. The upper bound remained the same at 95+ years.

- We changed the direction of the Socio-demographic Index covariate from 0 to -1.

The following table has the full list of covariates used for fatal inguinal, femoral, and abdominal hernia.

Table 1. Covariates used in inguinal, femoral, and abdominal hernia mortality modelling

Level	Covariate	Direction
1	BMI (mean)	-
	Cumulative cigarettes (10 years)	+
	Cumulative cigarettes (5 years)	+
	Smoking prevalence	+
2	Healthcare Access and Quality Index	-
3	Socio-demographic Index	-
	Education (years per capita)	-
	Log LDI ($I per capita)	-

Gallbladder and biliary diseases

Flowchart

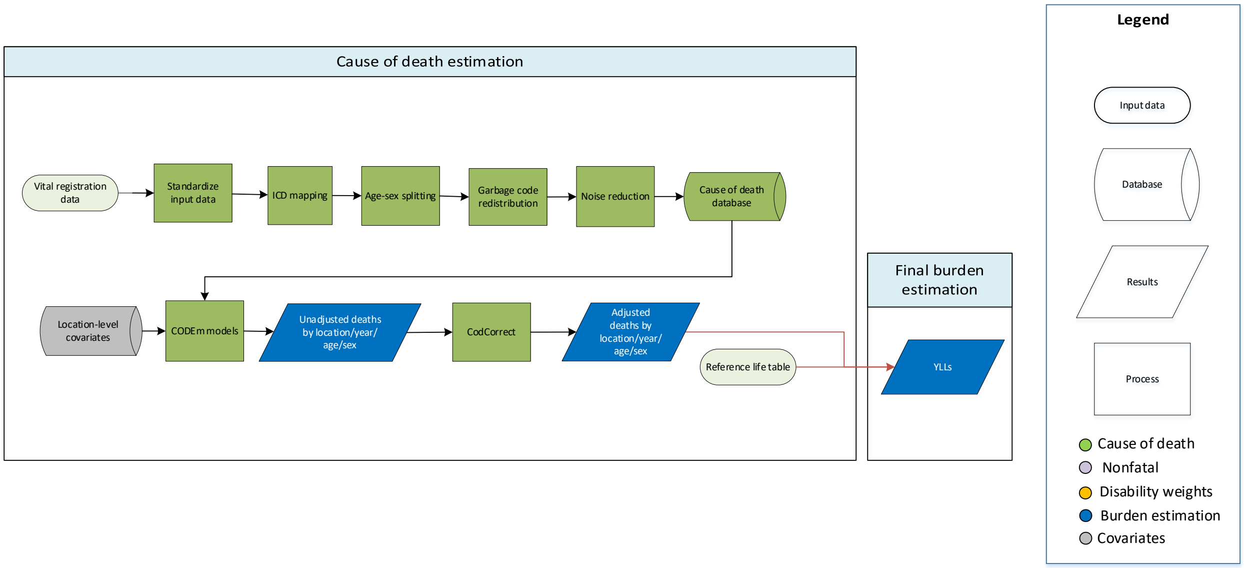

Input data

Data used to estimate mortality of gallbladder and biliary diseases consisted of vital registration data from the cause of death (COD) database. Outliers were identified by systematic examination of datapoints for all location-years. Specifically, we marked data as outliers in instances where garbage code redistribution and noise reduction, in combination with small sample sizes, resulted in unreasonable cause fractions. We also marked as outliers those data that violated well-established time or age trends.

Modelling strategy

The estimation strategy used for fatal gallbladder and biliary diseases is largely similar to methods used in GBD 2017. A standard CODEm model with location-level covariates was used to model deaths due to gallbladder and biliary diseases with age-restrictions for death estimations of 1 year for lower bound and 95+ years for upper bound (see appendix section on CODEm method for details). Separate models were conducted for male and female mortality. We then hybridised separate global and data-rich models to acquire unadjusted results, which we finalised and adjusted using CodCorrect to reach final YLLs due to gallbladder and biliary diseases.

Key changes from GBD 2017

- We added estimates for the following new locations: Monaco, San Marino, Palau, and Saint Kitts and Nevis.
- We added subnational location data for the following: Italy and Poland.
- We changed the direction of Socio-demographic Index and lag-distributed income covariates from 0 to -1 in GBD 2019.
- We replaced adjusted dietary covariates with age-sex-specific scaled exposure variable covariates with a direction of 1.

The following table has the full list of covariates used for fatal gallbladder and biliary diseases.

Table 1. Covariates used in gallbladder and biliary diseases mortality modelling

Level	Covariate	Direction
1	Age-sex-specific scaled exposure variable for low polyunsaturated fatty acids	+
	BMI (mean)	+
2	Alcohol (litres per capita)	+
	Healthcare Access and Quality Index	-
	Age-sex-specific scaled exposure variable for high red meat consumption	+
	Population over 65 (proportion)	+
3	Socio-demographic Index	-
	Education (years per capita)	-
	Log LDI ($I per capita)	-

Idiopathic Epilepsy

Flowchart

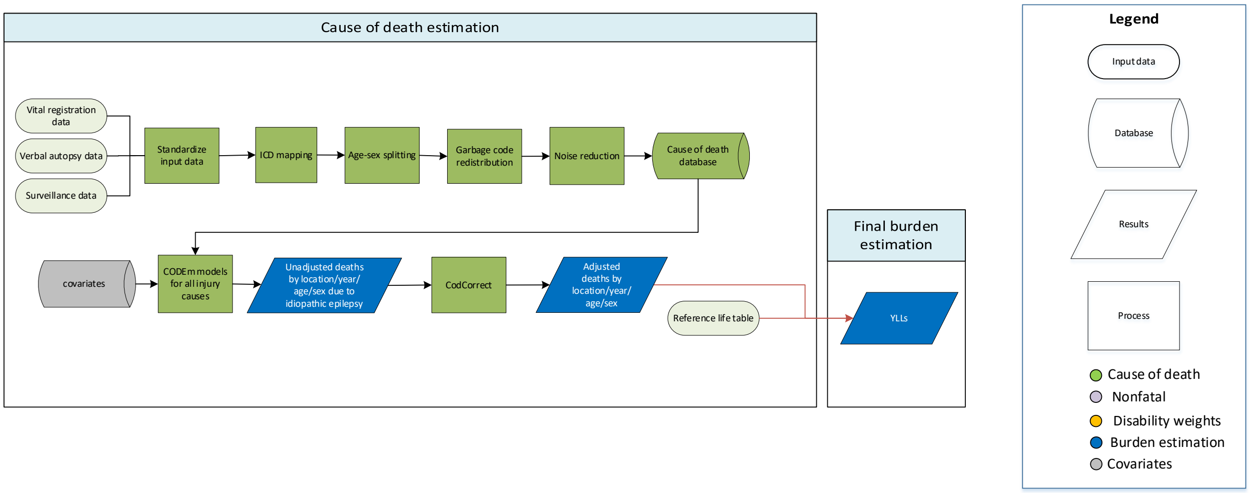

Input Data and Methodological Summary for Idiopathic Epilepsy

Input data

Data used to estimate epilepsy mortality included vital registration (VR), verbal autopsy, and China mortality surveillance data from the cause of death (COD) database. Our outlier criteria were to exclude data points that were (1) implausibly high or low relative to global or regional patterns, (2) substantially conflicted with established age or temporal patterns, or (3) substantially conflicted with other data sources based from the same locations or locations with similar characteristics (i.e., socio-demographic index).

Modeling strategy

The standard CODEm modelling approach (detailed in a appendix section 3.1) was used to estimate deaths due to idiopathic epilepsy. Separate models were conducted for male and female mortality, and the age range for both models was 28 days – 95+ years. Changes to these models relative to GBD 2017, and the complete list of covariates used in GBD 2019 are displayed below. Unadjusted death estimates were adjusted using CoDCorrect to produce final estimates of YLLs.

Key Changes from GBD 2017

- Introduction of subnational location data for Italy, Poland, Pakistan, the Philippines, and Nigeria.
- Introduction of the following new locations: Monaco, San Marino, Cook Islands, Nauru, Niue, Palau, Tokelau, Tuvalu, Monaco, San Marino, St Kitts, and Nevis.
- Changes in covariate choices. A covariate for pig meat consumption (kcal per capita) used in GBD 2017 was not modeled for use in CODEm in GBD 2019. All other covariates remained from GBD2017 (see Table 1).

Table 1. Covariates used in Idiopathic Epilepsy mortality modelling

Level	Covariate	Direction
1	Pigs (per capita)	+
	SEV scalar: epilepsy	+
	Mean systolic blood pressure (mmHg)	+
2	Health access and quality index	-
	Mean body mass index	+
	Mean serum total cholesterol (mmol/L)	+
3	Cumulative cigarettes (10 years)	+
	Cumulative cigarettes (5 years)	+
	Education (years per capita)	-
	Log LDI (per capita)	-
	Socio-demographic Index	-

The following plots show the influence of each covariate on the four CODEm models (male global, male data rich, female global, and female data rich). A positive standardized beta (to the right) means that the covariate was associated with increased death. A negative standardized beta (to the left) means the covariate was associated with decreased death.

Male, global

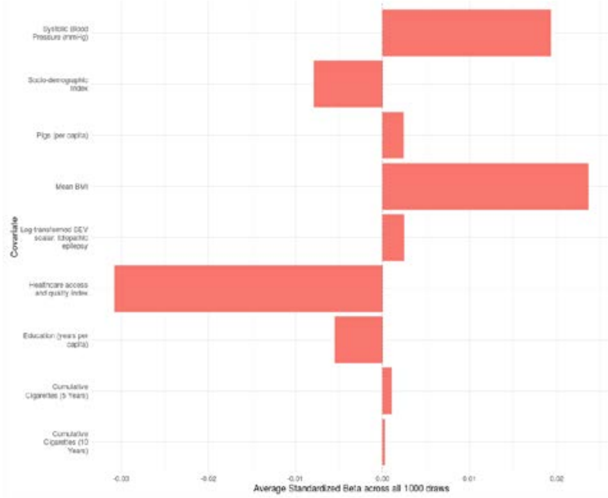

Male, data rich

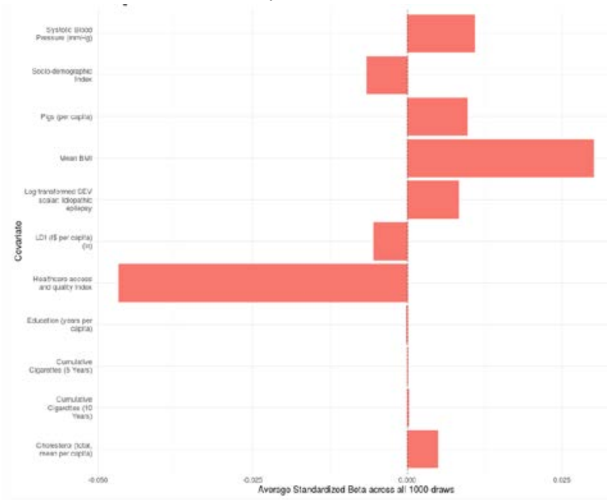

**Female, global**	**Female, data rich**
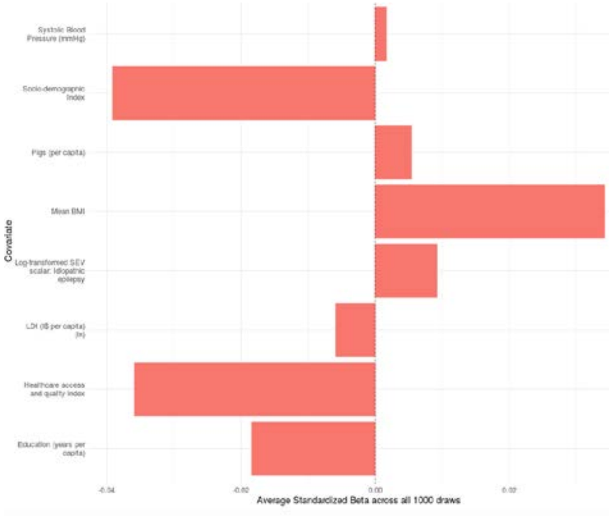	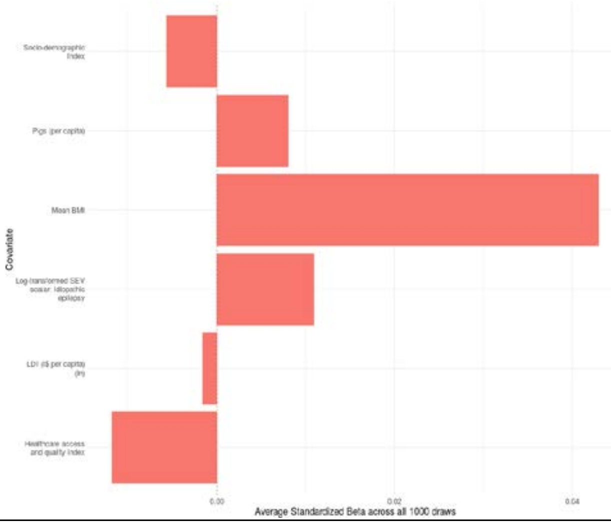

Diabetes Mellitus

Diabetes mellitus mortality was estimated for overall diabetes mellitus, diabetes mellitus type 1, and diabetes mellitus type 2 in GBD 2019.

Overall Diabetes Mellitus

Flowchart

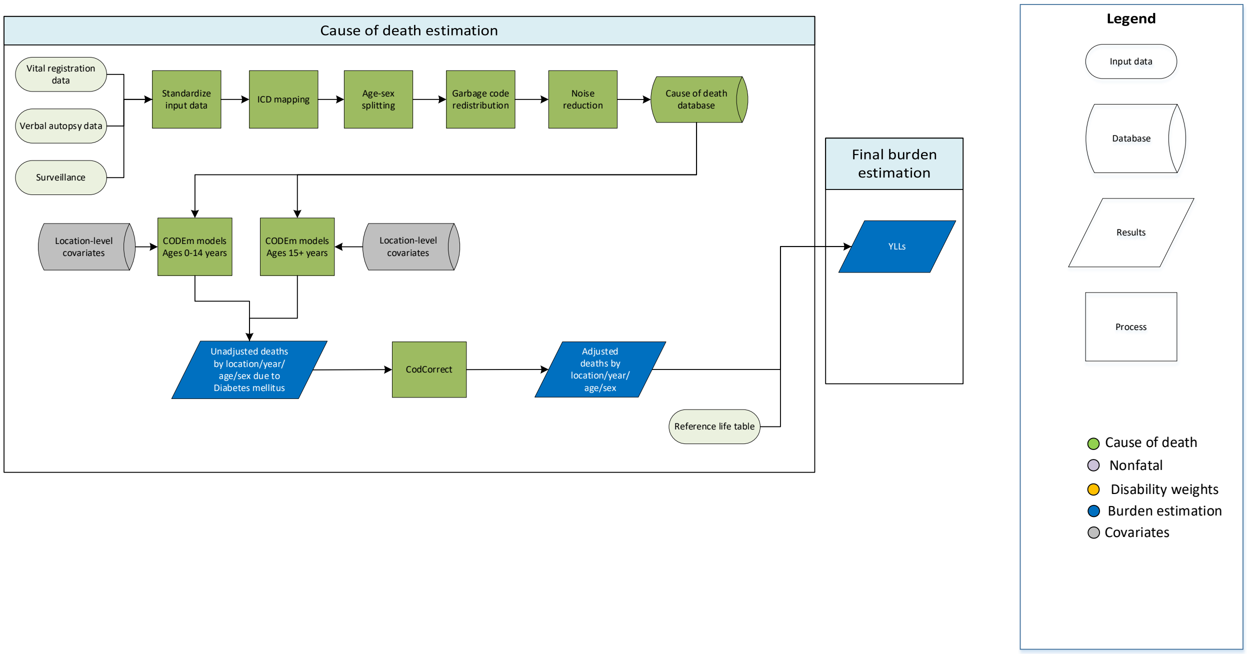

Input Data and Methodological Summary for diabetes mellitus

Input data

Overall diabetes mellitus mortality was estimated using deaths directly attributed to diabetes mellitus. We used verbal autopsy and vital registration data as inputs into the model.

Verbal autopsy data: We outliered data points from sources where there were zero deaths estimated in an age group as this was not realistic for deaths due to diabetes and we determined that these data sources were unreliable.

Vital registration data: We outliered all data from the India Medical Certification of Cause of Death report since the source of the data was unreliable according to expert opinion. We also outliered ICD9BTL data points that were inconsistent with the rest of the data series and created unlikely time trends.

Modelling strategy

The Cause of Death Ensemble model (CODEm) was used for deaths due to diabetes mellitus estimation.

In the overall diabetes mellitus model, we used two models to estimate overall diabetes deaths with different age restrictions. This is because deaths in younger age groups are almost exclusively due to type 1 diabetes, while deaths in older ages are primarily due to type 2 diabetes. This allowed us to select predictive covariates that are specific to the pathophysiology of diabetes type 1 and type 2. We set the younger age model from 0-14 years and the older age model from 15-95+ years. We determined the age threshold based on evidence of the onset age of diabetes type 2 occurring at younger ages.

Covariate selection

The following table lists the covariates included in the model. This requires that the covariate selected for the model must have the directional relationship with diabetes mellitus deaths. In GBD 2019, we made 2 updates. First, we changed 4 covariates to reflect the most current covariate available, proportion underweight to age-standardised underweight (weight-for-age) summary exposure variable, proportion stunting to age-standardised stunting (height-for-age) summary exposure variable, energy-adjusted grams of fruits to age- and sex-specific summary exposure variable for low fruit, and energy-adjusted grams of vegetables to age- and sex-specific summary exposure variable for low vegetables. Second, we selected a direction on covariates that we did not set a direction in previous GBD. We determined the direction based on the strength of the evidence.

Model	Level	Covariate	Direction
0-14 years	1	Healthcare access and quality index	-
	3	Education years per capita	-
	2	Age-standardised fertility rate	+
	2	Latitude	+
	2	Age-standardised underweight (weight-for-age) summary exposure variable	-
	2	Percentage of births occurring in women >35 years old	+
	2	Percentage of births occurring in women >40 years old	+
	3	Socio-demographic Index	-
	2	Age-standardised stunting (height-for-age) summary exposure variable	-
	2	Mean birth weight	-
15 + model	1	Age-standardised mean fasting plasma glucose (mmol/L)	+
	1	Age-standardised prevalence of diabetes	+
	3	Education years per capita	-
	3	Lag-distributed income per capita	+
	1	Mean BMI	+
	2	Mean cholesterol	+
	2	Mean systolic blood pressure	+
	1	Prevalence of obesity	+
	2	Age- and sex-specific summary exposure variable for low fruit	-
	2	Energy-adjusted grams of sugar	+

Model	Level	Covariate	Direction
	2	Age- and sex-specific summary exposure variable for low vegetables	-
	3	Healthcare access and quality index	-
	2	Age- and sex-specific summary exposure variable for alcohol use	+

Covariate Influences:

The following plots show the influence of each covariate on the four CODEm models (male global, male data rich, female global, and female data rich). A positive standardized beta (to the right) means that the covariate was associated with increased death. A negative standardized beta (to the left) means the covariate was associated with decreased death.

	**Data rich**	**Global**
Female 0-14 model	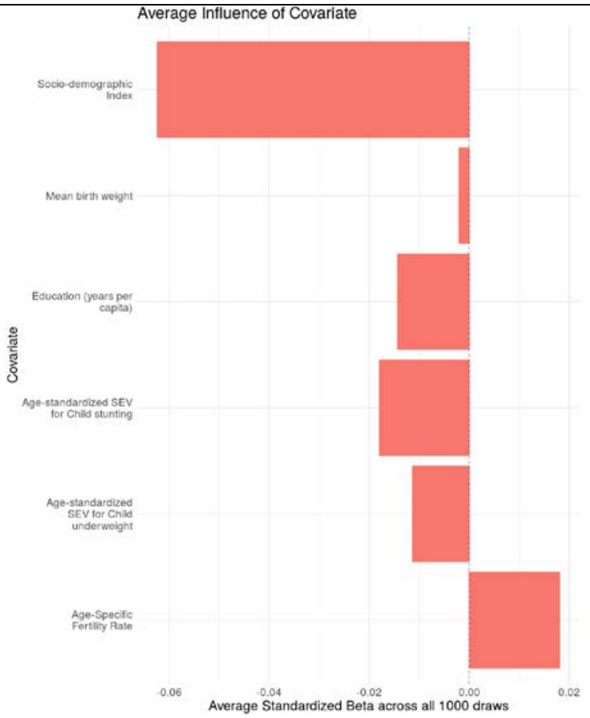	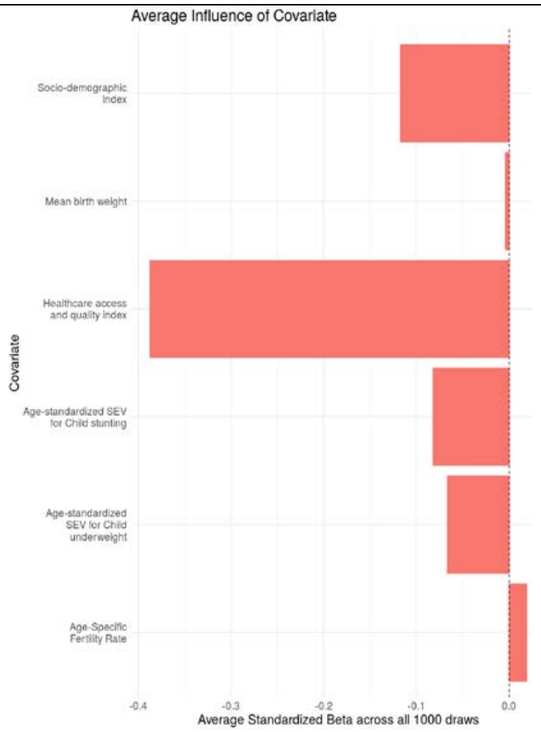

<table>
<tr><th></th><th>Data rich</th><th>Global</th></tr>
<tr><td>Male
0-14
model</td><td>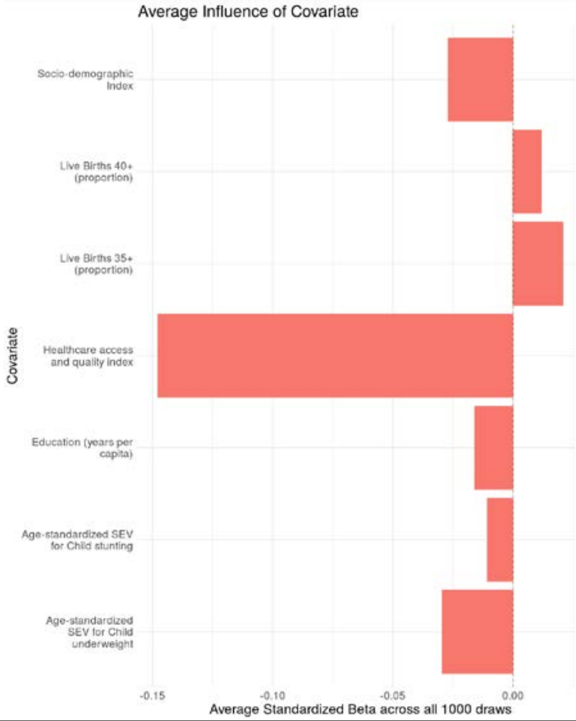
</td><td>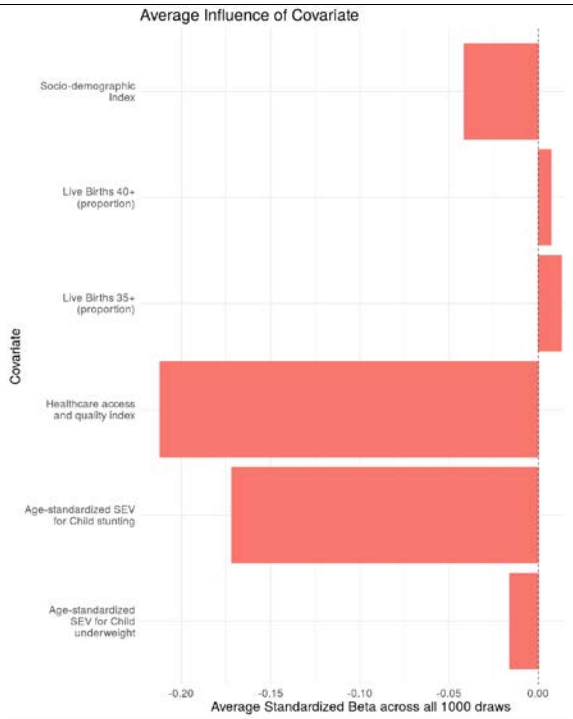
</td></tr>
<tr><td>Female
15+
model</td><td>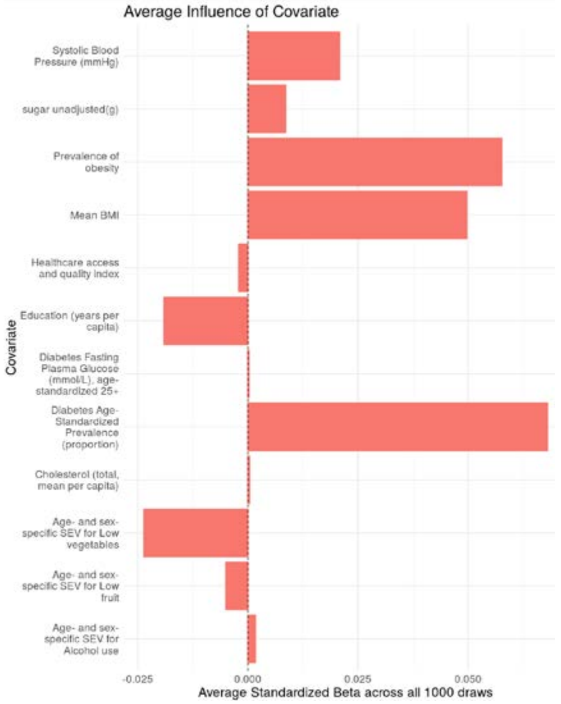
</td><td>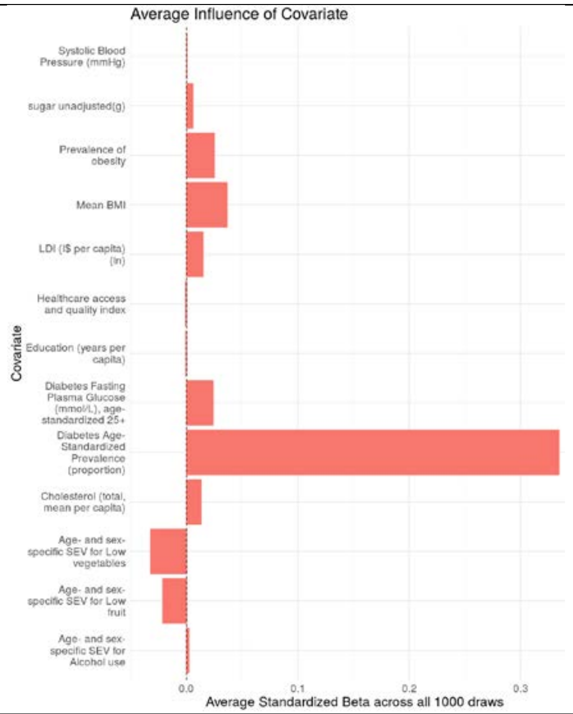
</td></tr>
</table>

	**Data rich**	**Global**
Male 15+ model	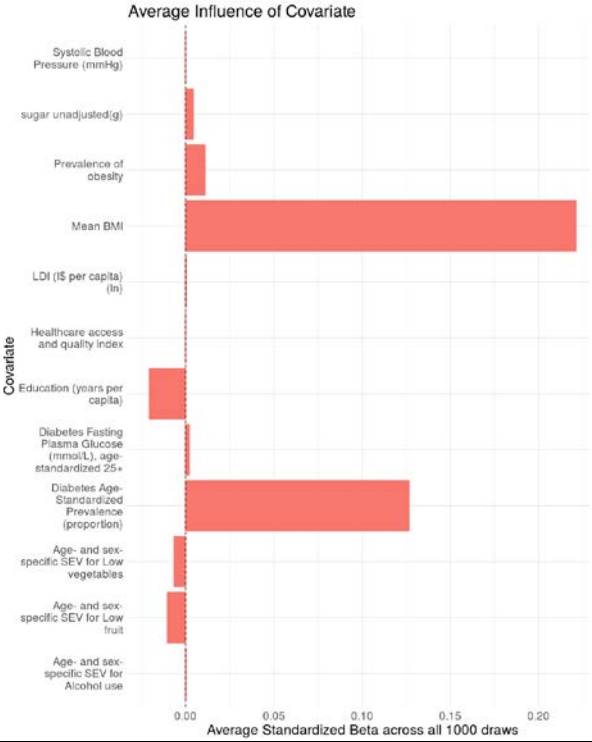	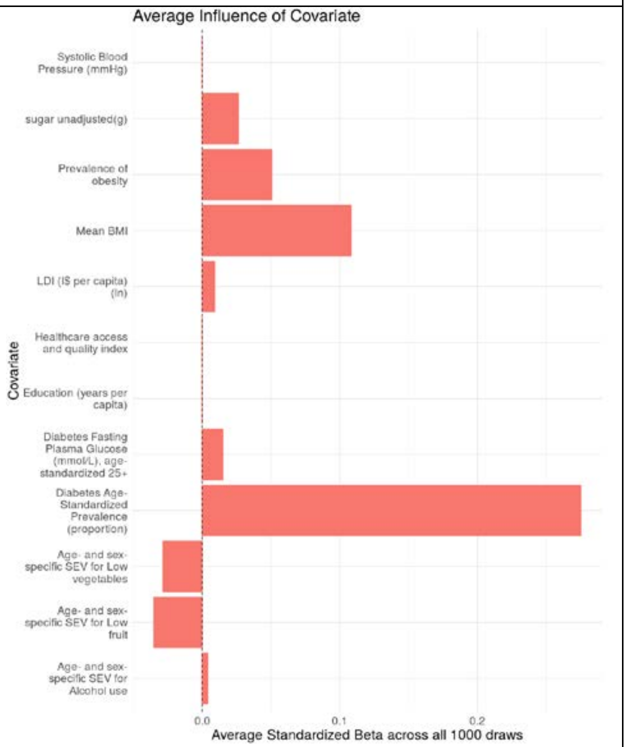

Chronic Kidney Disease

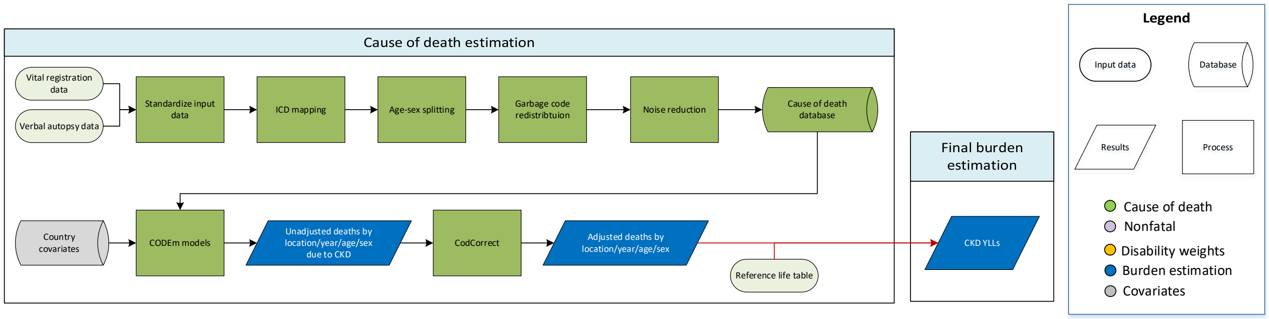

Input data

Vital registration and verbal autopsy data were used to model mortality due to chronic kidney disease. Data were standardised and mapped according to the GBD causes of death ICD mapping method. These data were then age-sex split, and appropriate redistribution of garbage code data was performed. Data points that violated well-established age or time trends or that resulted in extremely high or low cause fractions were marked as outliers and excluded.

Modelling strategy

The estimation strategy used for fatal chronic kidney disease is largely similar to methods used in GBD 2017. A standard CODEm model with location-level covariates was used to model deaths due to chronic kidney disease.

Key Changes from GBD 2017

- We removed the following covariates: whole grains per capita, animal fat per capita, and log lagged 10-year income per capita. We added lagged 10-year income per capita.
- Specified that CODem could only select covariates if the relationship detected between the covariate and mortality was in the direction known or suspected based on prior studies. This resulted the following changes: 1) SDI specified as having a negative association - previously not specified; 2) Red meat consumption specified with a positive association - previously not specified

The full list of covariates used in the GBD 2019 model are displayed below.

Level	Covariate	Direction
1	Diabetes fasting plasma glucose (mmol/L)	+
	Diabetes age-standardised prevalence (proportion)	+
	Mean systolic blood pressure (mmHg)	+
	Mean BMI	+
	Healthcare access and quality index	–
2	Mean cholesterol	+
	Total Calories available per capita per day	+
	Red meat unadjusted (kcal per capita)	+
3	Socio-demographic Index	–
	Education (years per capita)	–
	LDI (I$ per capita)	–

Covariate Influences:

The following plots show the influence of each covariate on the four CODEm models (male global, male data rich, female global, and female data rich). A positive standardized beta (to the right) means that the covariate was associated with increased death. A negative standardized beta (to the left) means the covariate was associated with decreased death.

Male Global

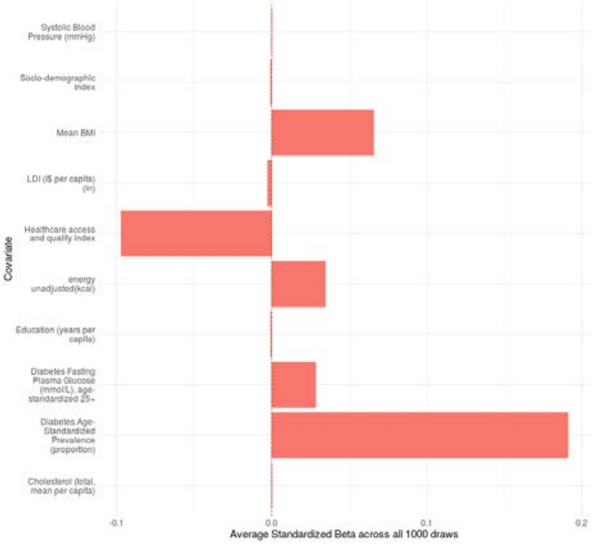

Male Data Rich

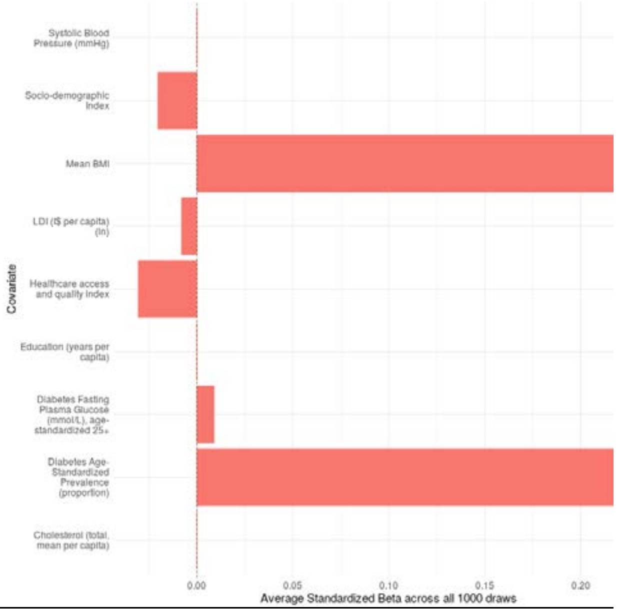

Female Global

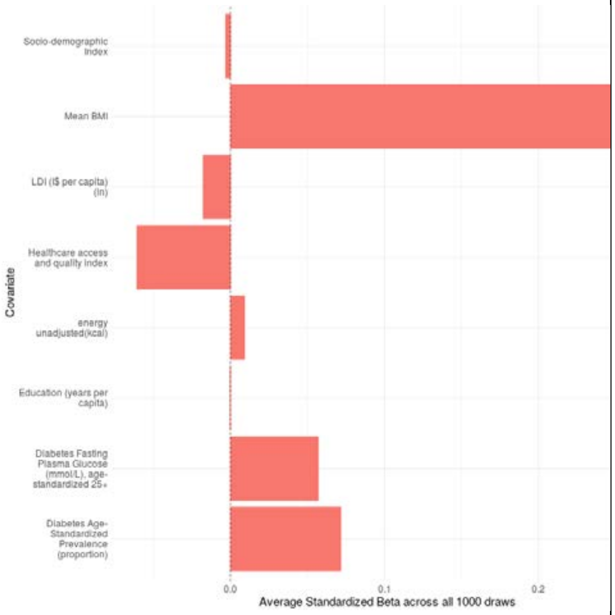

Female Data Rich

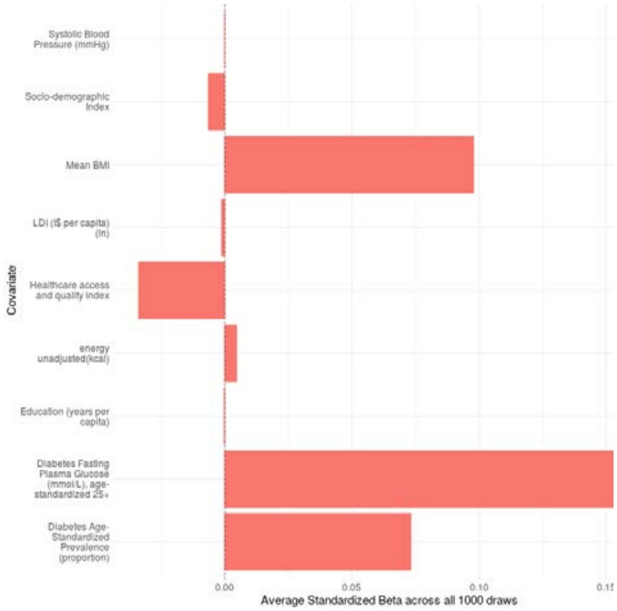

Congenital birth defects: neural tube defects, congenital heart anomalies, orofacial clefts, Down syndrome, Turner syndrome, Klinefelter syndrome, other chromosomal disorders, congenital musculoskeletal anomalies, urogenital congenital anomalies, digestive congenital anomalies, and other congenital birth defects.

Flowchart

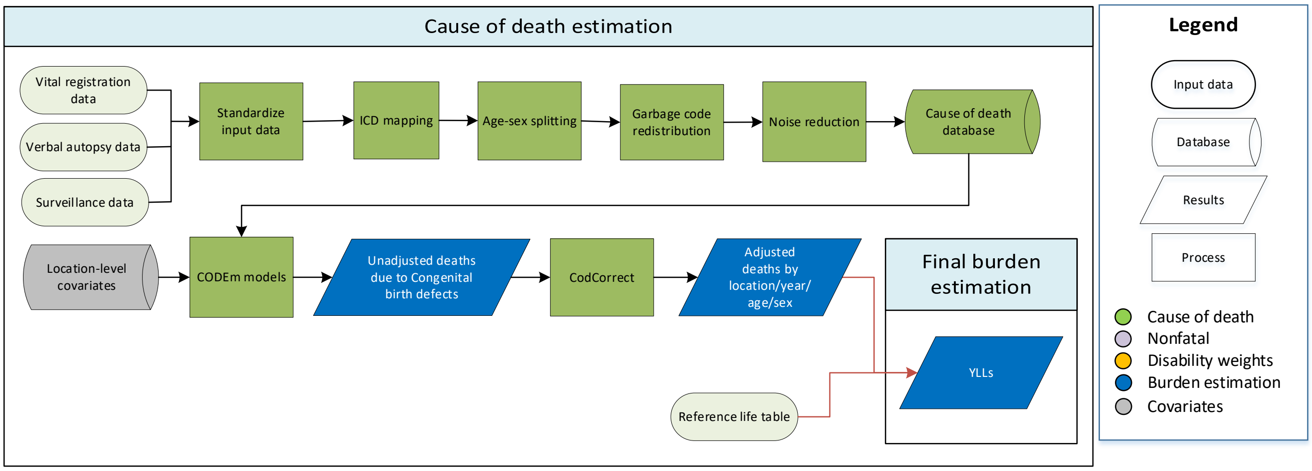

Input data

For GBD 2019, input data for estimating mortality due to congenital anomalies was centrally extracted, processed, and stored in cause of death (CoD) database. Vital registration (VR) was the dominant data type, followed by verbal autopsy (VA) and surveillance. Those CoD data sources that specified the subcause of birth defect were included in estimation of both the parent congenital anomalies model as well as in subtype-specific models.

For GBD 2019, data exclusions were limited. The majority of VA data were outliered in those over 5 years old as the age patterns were unreliable and led to poor model performance in the under-5 age groups. We also excluded some data sources from the parent model where only a subset of subcauses were specified (e.g., congenital heart disease, neural tube defects, and other congenital anomalies) and the sum of the subcauses clearly represented systematic underreporting of one of the subcauses. Systematic underreporting was suspected when sex- and age-specific rates were more than an order of magnitude lower than neighbouring or comparable locations. Data sources for those locations were still included by default for subcause specific models because underreporting of the total was not assumed to necessarily be associated with underreporting of all of the component conditions.

Modelling strategy

All types of congenital anomalies were estimated using cause of death ensemble modelling (CODEm) for GBD 2019, as was done for previous iterations of the GBD study. Specific causes included neural tube

defects, congenital heart anomalies, orofacial clefts, Down syndrome, other chromosomal anomalies, congenital musculoskeletal anomalies, urogenital congenital anomalies, digestive congenital anomalies, and other congenital birth defects. We assumed no mortality from either Klinefelter syndrome or Turner syndrome, for which we model nonfatal outcomes only. For GBD 2019, we modelled congenital anomalies as a cause of death for ages 0–69 years only, assuming that all mortality from congenital conditions occurs before age 70 years of age.

For GBD 2016, we added three new causes to the congenital anomalies: congenital musculoskeletal and limb anomalies; urogenital congenital anomalies; and digestive congenital anomalies. We made no additions to the causes of congenital anomalies for GBD 2017 or 2019.

Table 1: Covariates tested for CODEm model of overall congenital birth defects

Covariate	Transformation	Level	Direction
Maternal alcohol consumption during pregnancy (proportion)	None	1	+
In-facility delivery (proportion)	None	1	-
Live births 35+ (proportion)	None	1	+
Folic acid unadjusted (ug)	None	1	-
Folic acid fortification index	None	1	-
Birth prevalence of congenital heart disease	None	1	+
Birth prevalence of chromosomal anomalies	None	1	+
Legality of abortion	None	2	-
Antenatal care (1 visit) coverage (proportion)	None	2	-
Age-standardised summary exposure value (SEV) of smoking	None	2	+
Antenatal care (4 visits) coverage (proportion)	None	2	-
Healthcare Access and Quality Index	None	2	-
Maternal education (years per capita)	None	3	-
Alcohol (litres per capita)	None	3	+
Age-standardised SEV of low fruits	None	3	+
Outdoor air pollution (PM2.5)	None	3	+
Age-standardised SEV of household air pollution	None	3	+
Socio-demographic Index	None	3	-
Age-standardised SEV of low vegetables	None	3	+

Table 2: Covariates tested for CODEm model of neural tube defects

Covariate	Transformation	Level	Direction
In-facility delivery (proportion)	None	1	-
Folic acid unadjusted (ug)	None	1	-
Folic acid fortification index	None	1	-
Healthcare Access and Quality Index	None	2	-
Antenatal care (1 visit) coverage (proportion)	None	2	-
Antenatal care (4 visits) coverage (proportion)	None	2	-
Age-standardised SEV of smoking	None	2	+
Age-standardised SEV of low fruits	None	3	+
Age-standardised SEV of low vegetables	None	3	+
Maternal education (years per capita)	None	3	-
Socio-demographic Index	None	3	-
Legality of abortion	None	2	-
Maternal alcohol consumption during pregnancy (proportion)	None	3	+
Age-standardised SEV of household air pollution	None	3	+

Age-standardised SEV of fasting plasma glucose	None	3	+
Litres of alcohol consumed per capita	None	3	+

Table 3: Covariates selected for CODEm model of congenital heart anomalies

Covariate	Transformation	Level	Direction
Maternal alcohol consumption during pregnancy (proportion)	None	1	+
Birth prevalence of congenital heart disease	None	1	+
Socio-demographic Index	Log	2	-
Age-standardised SEV of smoking	None	2	+
Age-standardised SEV of diabetes	None	2	+
Healthcare Access and Quality Index	None	2	-
Legality of abortion	None	2	-
Antenatal care (1 visit) coverage (proportion)	None	2	-
In-facility delivery (proportion)	None	2	-
Maternal education (years per capita)	None	3	-
Alcohol (litres per capita)	None	3	+
Antenatal care (4 visits) coverage (proportion)	None	3	-
Skilled birth attendance (proportion)	None	3	-
Live births 35+ (proportion)	None	3	+

Table 4: Covariates selected for CODEm model of cleft lip and cleft palate

Covariate	Transformation	Level	Direction
Socio-demographic Index	None	1	-
Folic acid fortification index	None	1	-
Age-standardised SEV of diabetes	None	2	+
Maternal alcohol consumption during pregnancy (proportion)	None	2	+
Healthcare Access and Quality Index	None	2	-
Legality of abortion	None	2	-
Skilled birth attendance (proportion)	None	2	-
Age-standardised SEV of smoking	None	2	+
Age-standardised SEV of household air pollution	None	3	+
Age-standardised SEV of low vegetables	None	3	+
Alcohol (litres per capita)	None	3	+
Antenatal care (4 visits) coverage (proportion)	None	3	-
Maternal education (years per capita)	None	3	-
Age-standardised SEV of low fruits	None	3	+
Antenatal care (1 visit) coverage (proportion)	None	3	-

Table 5: Covariates selected for CODEm model of Down syndrome

Covariate	Transformation	Level	Direction
Live births 35+ (proportion)	None	1	+
Legality of abortion	None	1	-
Live births 40+ (proportion)	None	1	+
Birth prevalence of chromosomal anomalies	None	1	+
Socio-demographic Index	None	2	-
In-facility delivery (proportion)	None	2	-
Healthcare Access and Quality Index	None	2	-
Maternal alcohol consumption during pregnancy (proportion)	None	3	+
Antenatal care (1 visit) coverage (proportion)	None	3	-
Maternal education (years per capita)	None	3	-

Age-standardised SEV of household air pollution	None	3	+
Antenatal care (4 visits) coverage (proportion)	None	3	-
Age-standardised SEV of low vegetables	None	3	-
Age-standardised SEV of smoking	None	3	+
Litres of alcohol consumed per capita	None	3	+

Table 6: Covariates selected for CODEm model of other chromosomal abnormalities

Covariate	Transformation	Level	Direction
Live births 35+ (proportion)	None	1	+
Live births 40+ (proportion)	None	1	+
Legality of abortion	None	1	-
Lag distributed income (LDI) (I$ per capita)	Log	2	-
Healthcare Access and Quality Index	None	2	-
Antenatal care (4 visits) coverage (proportion)	None	2	-
Antenatal care (1 visit) coverage (proportion)	None	2	-
In-facility delivery (proportion)	None	2	-
Maternal alcohol consumption during pregnancy (proportion)	None	2	+
Socio-demographic Index	None	3	-
Alcohol (litres per capita)	None	3	+
Age-standardised SEV of smoking	None	3	+
Age-standardised SEV of household air pollution	None	3	+
Maternal education (years per capita)	None	3	-
Skilled birth attendance (proportion)	None	3	-

Table 7: Covariates selected for CODEm model of congenital musculoskeletal and limb anomalies

Covariate	Transformation	Level	Direction
Maternal alcohol consumption during pregnancy (proportion)	None	1	+
Legality of abortion	None	1	-
In-facility delivery (proportion)	None	2	-
Ag-standardised SEV of diabetes	None	2	+
Socio-demographic Index	None	2	-
Healthcare Access and Quality Index	None	2	-
Age-standardised SEV of household air pollution	None	2	+
Age-standardised SEV of smoking	None	2	+
Antenatal care (4 visits) coverage (proportion)	None	3	-
Alcohol (litres per capita)	None	3	+
Age-standardised SEV of low fruits	None	3	+
Age-standardised SEV of low vegetables	None	3	+
Maternal education (years per capita)	None	3	-
Antenatal care (1 visit) coverage (proportion)	None	3	-
LDI per capita	Log	3	-

Table 8: Covariates selected for CODEm model of urogenital congenital anomalies

Covariate	Transformation	Level	Direction
Age-standardised SEV of smoking	None	1	+
Maternal alcohol consumption during pregnancy (proportion)	None	1	+
Healthcare Access and Quality Index	None	2	-
Diabetes age-standardised prevalence (proportion)	None	2	+
Socio-demographic Index	None	2	-

Age-standardised SEV of outdoor air pollution	None	2	+
In-facility delivery (proportion)	None	2	-
Age-standardised SEV of household air pollution	None	2	+
Antenatal care (1 visit) coverage (proportion)	None	3	-
Alcohol (litres per capita)	None	3	+
Maternal education (years per capita)	None	3	-
LDI (I$ per capita)	Log	3	-
Antenatal care (4 visits) coverage (proportion)	None	3	-

Table 9: Covariates selected for CODEm model of digestive congenital anomalies

Covariate	Transformation	Level	Direction
Maternal alcohol consumption during pregnancy (proportion)	None	1	+
Age-standardised SEV of smoking	None	1	+
Age-standardised SEV of household air pollution	None	2	+
Diabetes age-standardised prevalence (proportion)	None	2	+
Age-standardised SEV of diabetes	None	2	+
Socio-demographic Index	None	2	-
Age-standardised SEV of obesity	None	2	+
In-facility delivery (proportion)	None	2	-
Healthcare Access and Quality Index	None	2	-
Alcohol (litres per capita)	None	3	+
Maternal education (years per capita)	None	3	-
Age-standardised SEV of low vegetables	None	3	+
Antenatal care (1 visit) coverage (proportion)	None	3	-
Antenatal care (4 visits) coverage (proportion)	None	3	-
Age-standardised SEV of low fruits	None	3	+
LDI (I$ per capita)	Log	3	-
MCI	None	3	-

Table 10: Covariates selected for CODEm model of other congenital birth defects

Covariate	Transformation	Level	Direction
Maternal alcohol consumption during pregnancy (proportion)	None	1	+
Live births 35+ (proportion)	None	1	+
Maternal education (years per capita)	None	2	-
Legality of abortion	None	2	-
In-facility delivery (proportion)	None	2	-
Age-standardised SEV of household air pollution	None	2	+
Healthcare Access and Quality Index	None	2	-
Antenatal care (1 visit) coverage (proportion)	None	3	-
Age-standardised SEV of diabetes	None	3	+
LDI (I$ per capita)	Log	3	-
Socio-demographic Index	None	3	-
Antenatal care (4 visits) coverage (proportion)	None	3	-
Alcohol (litres per capita)	None	3	+

Injuries

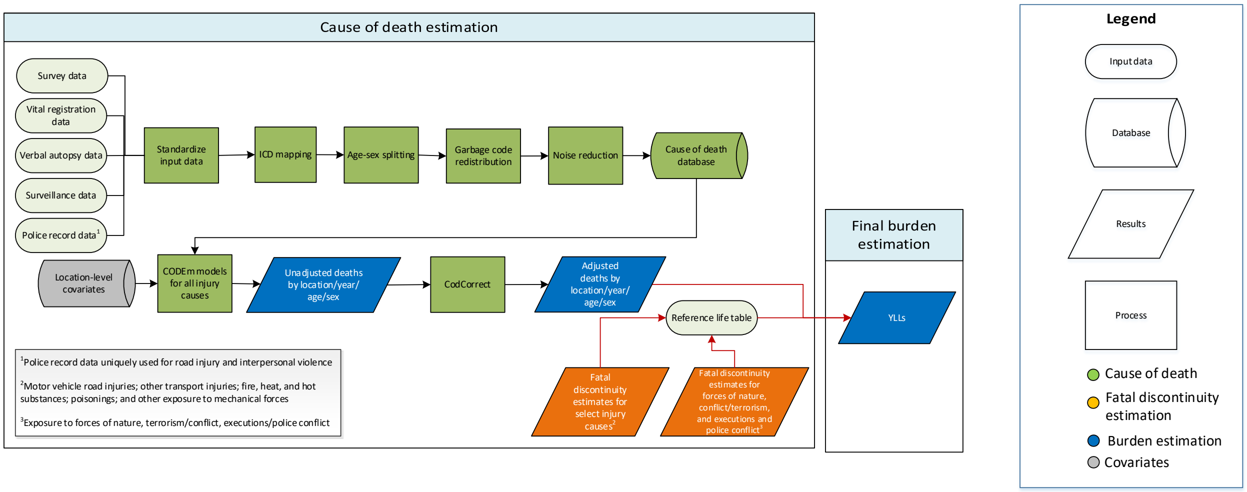

Input data

In GBD 2017, we estimated injury mortality from vital registration, verbal autopsy, mortality surveillance, censuses, surveys, and police record data. Police and crime reports were data sources uniquely used for the estimation of deaths from road traffic injury and interpersonal violence. The police data were collected from published studies, national agencies, and institutional surveys such as the United Nations Crime Trends Survey and the WHO Global Status Report on Road Safety Survey. For countries with vital registration data we did not use police records, except if the recorded number of road injury and interpersonal violence deaths from police records exceeded that in the vital registration.

Infrequently, data points were marked as outliers. Outlier criteria excluded data points that (1) were implausibly high or low relative to global or regional patterns, (2) substantially conflicted with established age or temporal patterns, or (3) significantly conflicted with other data sources conducted from the same locations or locations with similar characteristics (ie, Socio-demographic Index).

Modelling strategy

Overview

In GBD 2019, the standard CODEm modelling approach was applied to estimate deaths due to all causes of injury, excluding “Exposure to forces of nature,” and “Conflict and terrorism”. These causes were modelled solely outside of the CODEm process as fatal discontinuities estimation; this process is detailed further in the section on fatal discontinuities estimation in the appendix.

Fatal discontinuity was estimated for ten injury causes also modeled in CODEm. These causes included “Other transport injuries”, “Fire, heat, and hot substances”, “Poisoning by other means”, “Other exposure to mechanical forces”, “Non-venomous animal contact”, “Environmental heat and cold exposure”, “Physical violence by firearm”, “Physical violence by sharp object”, “Physical violence by other means”, “Executions and police conflict”. Final fatal discontinuity estimations for these causes were merged with CODEm results post-CoDCorrect to produce final cause of death results.

Refer to the table at the end of this section for a complete list of the cause-of-injury categories, modelling strategies, and covariate changes from GBD 2017.

GBD injury codes and categories

The International Classification of Diseases (ICD) was used to classify injuries. In GBD, injury incidence and death are defined as ICD-9 codes E000-E999 and ICD-10 chapters V to Y. There is one exception: deaths and cases of alcohol poisoning and drug overdoses are classified under drug and alcohol use disorders. In GBD 2019, injury causes were organized into 30 mutually exclusive and collectively exhaustive external cause-of-injury categories.

Preparation of data

The preparation of cause of death data includes age splitting, age-sex splitting, smoothing, and outlier detection. These steps are described in detail by Naghavi et al and Lozano et al.[1,2,3] The concept of "garbage codes" and redistribution of these codes was proposed in GBD 1990.[4] Garbage codes are causes of death that should not be identified as specific underlying causes of death but have been entered as the underlying cause of death on death certificates. A classic example of these types of codes in injuries chapters are "Exposure to unspecified factor" (X59 in ICD-10 and E887 in ICD-9) and all undetermined intent codes (Y10-Y34 in ICD-10 and E980-E988 in ICD-9). Other examples of garbage codes in injuries are the coding of an injury death to intermediate codes like septicemia or peritonitis or as an ill-defined and unknown cause of mortality (R99). Approximately 2% of total deaths in countries with vital registration data are assigned to these three injury garbage code categories.

Splitting into sublevel causes

In countries with non-detail ICD code data, cause-of-injury categories were proportionally split into sublevel cause-of-injury categories. The sublevel cause-of-injury causes were created in the CoDCorrect process. One of the countries with non-detail ICD code data is South Africa, and in GBD 2013 the proportions of sublevel cause-of-injury were based on vital registration data. For GBD iterations of 2015, 2016, 2017, and 2019, the proportions were based on post-mortem investigation of injury deaths as described in the paper by Matzopoulos et al. 2015.[5]

Limitations and model assumptions

We added police data for road injuries and interpersonal violence to help predict level and age patterns in countries with sparse or absent cause of death data even though we know from countries with near-complete vital registration data that police records tend to underestimate the true level of deaths. However, we applied police data estimates in instances where reported deaths were higher than vital registration numbers.

During GBD 2019, the input data for the US was reviewed for completeness, and we determined that the US National Vital Statistics System (NVSS) systematically underreports deaths due to police violence by about 50% every year. In order to quantify this bias, we ran a network meta-regression on NVSS data with direct comparisons by state and year to Mapping Police Violence (MPV), an alternate open-source database that we believe more accurately captures deaths due to police violence, and indirect comparisons to an additional source, Fatal Encounters (FE). The regression included a fixed effect on state to capture different underreporting rates across states, but assumed that underreporting rates are constant across age, sex, and year. Additionally, since MPV does not attempt to capture police killed by civilians and neither MPV nor FE attempt to capture executions, death counts from the FBI's Law

Enforcement Officers Killed and Assaulted database and the Death Penalty Information Center (DPIC) were added to these data sources in order to conform them to the GBD definition of executions and police conflict. We then used the underreporting rates estimated by the network meta-regression to scale the CODCorrect estimates for executions and police conflict in the United States upwards to a more accurate level. To maintain consistency with the all-cause mortality envelope, the deaths added to executions and police conflict were also removed proportionally from interpersonal violence and its relevant sub-causes. Record linkage between NVSS and open-source databases has shown that interpersonal violence is the most common underlying cause of death listed on death certificates for mis-assigned police violence deaths.[6]

Covariates

The following covariates were included.

**Transport Injuries**		
Level	Covariate	Direction
1	BAC law professional drivers (quartile)	1
1	BAC law general population (quartile)	1
1	BAC law youth drivers (quartile)	1
1	Liters of alcohol consumed per capita	1
1	Speed limit law rural (quartile)	1
1	Speed limit law urban (quartile)	1
1	Vehicles - 2 wheels fraction (proportion)	1
1	Vehicles - 2+4 wheels (per capita)	1
2	Education (years per capita)	-1
2	Healthcare access and quality index	-1
2	LDI (I$ per capita)	-1
2	Population 15 to 30 (proportion)	1
2	Population Density (300-500 ppl/sqkm, proportion)	1
2	Population Density (500-1000 ppl/sqkm, proportion)	1
2	Population-weighted mean temperature	1
2	Socio-demographic Index	-1
3	Rainfall Quintile 5 (proportion)	1
**Road injuries**		
Level	Covariate	Direction
1[a]	BAC law professional drivers (quartile)	1
1[a]	BAC law general population (quartile)	1
1[a]	BAC law youth drivers (quartile)	1
1	Liters of alcohol consumed per capita	1
1	Log-transformed SEV scalar: Road Inj	1
1[a]	Speed limit law rural (quartile)	1
1[a]	Speed limit law urban (quartile)	1
1	Vehicles - 2 wheels (per capita)	1

1	Vehicles - 2 wheels fraction (proportion)	1
1	Vehicles - 2+4 wheels (per capita)	1
1	Vehicles - 4 wheels (per capita)	1
2[b]	Education (years per capita)	-1
2	Healthcare access and quality index	-1
2[b]	LDI (I$ per capita)	-1
2	Population 15 to 30 (proportion)	1
2	Population Density (300-500 ppl/sqkm, proportion)	1
2	Population Density (500-1000 ppl/sqkm, proportion)	1
2	Population-weighted mean temperature	1
2[b]	Socio-demographic Index	-1
3	Rainfall Quintile 5 (proportion)	1
**Pedestrian road injuries**		
Level	Covariate	Direction
1	BAC law professional drivers (quartile)	1
1	BAC law general population (quartile)	1
1	BAC law youth drivers (quartile)	1
1	Liters of alcohol consumed per capita	1
1	Log-transformed SEV scalar: Pedest	1
1	Speed limit law rural (quartile)	1
1	Speed limit law urban (quartile)	1
1	Vehicles - 2 wheels fraction (proportion)	1
1	Vehicles - 2+4 wheels (per capita)	1
2	Education (years per capita)	-1
2	Healthcare access and quality index	-1
2	LDI (I$ per capita)	-1
2	Population 15 to 30 (proportion)	1
2	Population Density (300-500 ppl/sqkm, proportion)	1
2	Population Density (500-1000 ppl/sqkm, proportion)	1
2[c]	Population-weighted mean temperature	1
2	Socio-demographic Index	-1
3	Rainfall Quintile 5 (proportion)	1
**Pedestrian road injuries**		
Level	Covariate	Direction
1	BAC law professional drivers (quartile)	1
1	BAC law general population (quartile)	1
1	BAC law youth drivers (quartile)	1
1	Liters of alcohol consumed per capita	1
1	Log-transformed SEV scalar: Cyclist	1
1	Speed limit law rural (quartile)	1

1	Speed limit law urban (quartile)	1
1	Vehicles - 2 wheels fraction (proportion)	1
1	Vehicles - 2+4 wheels (per capita)	1
2	Education (years per capita)	-1
2	Healthcare access and quality index	-1
2	LDI (I$ per capita)	-1
2	Population 15 to 30 (proportion)	1
2	Population Density (300-500 ppl/sqkm, proportion)	1
2	Population Density (500-1000 ppl/sqkm, proportion)	1
2	Population-weighted mean temperature	1
2	Socio-demographic Index	-1
3	Rainfall Quintile 5 (proportion)	1
**Motorcyclist road injuries**		
Level	Covariate	Direction
1	BAC law professional drivers (quartile)	1
1	BAC law general population (quartile)	1
1	BAC law youth drivers (quartile)	1
1	Liters of alcohol consumed per capita	1
1	Log-transformed SEV scalar: Mot Cyc	1
1	Speed limit law rural (quartile)	1
1	Speed limit law urban (quartile)	1
1[d]	Vehicles - 2 wheels fraction (proportion)	1
2	Education (years per capita)	-1
2	Healthcare access and quality index	-1
2	LDI (I$ per capita)	-1
2	Population 15 to 30 (proportion)	1
2	Population Density (300-500 ppl/sqkm, proportion)	1
2	Population Density (500-1000 ppl/sqkm, proportion)	1
2	Population-weighted mean temperature	1
2	Socio-demographic Index	-1
3	Rainfall Quintile 5 (proportion)	1
**Motor vehicle road injuries**		
Level	Covariate	Direction
1	BAC law professional drivers (quartile)	1
1	BAC law general population (quartile)	1
1	BAC law youth drivers (quartile)	1
1	Liters of alcohol consumed per capita	1
1	Log-transformed SEV scalar: Mot Veh	1
1	Speed limit law rural (quartile)	1
1	Speed limit law urban (quartile)	1

1	Vehicles - 4 wheels (per capita)	1
2	Education (years per capita)	-1
2	Healthcare access and quality index	-1
2	LDI (I$ per capita)	-1
2	Population 15 to 30 (proportion)	1
2	Population Density (300-500 ppl/sqkm, proportion)	1
2	Population Density (500-1000 ppl/sqkm, proportion)	1
2	Population-weighted mean temperature	1
2	Socio-demographic Index	-1
3	Rainfall Quintile 5 (proportion)	1
**Other road injuries**		
Level	Covariate	Direction
1	BAC law professional drivers (quartile)	1
1	BAC law general population (quartile)	1
1	BAC law youth drivers (quartile)	1
1	Liters of alcohol consumed per capita	1
1	Log-transformed SEV scalar: Oth Road	1
1	Speed limit law rural (quartile)	1
1	Speed limit law urban (quartile)	1
1	Vehicles - 2 wheels fraction (proportion)	1
1	Vehicles - 2+4 wheels (per capita)	1
2	Education (years per capita)	-1
2	Healthcare access and quality index	-1
2	LDI (I$ per capita)	-1
2	Population 15 to 30 (proportion)	1
2	Population-weighted mean temperature	1
3	Rainfall Quintile 5 (proportion)	1
3[e]	Socio-demographic Index	-1
**Other transport injuries**		
Level	Covariate	Direction
1	BAC law professional drivers (quartile)	1
1	BAC law general population (quartile)	1
1	BAC law youth drivers (quartile)	1
1	Liters of alcohol consumed per capita	1
1	Log-transformed SEV scalar: Oth Trans	1
1	Speed limit law rural (quartile)	1
1	Speed limit law urban (quartile)	1
1[f]	Vehicles - 2 wheels fraction (proportion)	1
1	Vehicles - 2+4 wheels (per capita)	1
2	Education (years per capita)	-1
2	Healthcare access and quality index	-1

2	LDI (I$ per capita)	-1
2	Population 15 to 30 (proportion)	1
2	Population Density (300-500 ppl/sqkm, proportion)	1
2	Population Density (500-1000 ppl/sqkm, proportion)	1
2	Population-weighted mean temperature	1
2	Socio-demographic Index	-1
3	Rainfall Quintile 5 (proportion)	1
**Falls**		
Level	Covariate	Direction
1	Education (years per capita)	-1
1	Liters of alcohol consumed per capita	1
1	Log-transformed SEV scalar: Falls	1
2	Healthcare access and quality index	-1
2	Population-weighted mean temperature	-1
3	Elevation Over 1500m (proportion)	1
3	LDI (I$ per capita)	-1
3	Socio-demographic Index	-1
**Drowning**		
Level	Covariate	Direction
1	Coastal Population within 10km (proportion)	1
1	Landlocked Nation (binary)	-1
1	Log-transformed SEV scalar: Drown	1
1	Population-weighted mean temperature	1
1	Rainfall Quintile 1 (proportion)	-1
1	Rainfall Quintile 5 (proportion)	1
2	Elevation Under 100m (proportion)	1
3	Education (years per capita)	-1
3	LDI (I$ per capita)	-1
3	Socio-demographic Index	-1
**Fire, heat, and hot substances**		
Level	Covariate	Direction
1	Log-transformed SEV scalar: Fire	1
1	Population-weighted mean temperature	1
2	Healthcare access and quality index	-1
2	Indoor Air Pollution (All Cooking Fuels)	1
2	Population Density (over 1000 ppl/sqkm, proportion)	1
2	Tobacco (cigarettes per capita)	1
3	Education (years per capita)	-1
3	LDI (I$ per capita)	-1
3	Socio-demographic Index	-1

Poisonings		
Level	Covariate	Direction
1	Log-transformed SEV scalar: Poison	1
1	Opium Cultivation (binary)	1
1	Population-weighted mean temperature	1
2	Healthcare access and quality index	-1
2	Population Density (over 1000 ppl/sqkm, proportion)	-1
2	Population Density (under 150 ppl/sqkm, proportion)	1
3	Education (years per capita)	-1
3	LDI (I$ per capita)	-1
3	Socio-demographic Index	-1
**Poisoning by carbon monoxide**		
Level	Covariate	Direction
1	Log-transformed SEV scalar: Inj Pois CO	1
2	Population-weighted mean temperature	-1
3	Education (years per capita)	-1
3	Healthcare access and quality index	-1
3	LDI (I$ per capita)	-1
3	Socio-demographic Index	-1
**Poisoning by other means**		
Level	Covariate	Direction
1	Log-transformed SEV scalar: Inj Pois Oth	1
1	Population-weighted mean temperature	1
3	Education (years per capita)	-1
3	Healthcare access and quality index	-1
3	LDI (I$ per capita)	-1
3	Socio-demographic Index	-1
**Exposure to mechanical forces**		
Level	Covariate	Direction
1	Population-weighted mean temperature	1
2	Healthcare access and quality index	-1
2	Population Density (over 1000 ppl/sqkm, proportion)	-1
2	Population Density (under 150 ppl/sqkm, proportion)	1
3	Education (years per capita)	-1
3	LDI (I$ per capita)	-1
3	Socio-demographic Index	-1
**Unintentional firearm injuries**		
Level	Covariate	Direction
1	Log-transformed SEV scalar: Mech Gun	1
1	Population-weighted mean temperature	1

2	Healthcare access and quality index	-1
3	Education (years per capita)	-1
3	LDI (I$ per capita)	-1
3	Population Density (over 1000 ppl/sqkm, proportion)	-1
3	Population Density (under 150 ppl/sqkm, proportion)	1
3	Socio-demographic Index	-1
**Other exposure to mechanical forces**		
Level	Covariate	Direction
1	Log-transformed SEV scalar: Oth Mech	1
1	Population-weighted mean temperature	1
2	Healthcare access and quality index	-1
2	Population Density (over 1000 ppl/sqkm, proportion)	-1
2	Population Density (under 150 ppl/sqkm, proportion)	1
3	Education (years per capita)	-1
3	LDI (I$ per capita)	-1
3	Socio-demographic Index	-1
**Adverse effects of medical treatment**		
Level	Covariate	Direction
1	Education (years per capita)	-1
1[g]	Liters of alcohol consumed per capita	1
1	Population-weighted mean temperature	1
2	Healthcare access and quality index	-1
3	LDI (I$ per capita)	1
3	Socio-demographic Index	-1
**Environmental heat and cold exposure**		
Level	Covariate	Direction
2	Healthcare access and quality index	-1
3	90th percentile climatic temperature in the given country-year.	1
3	Education (years per capita)	-1
3	Elevation 500 to 1500m (proportion)	1
3	Elevation Over 1500m (proportion)	1
3	LDI (I$ per capita)	-1
3	Population Density (150-300 ppl/sqkm, proportion)	-1
3	Population-weighted mean temperature	1
3	Rainfall (Quintiles 4-5)	1
3	Sanitation (proportion with access)	-1
3	Socio-demographic Index	-1
**Animal contact**		

Level	Covariate	Direction
1	Liters of alcohol consumed per capita	1
1	Log-transformed SEV scalar: Animal	1
1	Population-weighted mean temperature	1
2	Healthcare access and quality index	-1
2	Population 15 to 30 (proportion)	1
3	Education (years per capita)	-1
3	Elevation Over 1500m (proportion)	-1
3	Elevation Under 100m (proportion)	1
3	LDI (I$ per capita)	-1
3	Population Density (over 1000 ppl/sqkm, proportion)	-1
3	Population Density (under 150 ppl/sqkm, proportion)	1
3	Socio-demographic Index	-1
**Venomous animal contact**		
Level	Covariate	Direction
1	Liters of alcohol consumed per capita	1
1	Log-transformed SEV scalar: Venom	1
1	Absolute value of average latitude	-1
1	Liters of alcohol consumed per capita	1
1	Mean number of venomous snake species	1
1	Proportion of population vulnerable to snake species	1
1	Population-weighted mean temperature	1
1	Rainfall population-weighted (mm/yr)	1
1	Proportion of population involved in agricultural activities	1
1	Sahel Region of Africa (binary)	1
1	Urbanicity	-1
2	Healthcare access and quality index	-1
3	Education (years per capita)	-1
3	Elevation Over 1500m (proportion)	-1
3	Elevation Under 100m (proportion)	-1
3	LDI (I$ per capita)	-1
3	Population Density (over 1000 ppl/sqkm, proportion)	-1
3	Population Density (under 150 ppl/sqkm, proportion)	1
3	Socio-demographic Index	-1
**Non-venomous animal contact**		
Level	Covariate	Direction
1[k]	Elevation Over 1500m (proportion)	-1
1[k]	Elevation Under 100m (proportion)	1

1	Liters of alcohol consumed per capita	1
1	Log-transformed SEV scalar: Non Ven	1
1	Population-weighted mean temperature	1
2[l]	Healthcare access and quality index	-1
3	Education (years per capita)	-1
3[m]	Elevation Over 1500m (proportion)	-1
3[m]	Elevation Under 100m (proportion)	1
3	LDI (I$ per capita)	-1
3[m]	Population Density (over 1000 ppl/sqkm, proportion)	-1
3[m]	Population Density (under 150 ppl/sqkm, proportion)	1
3	Socio-demographic Index	-1
**Foreign body**		
Level	Covariate	Direction
1	Education (years per capita)	1
1	Indoor Air Pollution (All Cooking Fuels)	1
1	LDI (I$ per capita)	1
1	Liters of alcohol consumed per capita	1
1	Population Over 65 (proportion)	1
1	Population-weighted mean temperature	1
2	Healthcare access and quality index	-1
3	Socio-demographic Index	-1
**Pulmonary aspiration and foreign body in airway**		
Level	Covariate	Direction
1[n]	Education (years per capita)	-1
1	Liters of alcohol consumed per capita	1
1	Log-transformed SEV scalar: F Body Asp	1
1	Population-weighted mean temperature	1
2[o]	Alcohol binge drinker proportion, age-standardized	1
2	Healthcare access and quality index	-1
2	Mean BMI	1
3	LDI (I$ per capita)	-1
3	Socio-demographic Index	-1
**Foreign body in other body part**		
Level	Covariate	Direction
1	Liters of alcohol consumed per capita	1
1	Log-transformed SEV scalar: Oth F Body	1
1	Population-weighted mean temperature	1
2	Healthcare access and quality index	-1
3	Education (years per capita)	-1
3	LDI (I$ per capita)	-1

3	Socio-demographic Index	-1
**Other unintentional injuries**		
Level	Covariate	Direction
1	Liters of alcohol consumed per capita	1
1	Log-transformed SEV scalar: Oth Unint	1
1	Population-weighted mean temperature	1
1	Vehicles - 2 wheels (per capita)	1
1	Vehicles - 4 wheels (per capita)	1
2	Healthcare access and quality index	-1
3	Education (years per capita)	-1
3	LDI (I$ per capita)	-1
3	Population Density (over 1000 ppl/sqkm, proportion)	-1
3	Population Density (under 150 ppl/sqkm, proportion)	1
3	Socio-demographic Index	-1
**Self-harm**		
Level	Covariate	Direction
1	12-month non-partner sexual violence	1
1	Liters of alcohol consumed per capita	1
1[h]	Log-transformed SEV scalar: Self Harm	1
1	Major depressive disorder	1
1[i]	Muslim Religion (proportion of population)	1
1	Population-weighted mean temperature	1
2	Healthcare access and quality index	-1
2	Population Density (150-300 ppl/sqkm, proportion)	1
2	Population Density (300-500 ppl/sqkm, proportion)	-1
2	Population Density (500-1000 ppl/sqkm, proportion)	-1
2	Population Density (over 1000 ppl/sqkm, proportion)	-1
2	Population Density (under 150 ppl/sqkm, proportion)	1
3	Education (years per capita)	-1
3	LDI (I$ per capita)	-1
3	Socio-demographic Index	-1
**Self-harm by firearm**		
Level	Covariate	Direction
1	12-month non-partner sexual violence	1
1	Liters of alcohol consumed per capita	1
1	Log-transformed SEV scalar: Self Harm	1

1	Major depressive disorder	1
1	Population-weighted mean temperature	1
2	Healthcare access and quality index	-1
2	Population Density (150-300 ppl/sqkm, proportion)	1
2	Population Density (300-500 ppl/sqkm, proportion)	-1
2	Population Density (500-1000 ppl/sqkm, proportion)	-1
2	Population Density (over 1000 ppl/sqkm, proportion)	-1
2	Population Density (under 150 ppl/sqkm, proportion)	1
3	Education (years per capita)	-1
3	LDI (I$ per capita)	-1
3	Socio-demographic Index	-1
**Self-harm by other specified means**		
Level	Covariate	Direction
1	12-month non-partner sexual violence	1
1	Liters of alcohol consumed per capita	1
1	Log-transformed SEV scalar: Self Harm	1
1	Major depressive disorder	1
1	Population-weighted mean temperature	1
2	Healthcare access and quality index	-1
2	Population Density (150-300 ppl/sqkm, proportion)	1
2	Population Density (300-500 ppl/sqkm, proportion)	-1
2	Population Density (500-1000 ppl/sqkm, proportion)	-1
2	Population Density (over 1000 ppl/sqkm, proportion)	-1
2	Population Density (under 150 ppl/sqkm, proportion)	1
3	Education (years per capita)	-1
3	LDI (I$ per capita)	-1
3	Socio-demographic Index	-1
**Interpersonal violence**		
Level	Covariate	Direction
1	Education Relative Inequality (Gini)	1
1	Liters of alcohol consumed per capita	1
1	Log-transformed SEV scalar: Violence	1
1	Population 15 to 30 males (proportion)	1
1	Population-weighted mean temperature	1
2	Healthcare access and quality index	-1

2	Opium Cultivation (binary)	1
2	Population Density (over 1000 ppl/sqkm, proportion)	1
3	Education (years per capita)	-1
3	LDI (I$ per capita)	-1
3	Socio-demographic Index	-1
**Assault by firearm**		
Level	Covariate	Direction
1	Education Relative Inequality (Gini)	1
1	Liters of alcohol consumed per capita	1
1	Log-transformed SEV scalar: Viol Gun	1
1	Population 15 to 30 males (proportion)	1
1	Population-weighted mean temperature	1
2	Healthcare access and quality index	-1
2	Opium Cultivation (binary)	1
2	Population Density (over 1000 ppl/sqkm, proportion)	1
3	Education (years per capita)	-1
3	LDI (I$ per capita)	-1
3	Socio-demographic Index	-1
**Assault by sharp object**		
Level	Covariate	Direction
1	Education Relative Inequality (Gini)	1
1	Liters of alcohol consumed per capita	1
1	Log-transformed SEV scalar: Viol Knife	1
1	Population 15 to 30 males (proportion)	1
1[j]	Population-weighted mean temperature	1
2	Healthcare access and quality index	-1
2	Opium Cultivation (binary)	1
2	Population Density (over 1000 ppl/sqkm, proportion)	1
3	Education (years per capita)	-1
3	LDI (I$ per capita)	-1
3	Socio-demographic Index	-1
**Assault by other means**		
Level	Covariate	Direction
1	Education Relative Inequality (Gini)	1
1	Liters of alcohol consumed per capita	1
1	Log-transformed SEV scalar: Oth Viol	1
1	Population 15 to 30 males (proportion)	1
1	Population-weighted mean temperature	1
2	Healthcare access and quality index	-1
2	Opium Cultivation (binary)	1

2	Population Density (over 1000 ppl/sqkm, proportion)	1
3	Education (years per capita)	-1
3	LDI (I$ per capita)	-1
3	Socio-demographic Index	-1

a: Used at level 1 in female models, level 2 in males
b: Used at level 3 in global models, level 2 in data-rich models
c: Used at level 1 in male data-rich model. Level 2 in other three models.
d: Only used in Female global model
e: Used at level 2 in male global model, level 3 for the other three models
f: Not used in female global model
g: Only used in female global model
h: Only used in female models
i: Used at level 2 in male global mode, used at level 1 in male data-rich model. Not used in female model.
j: Used at level 2 in female, global model and level 1 for all others
k: Only used in male global model
l: Used at level 3 in male global model
m: Used at level 2 in male global model
n: Used at level 3 in the female global model
o: Only used in the female global model

Table – Injury Cause List			
**ID**	**Cause**	**Modelling Strategy**	**Covariate changes from GBD 2017**
1	Transport injuries	CODEm	Additions: Population-weighted mean temperature; Quartile on the strictness of blood-alcohol content laws of professional, general, and youth drivers; Quartile on the strictness of speed limit laws in rural and urban places; Proportion of population aged 15 to 30
1.1	Road injuries	CODEm	Additions: Population-weighted mean temperature; Quartile on the strictness of blood-alcohol content laws of professional, general, and youth drivers; Quartile on the strictness of speed limit laws in rural and urban places; Proportion of population aged 15 to 30
1.1.1	Pedestrian road injuries	CODEm	Additions: Population-weighted mean temperature; Quartile on the strictness of blood-alcohol content laws of professional, general, and youth drivers; Quartile on the strictness of speed limit laws in rural and urban places; Proportion of population aged 15 to 30
1.1.2	Cyclist road injuries	CODEm	Additions: Population-weighted mean temperature; Quartile on the strictness of

Table – Injury Cause List			
**ID**	**Cause**	**Modelling Strategy**	**Covariate changes from GBD 2017**
			blood-alcohol content laws of professional, general, and youth drivers; Quartile on the strictness of speed limit laws in rural and urban places; Proportion of population aged 15 to 30
1.1.3	Motorcyclist road injuries	CODEm	Additions: Population-weighted mean temperature; Quartile on the strictness of blood-alcohol content laws of professional, general, and youth drivers; Quartile on the strictness of speed limit laws in rural and urban places; Proportion of population aged 15 to 30
1.1.4	Motor vehicle road injuries	CODEm	Additions: Population-weighted mean temperature; Quartile on the strictness of blood-alcohol content laws of professional, general, and youth drivers; Quartile on the strictness of speed limit laws in rural and urban places; Proportion of population aged 15 to 30
1.1.5	Other road injuries	CODEm	Additions: Population-weighted mean temperature; Quartile on the strictness of blood-alcohol content laws of professional, general, and youth drivers; Quartile on the

Table – Injury Cause List			
**ID**	**Cause**	**Modelling Strategy**	**Covariate changes from GBD 2017**
			strictness of speed limit laws in rural and urban places; Proportion of population aged 15 to 30
1.2	Other transport injuries	CODEm and fatal discontinuity estimation	Additions: Population-weighted mean temperature; Quartile on the strictness of blood-alcohol content laws of professional, general, and youth drivers; Quartile on the strictness of speed limit laws in rural and urban places; Proportion of population aged 15 to 30<br><br>Dropped: Education (years per capita)
2	Unintentional injuries	Not modeled at parent cause level	
2.1	Falls	CODEm	Added: Population-weighted mean temperature; education in years per capita
2.2	Drowning	CODEm	Added: Population-weighted mean temperature
2.3	Fire, heat, and hot substances	CODEm and fatal discontinuity estimation	Added: Population-weighted mean temperature
2.4	Poisonings	CODEm	Added: Population-weighted mean temperature
2.4.1	Poisoning by carbon monoxide	CODEm	Added: Population-weighted mean temperature; summary exposure value of risk factors for poisoning by carbon monoxide, log-transformed
2.4.2	Poisoning by other means	CODEm and fatal discontinuity estimation	Added: Population-weighted mean

Table – Injury Cause List			
**ID**	**Cause**	**Modelling Strategy**	**Covariate changes from GBD 2017**
			temperature; Summary exposure value of risk factors for poisoning by other means, log-transformed
2.5	Exposure to mechanical forces	CODEm	Added: Population-weighted mean temperature
2.5.1	Unintentional firearm injuries	CODEm	Added: Population-weighted mean temperature
2.5.2	Other exposure to mechanical forces	CODEm and fatal discontinuity estimation	Added: Population-weighted mean temperature
2.6	Adverse effects of medical treatment	CODEm	Added: Alcohol liters per capita; population-weighted mean temperature; education (years per capita)
2.7	Animal contact	CODEm	Added: Population-weighted mean temperature
2.7.1	Venomous animal contact	CODEm	Added: Population-weighted mean temperature
2.7.2	Non-venomous animal contact	CODEm and fatal discontinuity estimation	Added: Population-weighted mean temperature
2.8	Foreign body	CODEm	Added: Population-weighted mean temperature Dropped: Population of people living at greater than 1500 meters (proportion); Population density over 1,000 per square kilometer (proportion); Population density under 150 per square kilometer (proportion); Population of people living under 100 meters elevation (proportion)
2.8.1	Pulmonary aspiration and foreign body in airway	CODEm	Added: Population-weighted mean temperature;

Table – Injury Cause List			
**ID**	**Cause**	**Modelling Strategy**	**Covariate changes from GBD 2017**
			education (years per capita)
2.8.2	Foreign body in other body part	CODEm	Added: Population-weighted mean temperature<br>Dropped: Population of people living at greater than 1500 meters (proportion); Population density over 1,000 per square kilometer (proportion); Population density under 150 per square kilometer (proportion); Population of people living under 100 meters elevation (proportion)
2.9	Environmental exposure to heat and cold	CODEm and fatal discontinuity estimation	
2.10	Exposure to forces of nature	Fatal discontinuity estimation	
2.11	Other unintentional injuries	CODEm and fatal discontinuity estimation	Added: Population-weighted mean temperature<br><br>Dropped: Population living at over 1,500 meters elevation (proportion); Population living under 100 meters elevation (proportion)
3	Self-harm and interpersonal violence	Not modeled at parent cause level	
3.1	Self-harm	CODEm	Population-weighted mean temperature; 12-month non-partner sexual violence
3.1.1	Self-harm by firearm	CODEm	Population-weighted mean temperature; 12-month non-partner sexual violence
3.1.2	Self-harm by other specified means	CODEm	Population-weighted mean temperature; 12-

Table – Injury Cause List			
**ID**	**Cause**	**Modelling Strategy**	**Covariate changes from GBD 2017**
			month non-partner sexual violence
3.2	Interpersonal violence	CODEm	Population-weighted mean temperature; Education relative inequality (Gini); Proportion of population males 15 to 30 years old
3.2.1	Physical violence by firearm	CODEm and fatal discontinuity estimation	Population-weighted mean temperature; Education relative inequality (Gini); Proportion of population males 15 to 30 years old
3.2.2	Physical violence by sharp object	CODEm and fatal discontinuity estimation	Population-weighted mean temperature; Education relative inequality (Gini); Proportion of population males 15 to 30 years old
3.2.3	Physical violence by other means	CODEm and fatal discontinuity estimation	Population-weighted mean temperature; Education relative inequality (Gini); Proportion of population males 15 to 30 years old
3.3	Conflict and terrorism	Fatal discontinuity estimation	
3.4	Executions and police conflict	CODEm and fatal discontinuity estimation	Population-weighted mean temperature; Proportion of population males 15 to 30 years old

References

1 Lozano R, Naghavi M, Foreman K, *et al.* Global and regional mortality from 235 causes of death for 20 age groups in 1990 and 2010: a systematic analysis for the Global Burden of Disease Study 2010. *The Lancet* 2012; **380**: 2095–128.

2 Global, regional, and national age–sex specific all-cause and cause-specific mortality for 240 causes of death, 1990–2013: a systematic analysis for the Global Burden of Disease Study 2013. *The Lancet* 2015; **385**: 117–71.

3 Global, regional, and national life expectancy, all-cause mortality, and cause-specific mortality for 249 causes of death, 1980-2015: a systematic analysis for the Global Burden of Disease Study 2015. The Lancet 2016; **388**: 1459-1544.

4 Murray CJL, Lopez AD, Harvard School of Public Health, World Health Organization, World Bank. The global burden of disease: a comprehensive assessment of mortality and disability from diseases, injuries, and risk factors in 1990 and projected to 2020. Cambridge, MA: Published by the Harvard School of Public Health on behalf of the World Health Organization and the World Bank : Distributed by Harvard University Press, 1996.

5 Matzopoulos R, Prinsloo M, Wyk VP, Gwebushe N, Mathews S, et al. Injury-related mortality in South Africa: a retrospective descriptive study of postmortem investigations. *Bull World Health Organ* 2015; **93**: 303–13.

6 Feldman JM, Gruskin S, Coull BA, Krieger N (2017) Quantifying underreporting of law-enforcement-related deaths in United States vital statistics and news-media-based data sources: A capture–recapture analysis. PLOS Medicine 14(10): e1002399.
